# Supplementary material for: Straightforward Access to a New Class of Dual DYRK1A/CLK1 Inhibitors Possessing a Simple Dihydroquinoline Core
Source: Molecules. 2022 Dec 21;28(1):36. doi: 10.3390/molecules28010036 (PMC9822041; doi:10.3390/molecules28010036)

Straightforward Access to a New Class of Dual DYRK1A/CLK1 Inhibitors Possessing a Simple Dihydroquinoline Core

Mihaela-Liliana Ţînţaş [1], Ludovic Peauger [2], Florent Alix [2], Cyril Papamicaël [1,*], Thierry Besson [1], Jana Sopková-de Oliveira Santos [3], Vincent Gembus [2,*], Vincent Levacher [1,*]

[1] INSA Rouen Normandie, Univ Rouen Normandie, CNRS UMR 6014 COBRA, FR 3038, F-76000 Rouen, France
[2] VFP Therapies, 15 rue François Couperin, 76000 Rouen, France
[3] UNICAEN, CERMN (Centre d'Etudes et de Recherche sur le Médicament de Normandie), Normandie Univ., Bd becquerel, F-14032 Caen, France
* Correspondence: vincent.levacher@insa-rouen.fr; Tel.: +33-023-552-2485 (V.L.), cyril.papamicael@insa-rouen.fr (C.P.), gembusv@hotmail.fr (V.G.)

Contents

I - In vitro studies 2
I-1 DYRK1A Human CMGC Kinase Enzymatic Radiometric Assay [Km ATP] 2
I-2 CLK1 Human CMGC Kinase Enzymatic Radiometric Assay [Km ATP] 2
I-3 Solubility of **1p** 2
I-4 Blood-Brain Barrier Permeability (PAMPA-BBB) of **1p** 2
II – Stability/oxidation studies 3
III – In vitro DPPH radical scavenging assay 4
IV – ^{1}H NMR evidence of the oxidation of **1p** to **2p** in the presence of DPPH$^{\bullet}$ 5
V – ADME results for **1p** 6
VI - NMR spectra 7

I - In vitro studies[1,2,3]

I-1 DYRK1A Human CMGC Kinase Enzymatic Radiometric Assay [Km ATP]

*h*DYRK1A is incubated with 8 mM MOPS pH 7.0, 0.2 mM EDTA, 50 µM RRRFRPASPLRGPPK, 10 mM MgAcetate and [gamma-33P]-ATP (specific activity and concentration as required). The reaction is initiated by the addition of the Mg/ATP mix. After incubation for 40 min at 20 °C, the reaction is stopped by the addition of phosphoric acid to a concentration of 0.5%. An aliquot of the reaction is then spotted onto a filter and washed four times for 4 minutes in 0.425% phosphoric acid and once in methanol prior to drying and scientillation counting. Staurosporine was used as a control inhibitor.

I-2 CLK1 Human CMGC Kinase Enzymatic Radiometric Assay [Km ATP]

*h*CLK1 is incubated with 8 mM MOPS pH 7.0, 0.2 mM EDTA, 1 mM sodium orthovanadate, 5 mM sodium 6- glycerophosphate, 200 uM ERMRPRKRQGSVRRRV, 10 mM MgAcetate and [gamma-33P]-ATP (specific activity and concentration as required). The reaction is initiated by the addition of the Mg/ATP mix. After incubation for 40 minutes at 20 °C, the reaction is stopped by the addition of phosphoric acid to a concentration of 0.5%. An aliquot of the reaction is then spotted onto a filter and washed four times for 4 minutes in 0.425% phosphoric acid and once in methanol prior to drying and scintillation counting. Staurosporine was used as a control inhibitor.

I-3 Solubility of **1p**

The analysis was performed using a LC system (Thermo U3000) under UV detection. HPLC analysis was performed using a Hypersil GOLD C18 (150*2.1 mm, 3 µm); the gradient (CAN in 0.1% formic acid in water) and the mobile phase (flow rate 400 $mL.min^{-1}$) used are determined in order to detect the compound of interest with satisfying retention time and peak shape (run time 15 min, column temperature 40 °C). Acquisition and analysis of data were performed with Chromeleon software. 100 µL of 1 mM solution in DMSO of the compound are diluted to 10 mL mark with PBS pH 7.4 (in triplicate). The tubes were shaken 24 h at 20 °C, then centrifuged at 10000 rpm for 5 min. Then, the mixtures were filtered over 0.45 µm filters and 10 µL of samples were subjected to HPLC analysis. A calibration curve was used to calculate the solubility.

Compound	Solubility
**1p**	0.6 mM

I-4 Blood-Brain Barrier Permeability (PAMPA-BBB) of **1p**

Assay description

CNS screening of candidate drug molecules is done using a PAMPA (Parallel Artificial Membrane Permeability Assay) assay to model blood-brain barrier (BBB) permeability. The PAMPA-BBB test is performed according to the methodology using the BBB-Pampa Explorer® kit (pION Inc, Woburn, MA, USA). It consists in the measurement of the flow rate of a compound from a compartment to another through an artificial membrane at pH=7.4.

Material and methods

Compounds were diluted to 20 mM in DMSO and then at 100 µM in pH 7.4 Prisma HT buffer (pION). Then, 200 µL of this solution were added to each well of the donor plate (n = 6). The filter membrane on the acceptor plate was coated with 5 µL of the BBB-1 lipid (pION)

---

[1] Davies S.P.; Reddy H.; Caivano M.; Cohen P. *Biochem. J.* **2000**, *351(Pt 1)*, 95-105.

[2] Gao Y.; Davies S.P.; Augustin M.; Woodward A.; Patel U.A.; Kovelman R.; Harvey K.J. *Biochem. J.* **2013**, *451*, 313-328.

[3] Peauger L.; Azzouz R.; Gembus V.; Ţînţaş M.-L.; Sopková-de Oliveira Santos J.; Bohn, P.; Papamicaël C.; Levacher V. *J. Med. Chem.* **2017**, *60*, 5909-5926.

formulation and to each well of the acceptor plate, 200 µL of brain sink buffer (BSB, pION) was added. The acceptor filter plate was placed on top of the donor plate to form a “sandwich”. The sandwich was incubated at room temperature for 4 h without stirring. The sandwich is then separated and the UV-vis spectra were measured in the reference, acceptor and donor plates using a microplate reader (Tecan infinite® M200). For each compound, -logPe was calculated using the PAMPA Explorer® software v. 3.7 (pION). The references used are theophylline (250 µM), corticosterone (100 µM).

Results

Prediction of CNS positive (CNS+) and CNS negative (CNS-) compounds using PAMPA model[4]

Compound	Tested concentration	Pe (10^{-6} cm.s^{-1})	Prediction
**1p**	100 µM	20.0±0.9	CNS+
Theophylline	250 µM	0.5±0.1	CNS-
Corticosterone	100 µM	20.1±0.8	CNS+

II – Stability/oxidation studies

The *in vitro* stability of compound **1p** was studied in PBS buffer (pH 7.4), in 100% of rehydrated human plasma with PBS buffer (pH 7.4), in 0.2 % and 2% NAD^+ solution in PBS, in freshly prepared 20% mice brain homogenate in PBS (pH 7.4), in 0.1% hydrogen peroxide in PBS (pH 7.4) and in 0.1% (-)-Riboflavin solution in PBS. The incubation was initiated by the addition of compound **1p** (10 mM in DMSO) to the above solutions to obtain a final concentration of 25 µM. The assays were performed at 37 °C and conducted in duplicate. Samples (10 µL) in PBS, NAD^+, riboflavin or hydrogen peroxide solutions were directly analyzed by HPLC-UV. Samples (10 µL) in human plasma and in mice brain homogenate were subjected to vortex mixing for 1 min and then centrifugation for 10 min at 13,000 rpm in order to deproteinize. Samples (10 µL) of the resulting supernatants were withdrawn and analyzed by HPLC-UV to determine the percentage of remaining prodrug **1p** and oxidized compound **2p**. The analyses were performed using a LC system (Thermo U3000) under UV detection. HPLC analysis was performed using a Hypersil Gold TM C18 (150*2.1 mm, 3µm) with the gradient (ACN in 0.1% formic acid water), a flow rate of 0.4 mL.min^{-1} and column temperature at 40 °C. Acquisition and analysis of data were performed with Chromeleon software.

[4] Di L.; Kerns E.H.; Fan K.; McConnell O.J.; Carter G.T. *Eur. J. Med. Chem.* **2003**, *38*, 223-232.

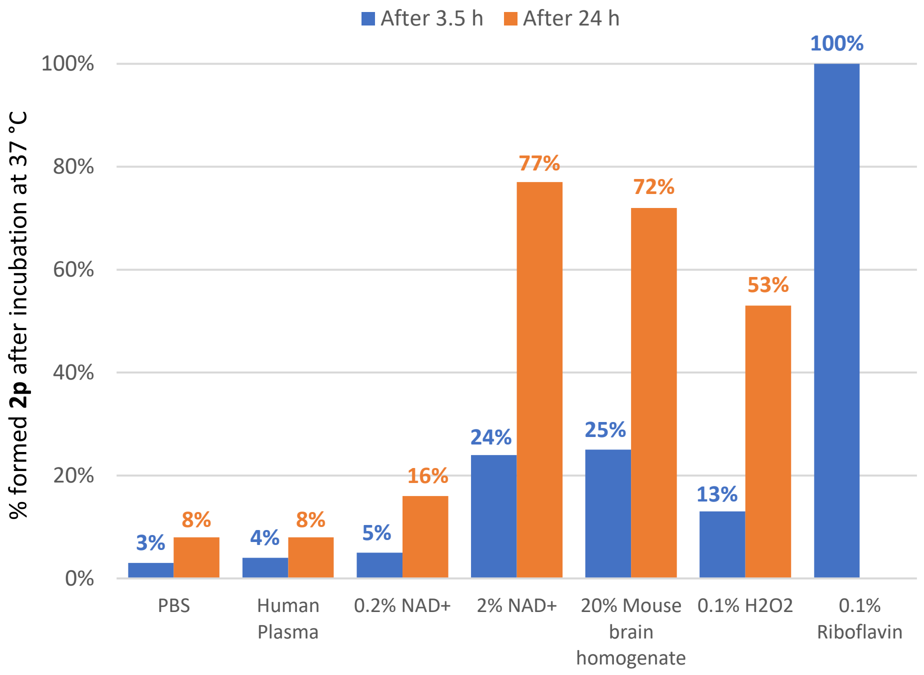

Figure 1. In vitro stability of **1p** in various non-oxidazing and oxidazing media.

III – In vitro DPPH radical scavenging assay

DPPH radical scavenging activity was determined according to the method described by Blois.[5] 0.5 mL of 0.1 mM DPPH radical solution in methanol was mixed with 1.5 mL of various concentrations in methanol of **1p**. The mixture was then thoroughly vortexed for 5s and each antioxidant-DPPH radical reaction mixture is kept in the test tubes in the dark at 37 °C for 40 min (time point by which all reactions should have reached steady state) and transferred to a cuvette for absorbance measurement at 517 nm. For the baseline control, 1.5 mL of methanol was used. Mean values were obtained from triplicate experiments. Percentage of inhibition was calculated using the equation $[(A_0-A_1)/A_0] \times 100$ where A_0 was the absorbance of the control and A_1 was the absorbance in the presence of the compound. Percent inhibition was plotted *vs* concentration; the EC 50 values were determined using OriginPro®.

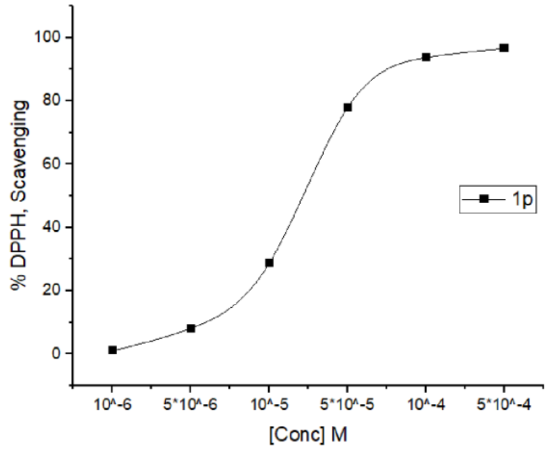

Figure 2. DPPH radical scavenging activity of compound **1p**. Data are presented as mean ± SD of each of three replicates.

[5] Blois M.S. *Nature*, **1958**, *181*, 1199-2000.

IV – ^{1}H NMR evidence of the oxidation of **1p** to **2p** in the presence of DPPH•

To a solution of DPPH• (2 mg, 5.1 µmol) in degassed DMSO-d_6 (0.5 mL) was added a solution of **1p** (2.5 mg, 10.2 µmol) in degassed DMSO-d_6 (0.5mL). The mixture was then thoroughly vortexed for 5s and kept in the dark all the time of the reaction. ^{1}H NMR analysis was performed immediately and the recorded spectra were compared to **2p** and **1p** ^{1}H NMR without DPPH radical.

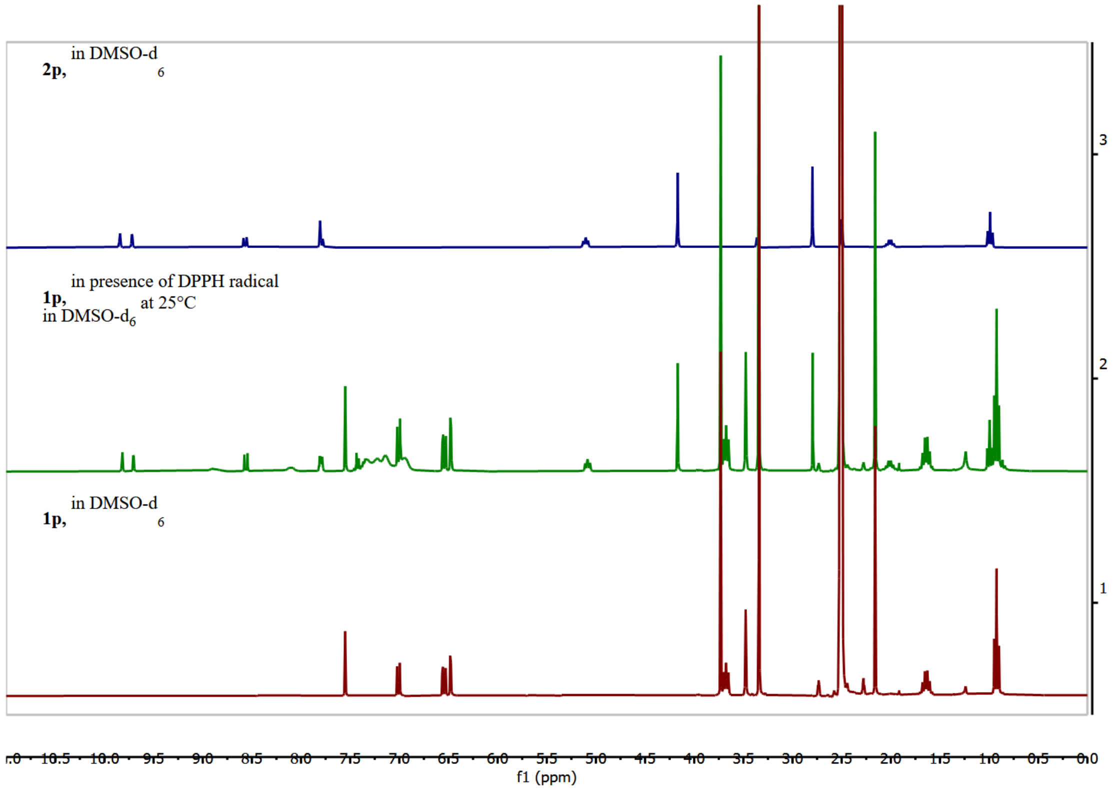

Figure 3. ^{1}H NMR spectra in DMSO-d_6 of **2p** (top), **1p** in the presence of DPPH radical (middle) and **1p** (bottom).

V – ADME results for **1p**

Results obtained from the free website swissadme.ch (24th of september 2022)

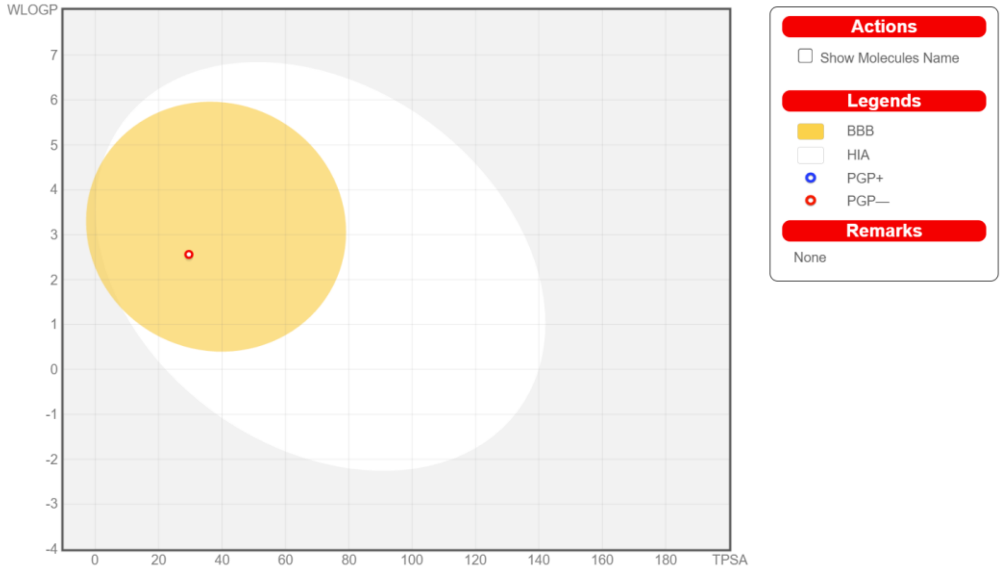

Molecule 1

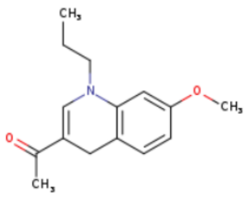

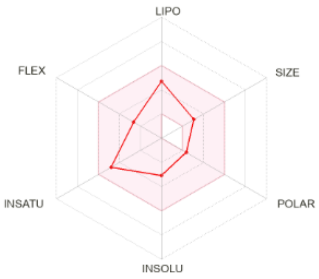

SMILES CCCN1C=C(Cc2c1cc(OC)cc2)C(=O)C

Physicochemical Properties	
Formula	C15H19NO2
Molecular weight	245.32 g/mol
Num. heavy atoms	18
Num. arom. heavy atoms	6
Fraction Csp3	0.40
Num. rotatable bonds	4
Num. H-bond acceptors	2
Num. H-bond donors	0
Molar Refractivity	76.69
TPSA	29.54 Å²

Lipophilicity	
Log $P_{o/w}$ (iLOGP)	2.86
Log $P_{o/w}$ (XLOGP3)	2.74
Log $P_{o/w}$ (WLOGP)	2.56
Log $P_{o/w}$ (MLOGP)	2.02
Log $P_{o/w}$ (SILICOS-IT)	2.97
Consensus Log $P_{o/w}$	2.63

Water Solubility	
Log *S* (ESOL)	-3.07
Solubility	2.09e-01 mg/ml ; 8.51e-04 mol/l
Class	Soluble
Log *S* (Ali)	-3.01
Solubility	2.37e-01 mg/ml ; 9.66e-04 mol/l
Class	Soluble
Log *S* (SILICOS-IT)	-3.98
Solubility	2.56e-02 mg/ml ; 1.04e-04 mol/l
Class	Soluble

Pharmacokinetics	
GI absorption	High
BBB permeant	Yes
P-gp substrate	No
CYP1A2 inhibitor	Yes
CYP2C19 inhibitor	Yes
CYP2C9 inhibitor	No
CYP2D6 inhibitor	No
CYP3A4 inhibitor	No
Log K_p (skin permeation)	-5.85 cm/s

Druglikeness	
Lipinski	Yes; 0 violation
Ghose	Yes
Veber	Yes
Egan	Yes
Muegge	Yes
Bioavailability Score	0.55

Medicinal Chemistry	
PAINS	0 alert
Brenk	0 alert
Leadlikeness	No; 1 violation: MW<250
Synthetic accessibility	2.95

VI - NMR spectra

1-(1-Methyl-1,4-dihydroquinolin-3-yl)ethanone (**1a**)

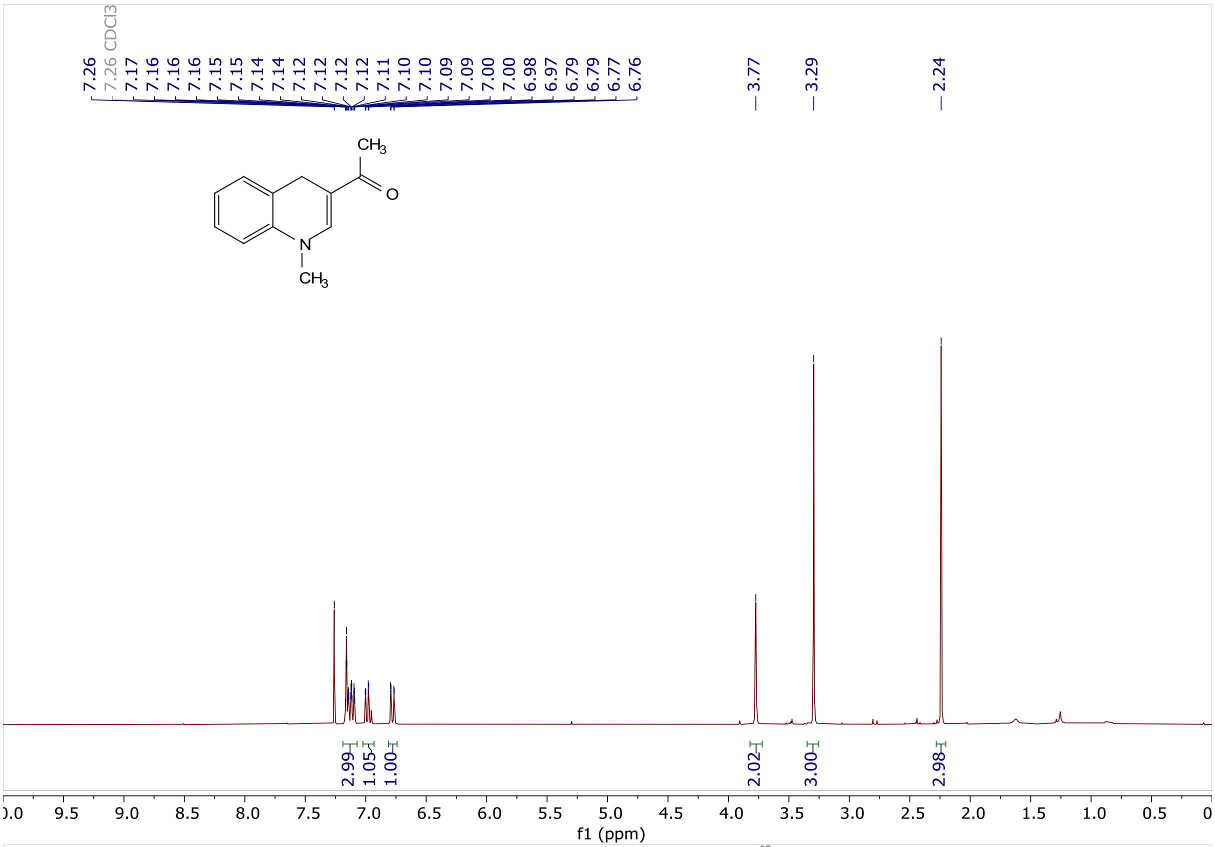

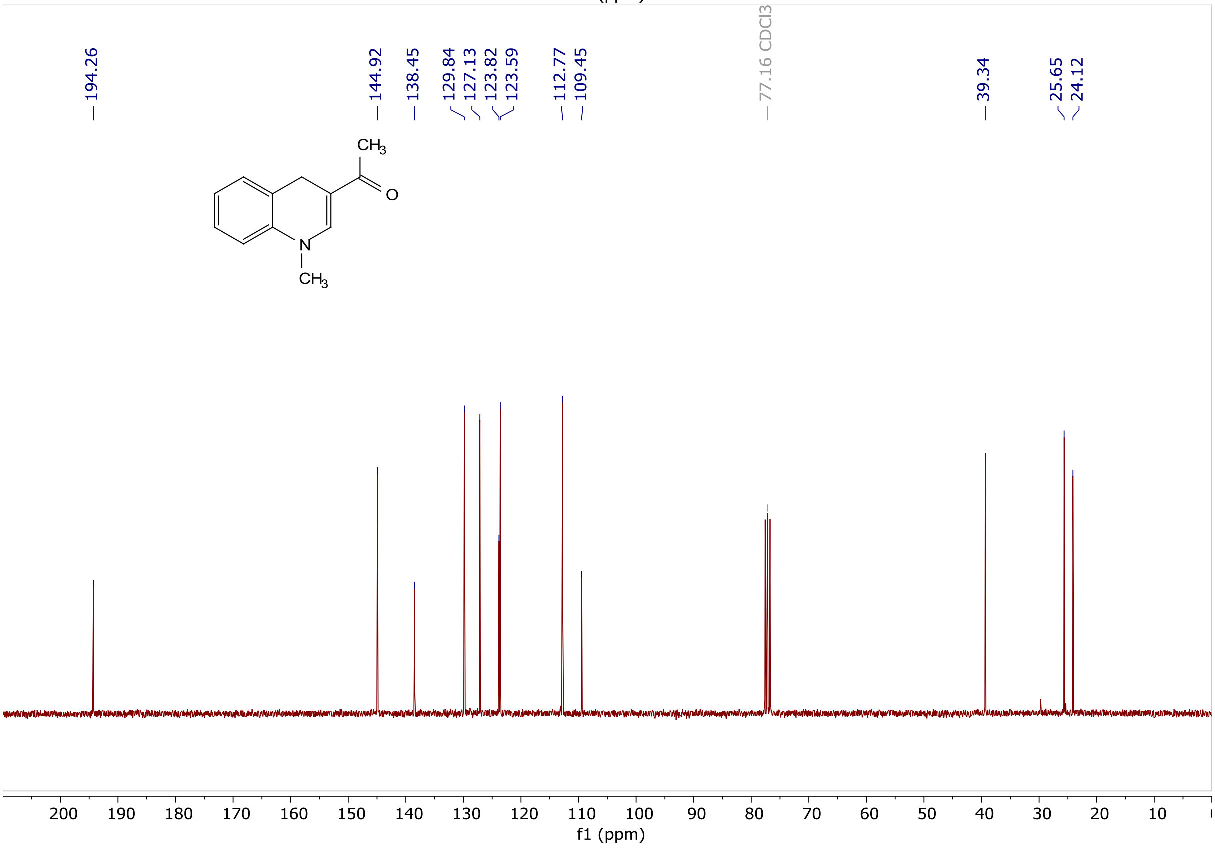

1-(1-Methyl-7-(trifluoromethyl)-1,4-dihydroquinolin-3-yl)ethanone (**1b**)

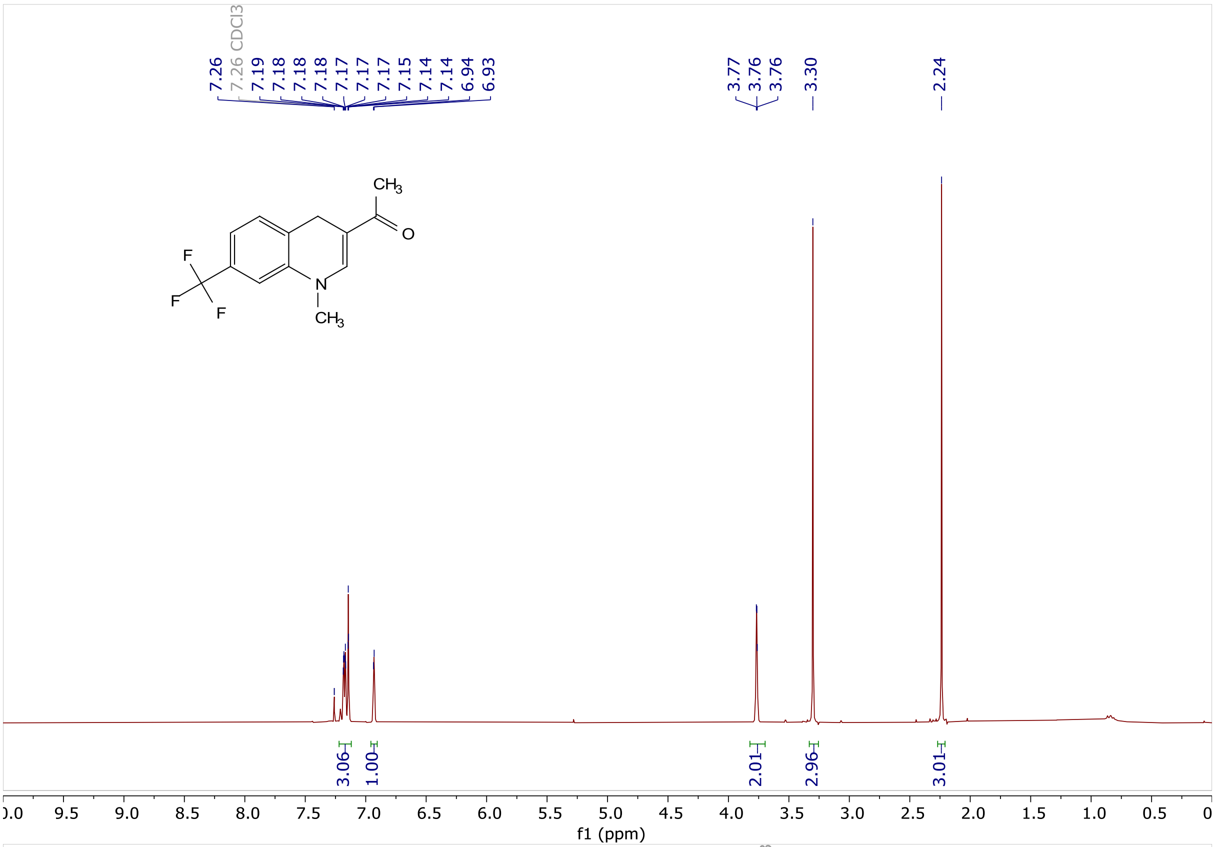

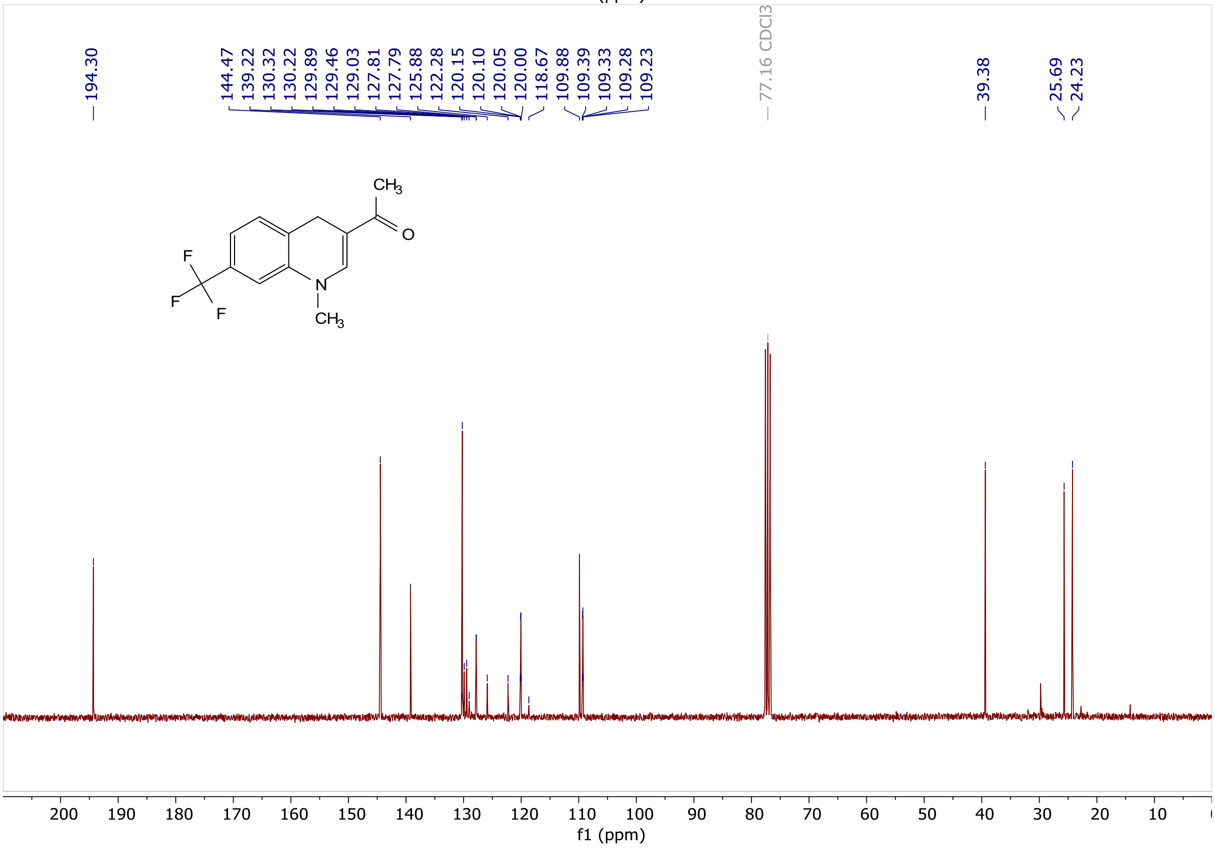

1-(7-(Dimethylamino)-1-methyl-1,4-dihydroquinolin-3-yl)ethanone (**1c**)

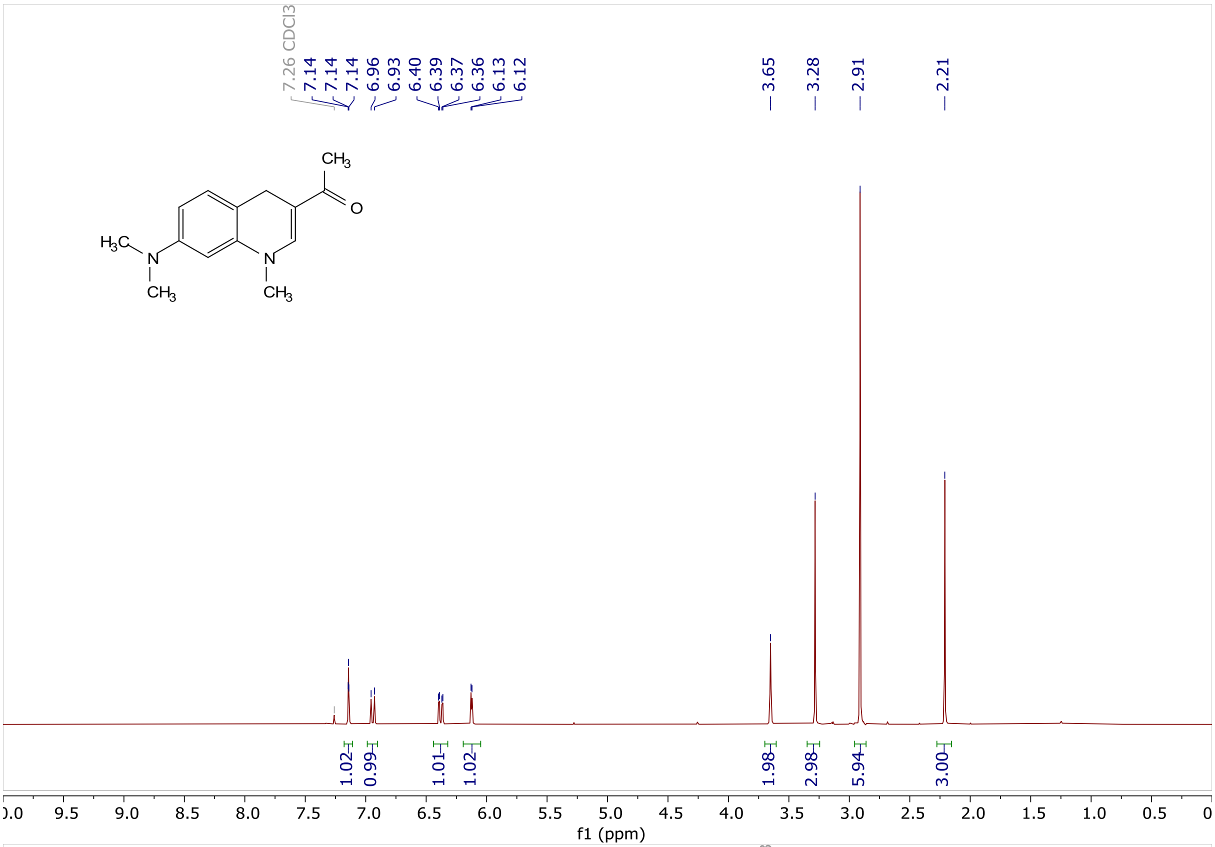

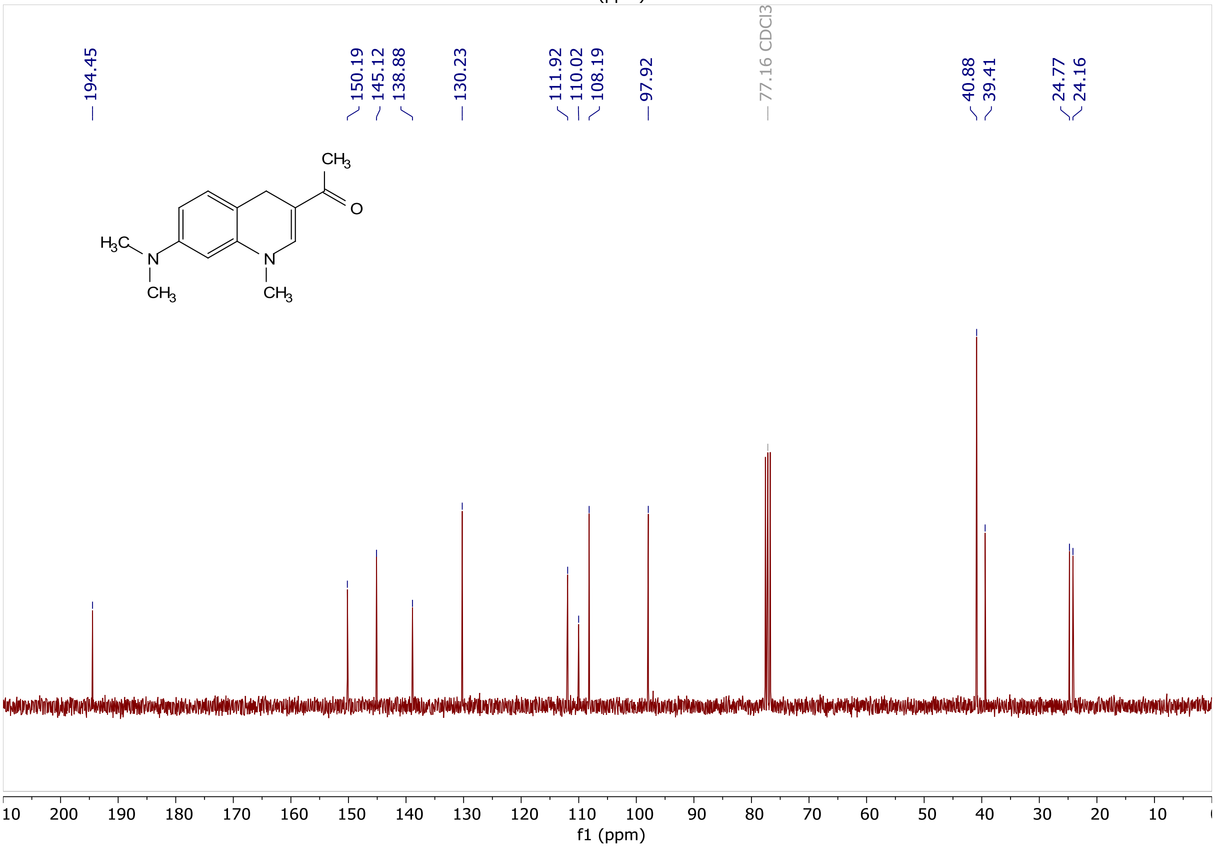

1-(7-Bromo-1-methyl-1,4-dihydroquinolin-3-yl)ethanone (**1d**)

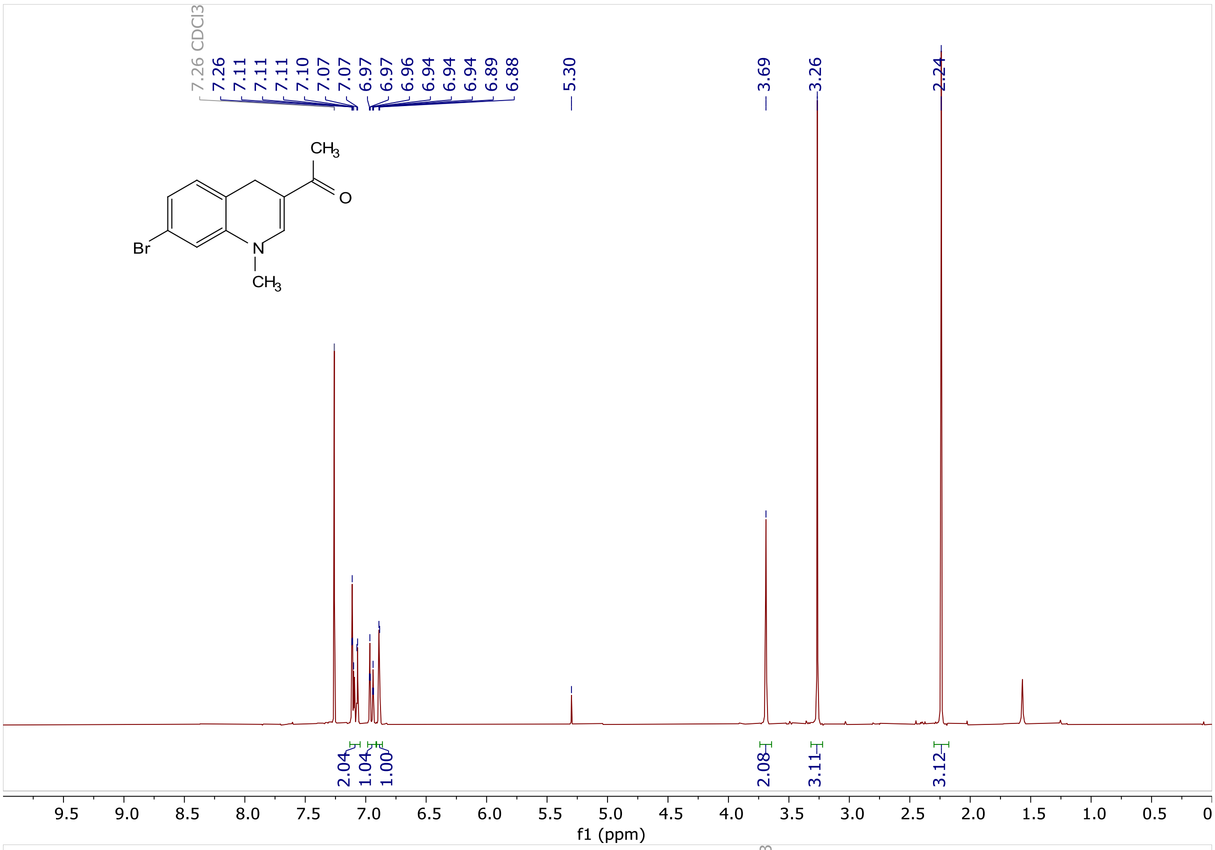

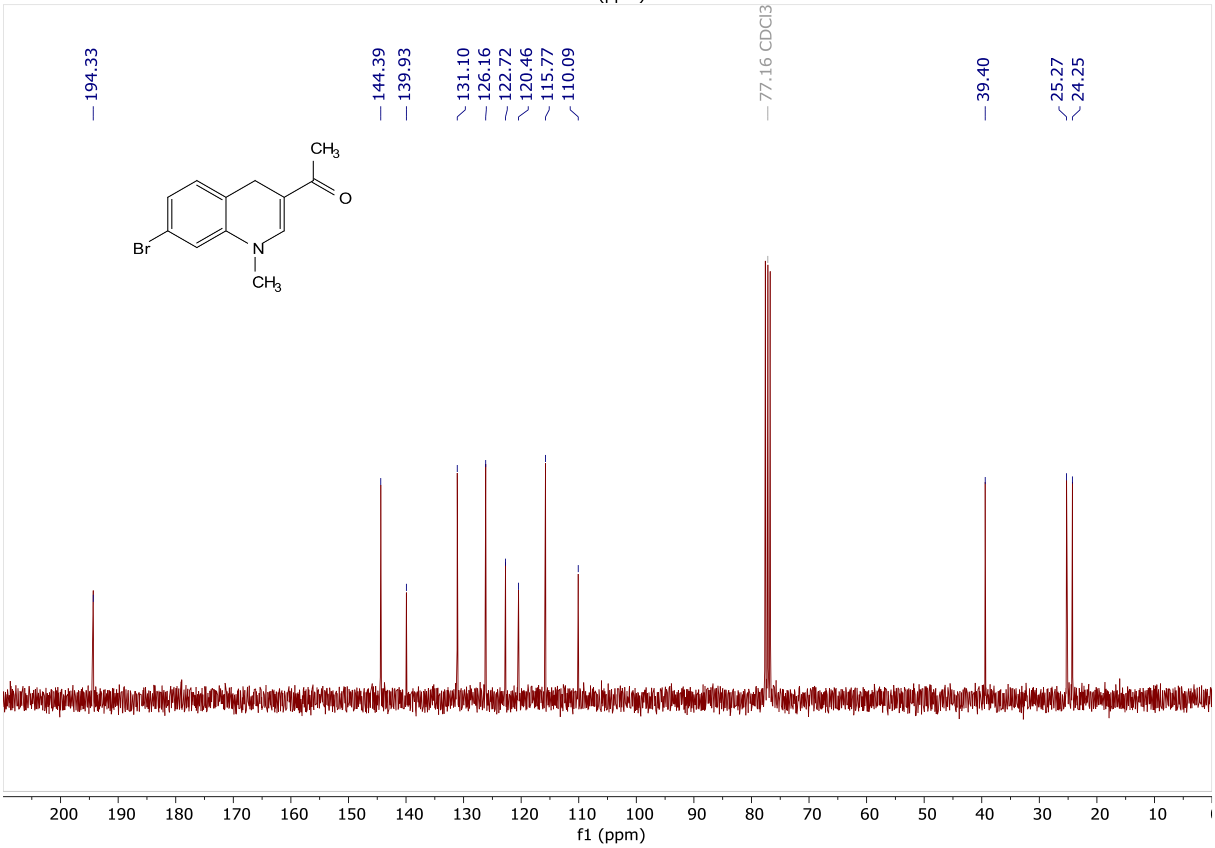

1-(6-Chloro-1-methyl-1,4-dihydroquinolin-3-yl)ethanone (**1e**)

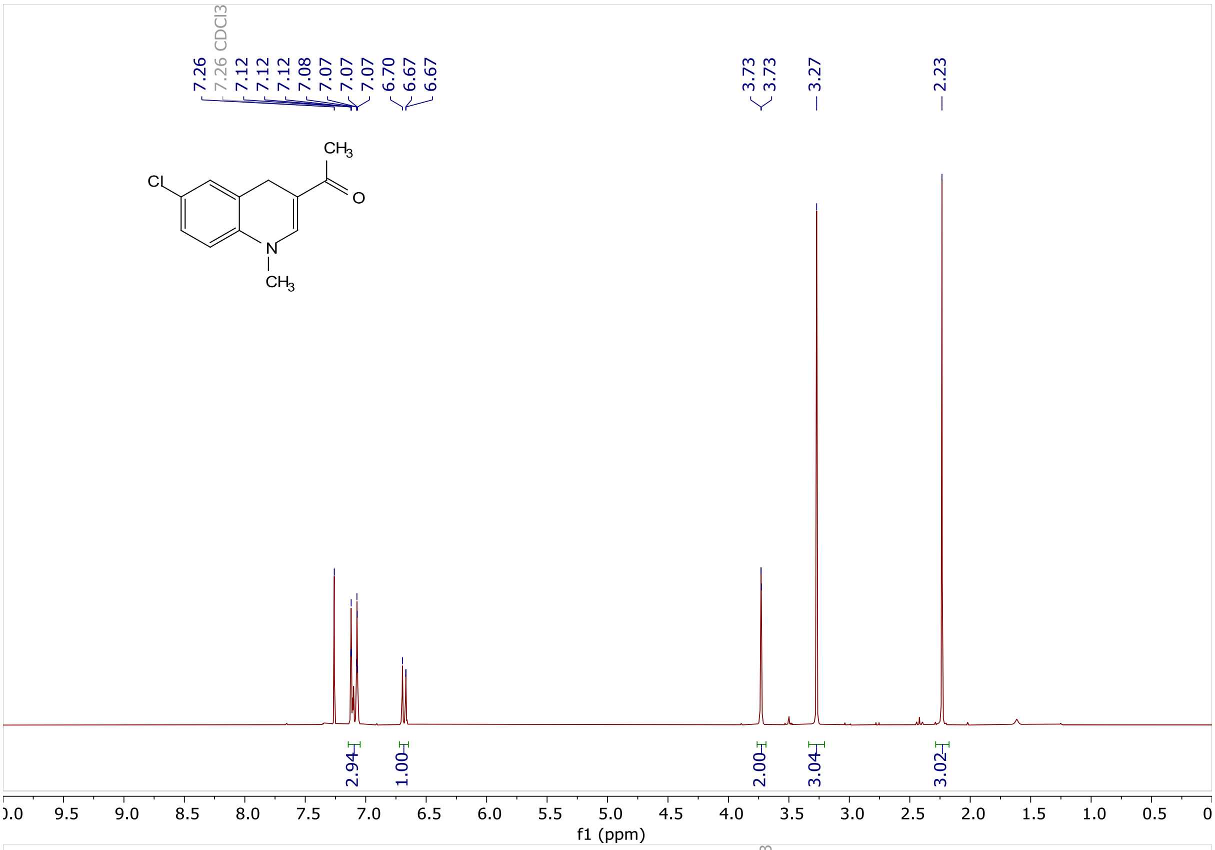

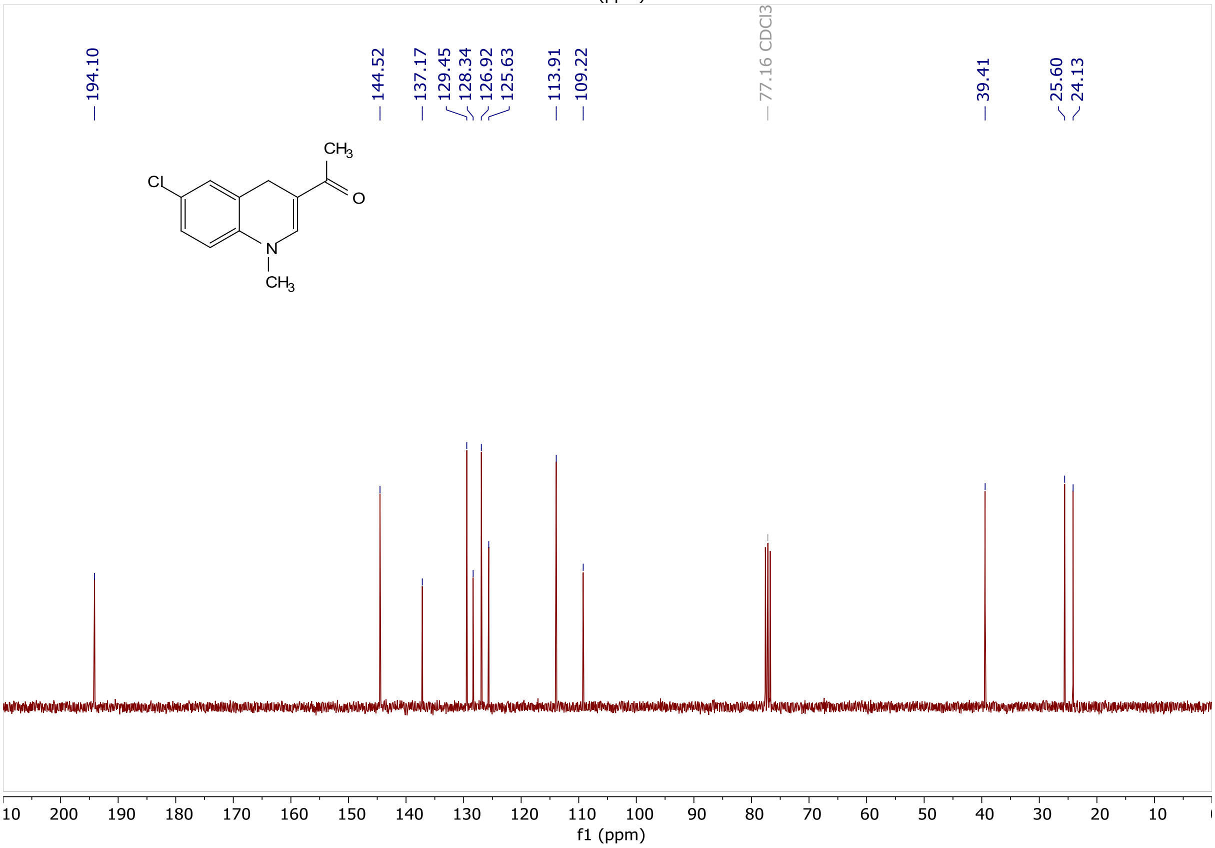

1-(6,7-Dimethoxy-1-methyl-1,4-dihydroquinolin-3-yl)ethanone (**1f**)

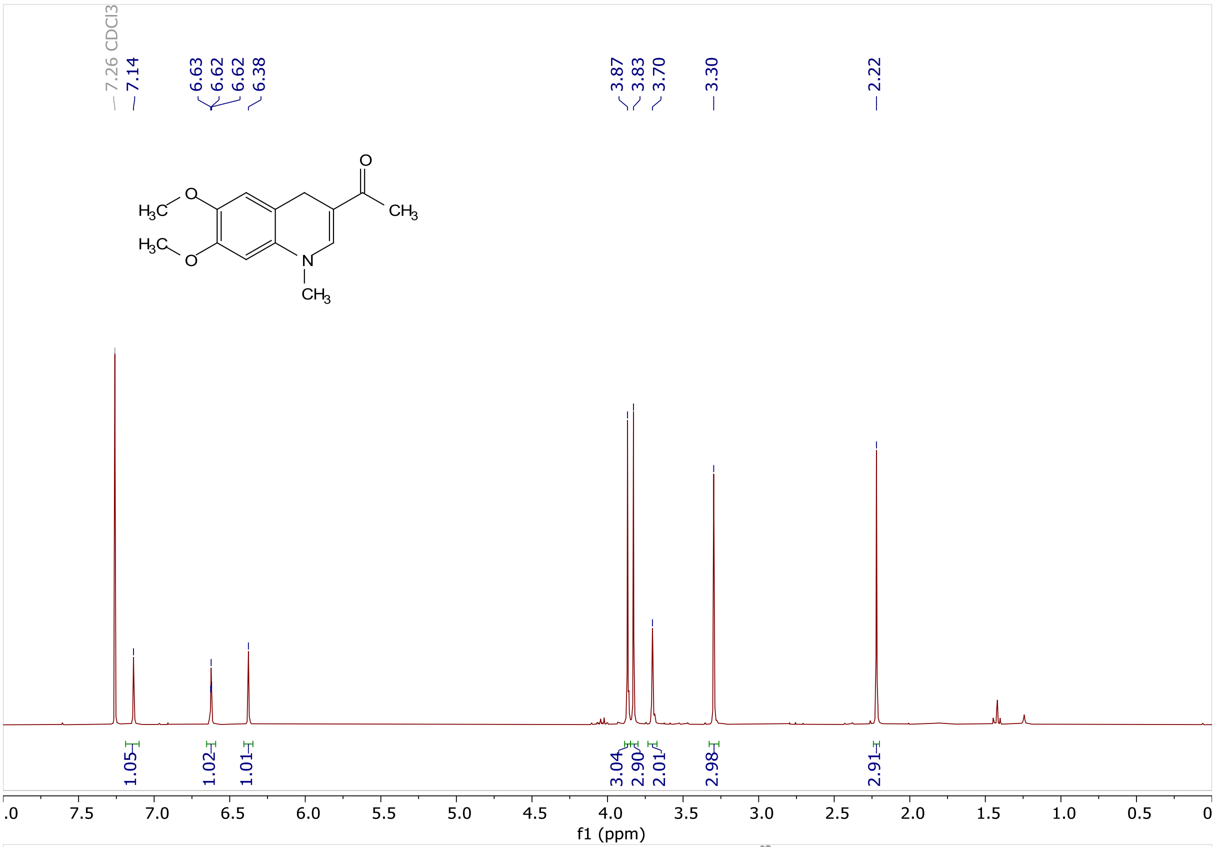

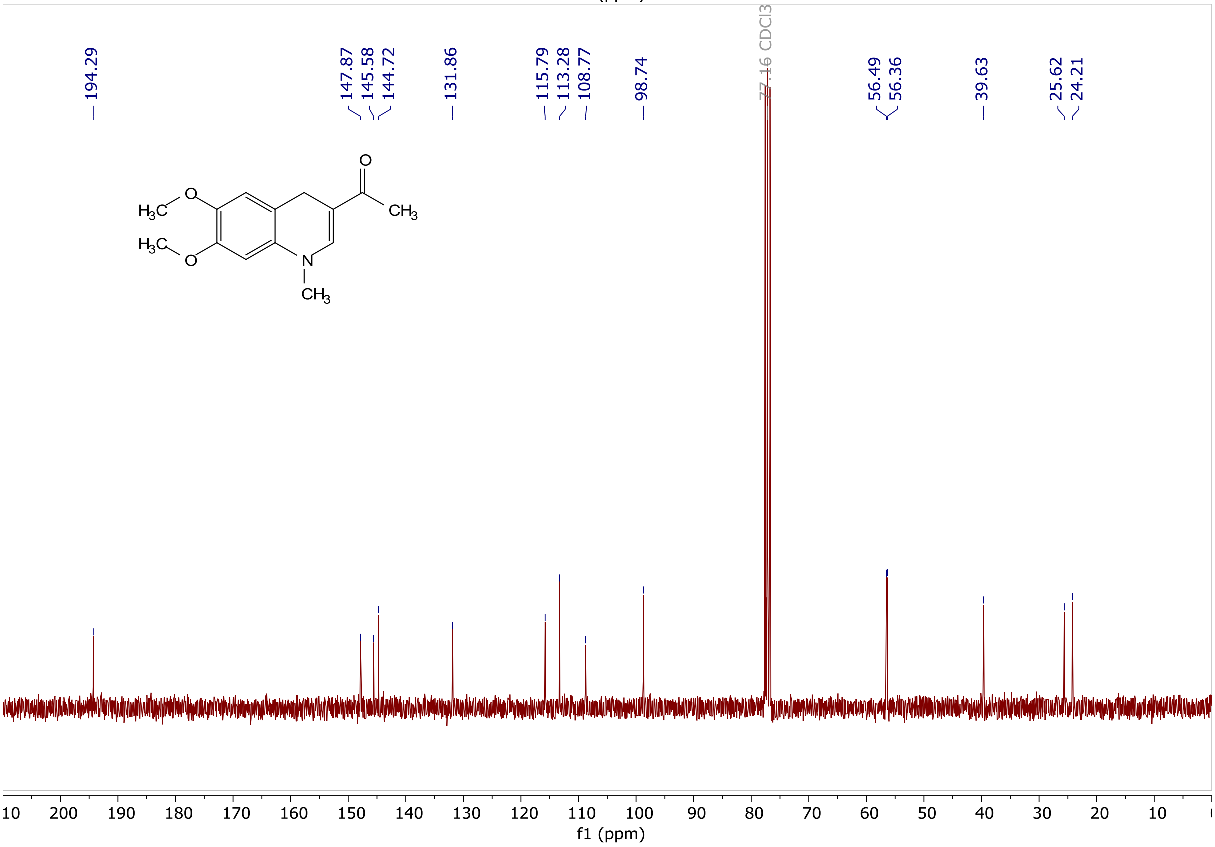

1-(5-Methyl-5,8-dihydro-[1,3]dioxolo[4,5-g]quinolin-7-yl)ethanone (**1g**)

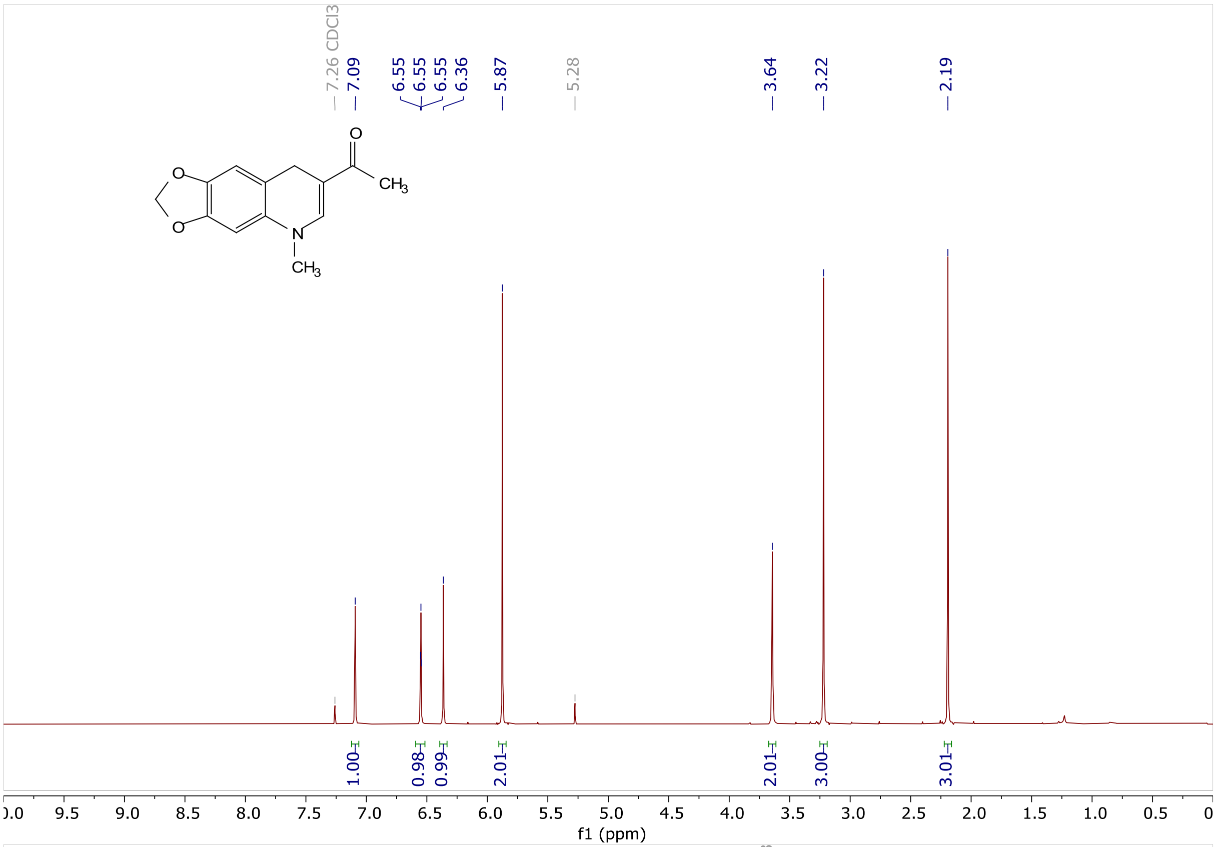

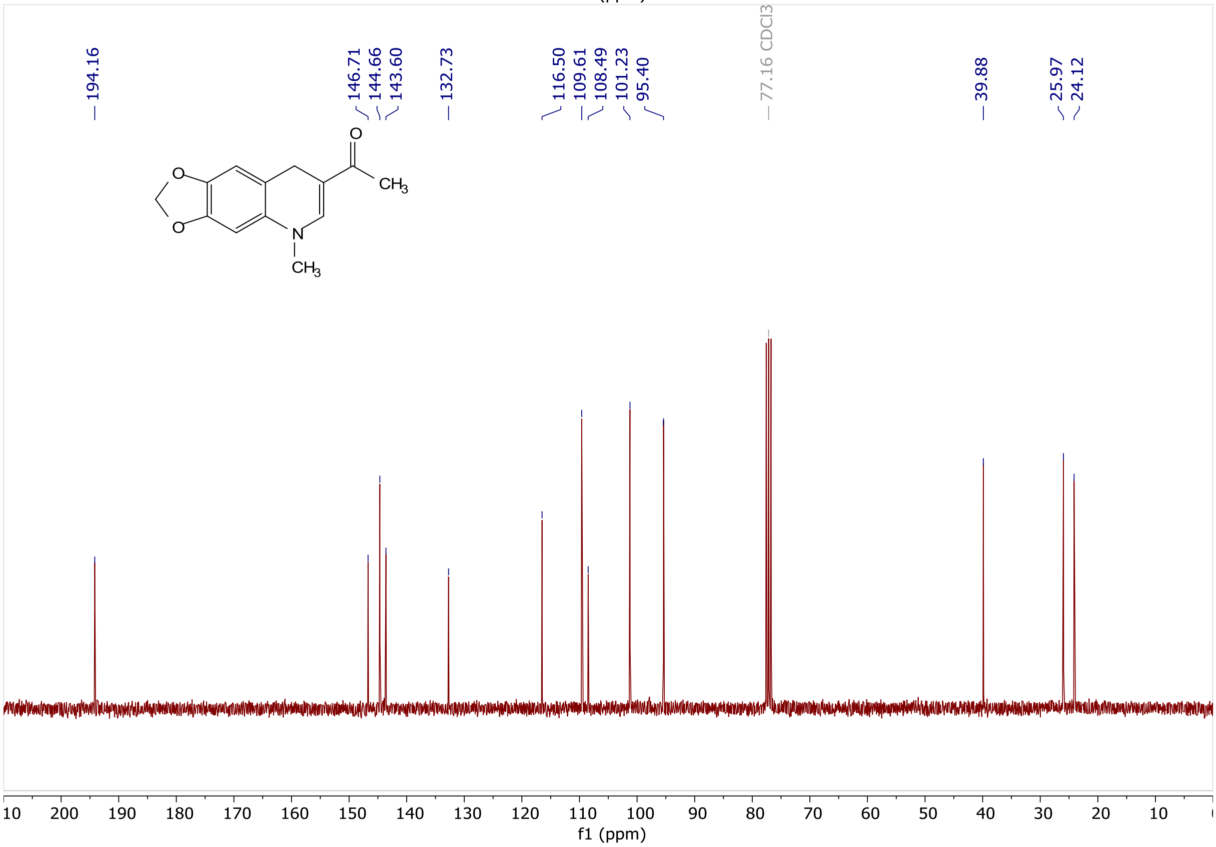

1-(7-Methoxy-1-methyl-1,4-dihydroquinolin-3-yl)ethanone (**1h**)

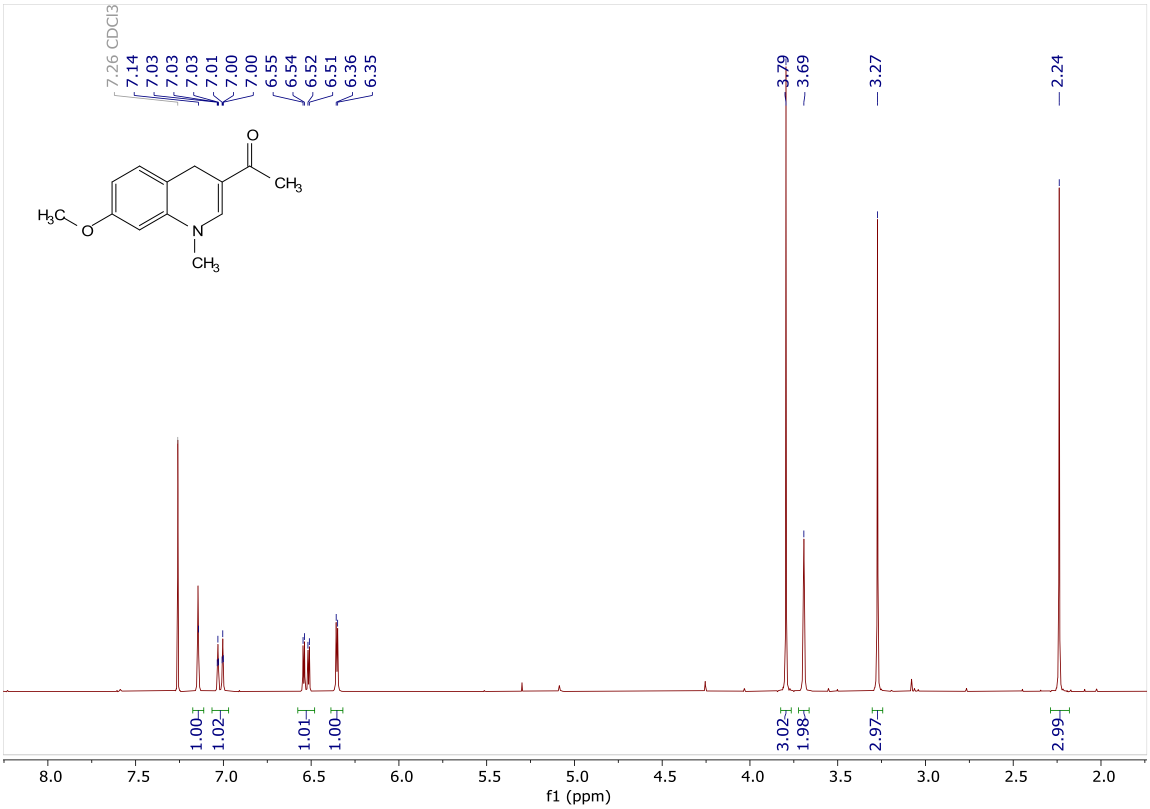

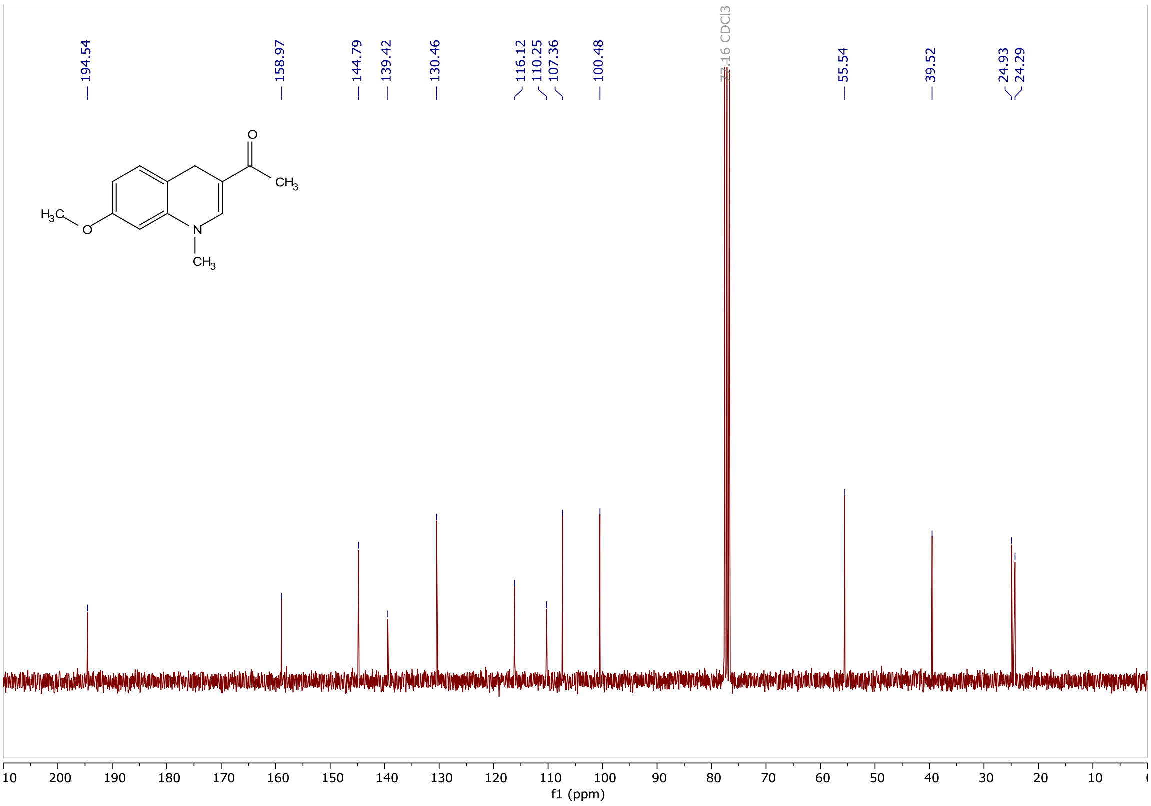

7-Methoxy-1-methyl-1,4-dihydroquinoline-3-carbonitrile (**1i**)

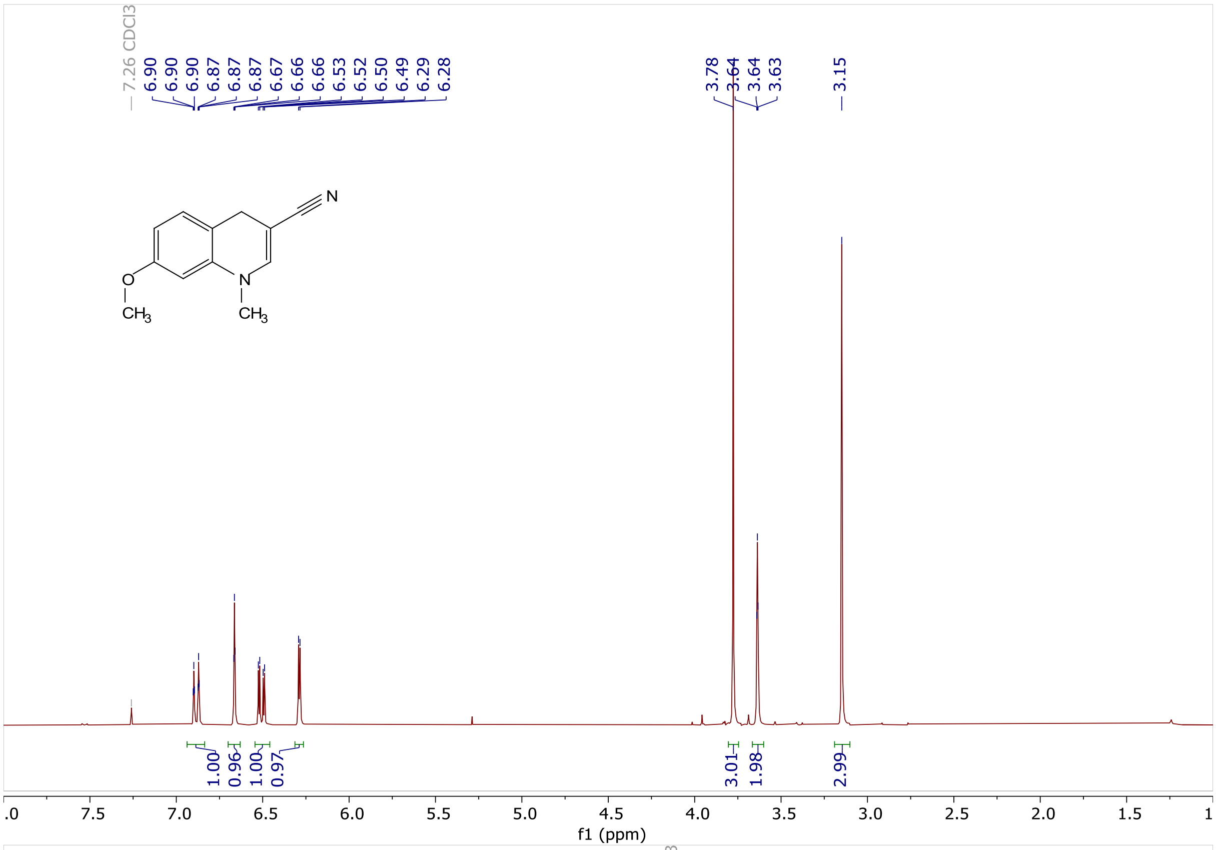

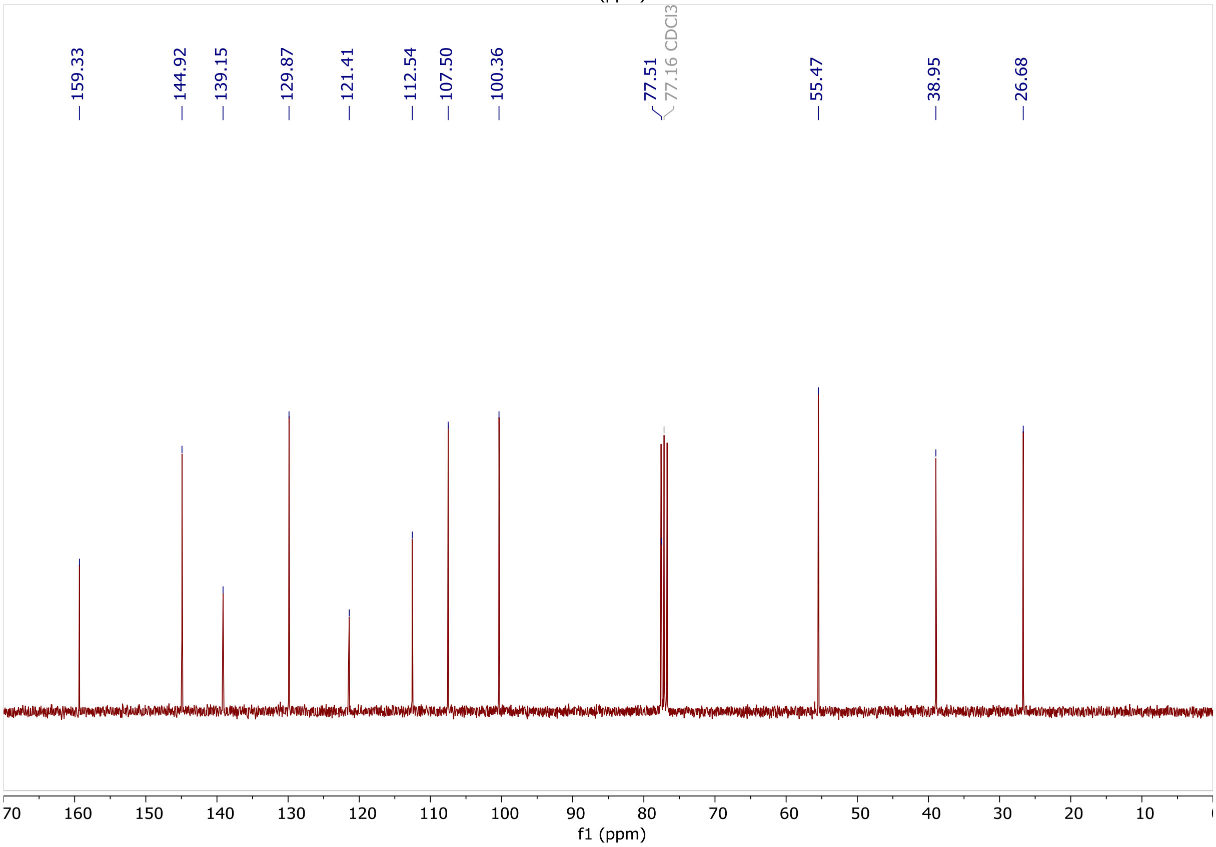

Methyl 7-methoxy-1-methyl-1,4-dihydroquinoline-3-carboxylate (**1j**)

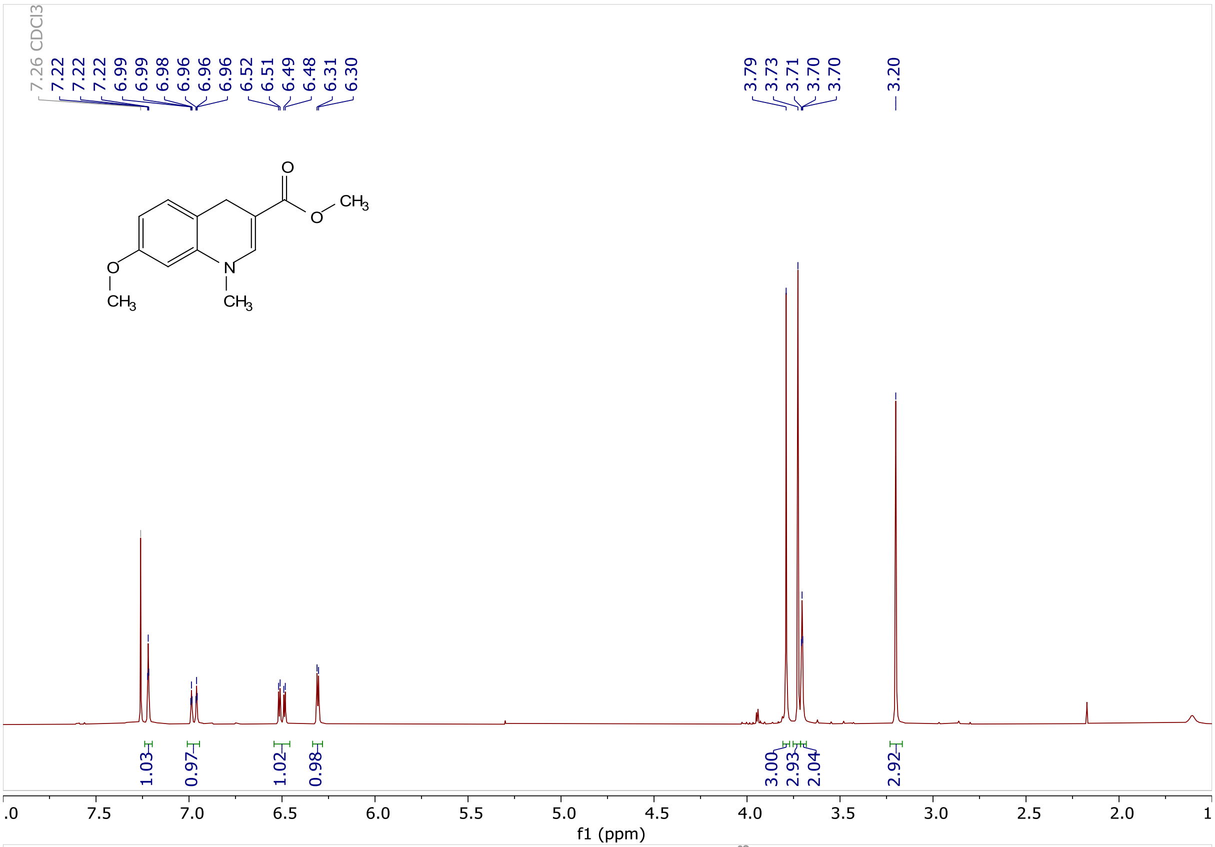

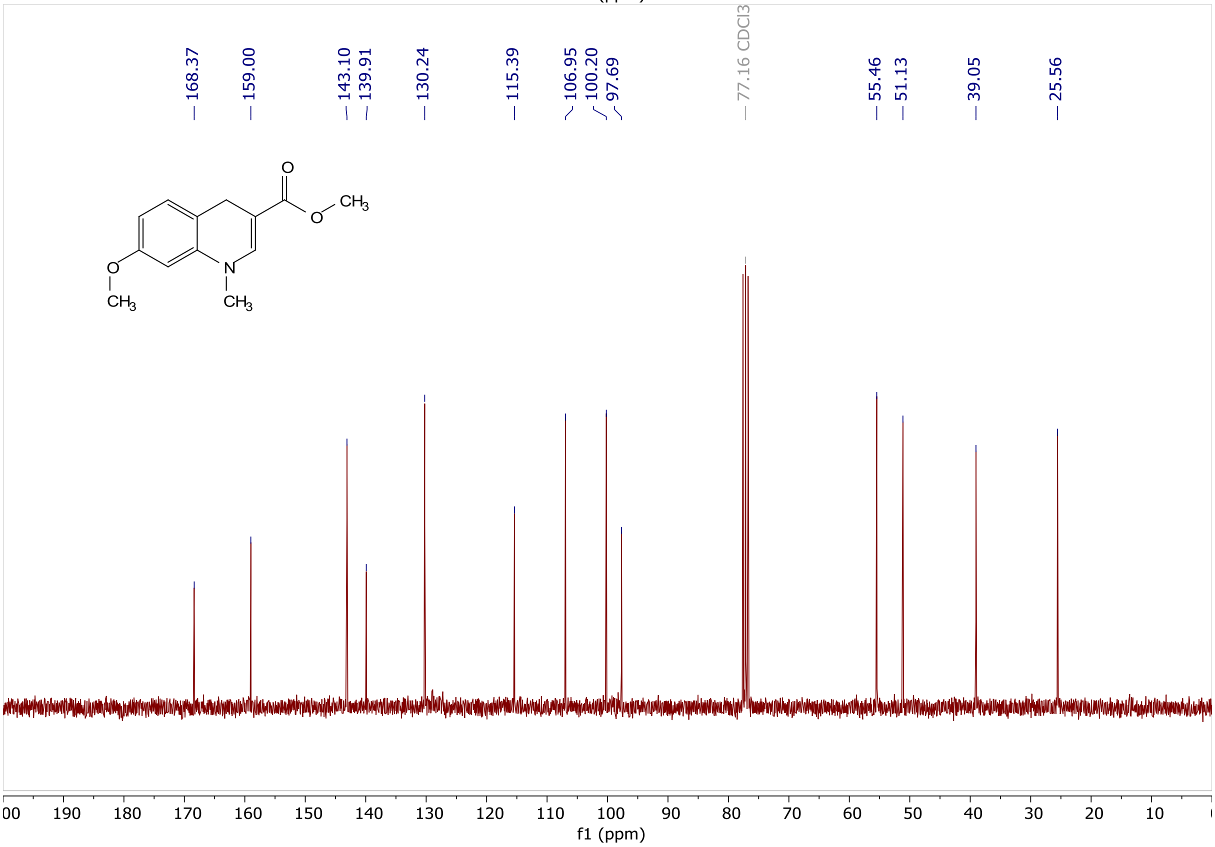

Ethyl 7-methoxy-1-methyl-1,4-dihydroquinoline-3-carboxylate (**1k**)

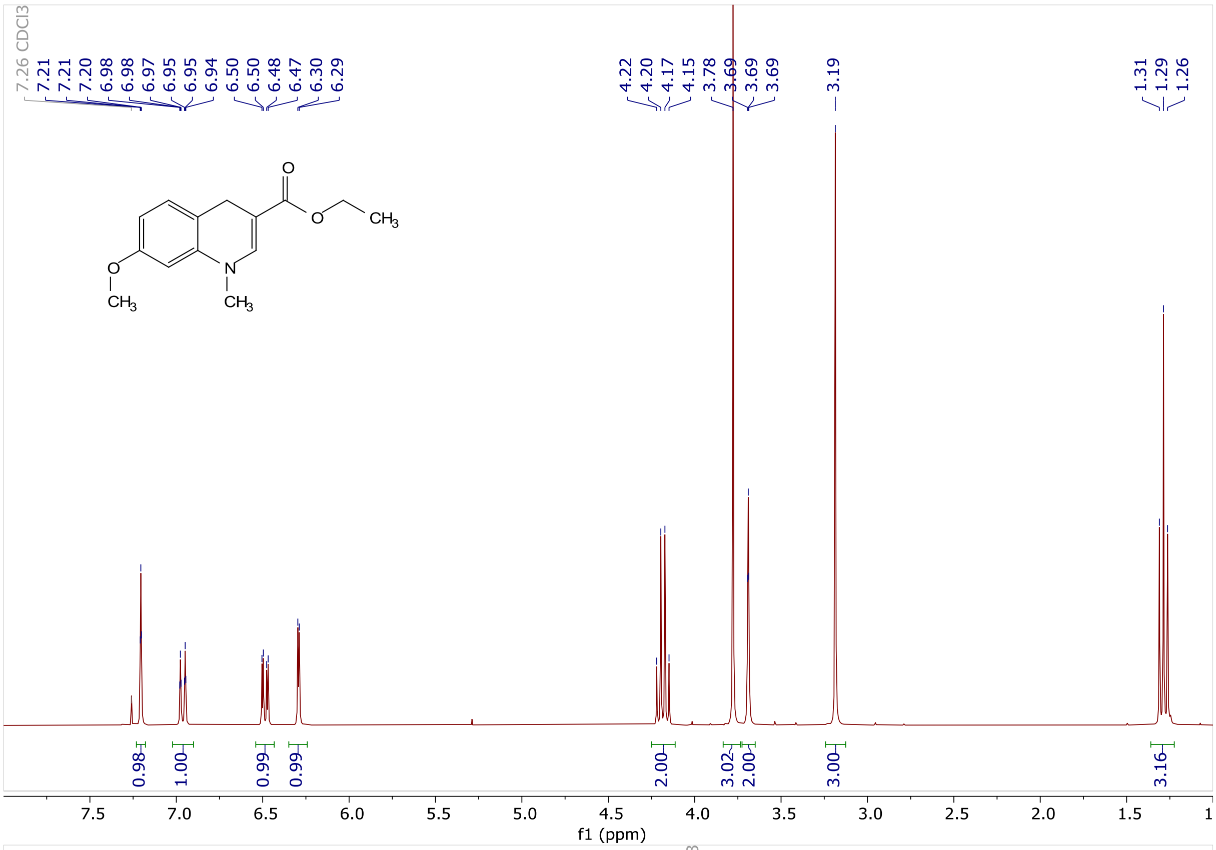

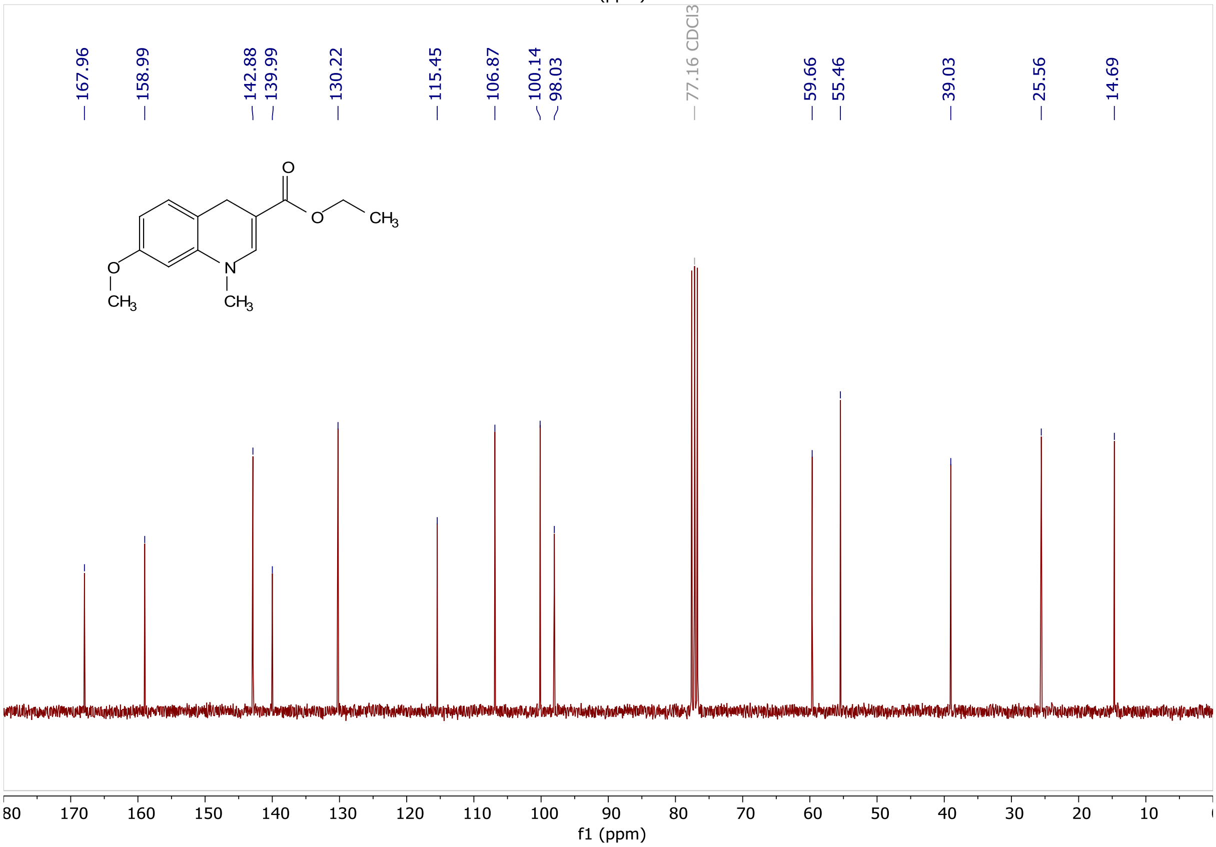

7-Methoxy-N,N,1-trimethyl-1,4-dihydroquinoline-3-carboxamide (**1l**)

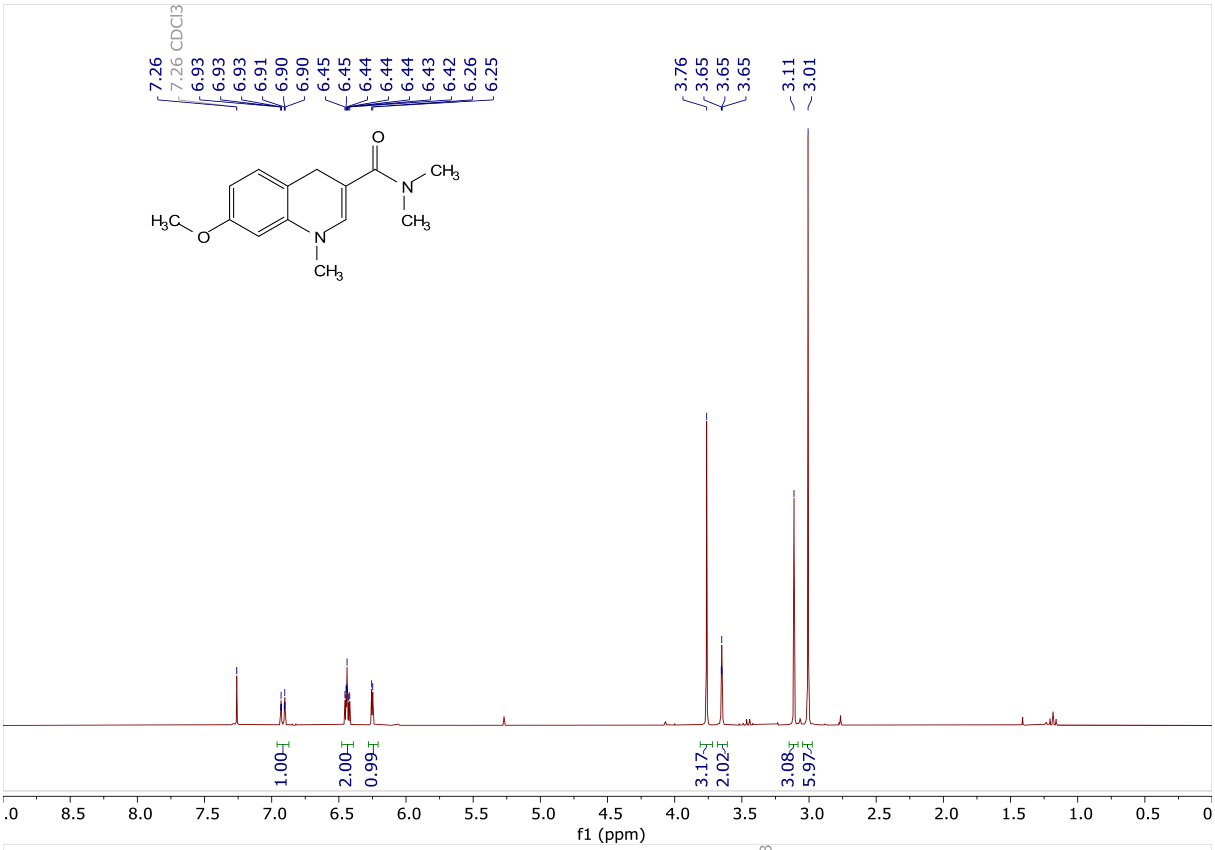

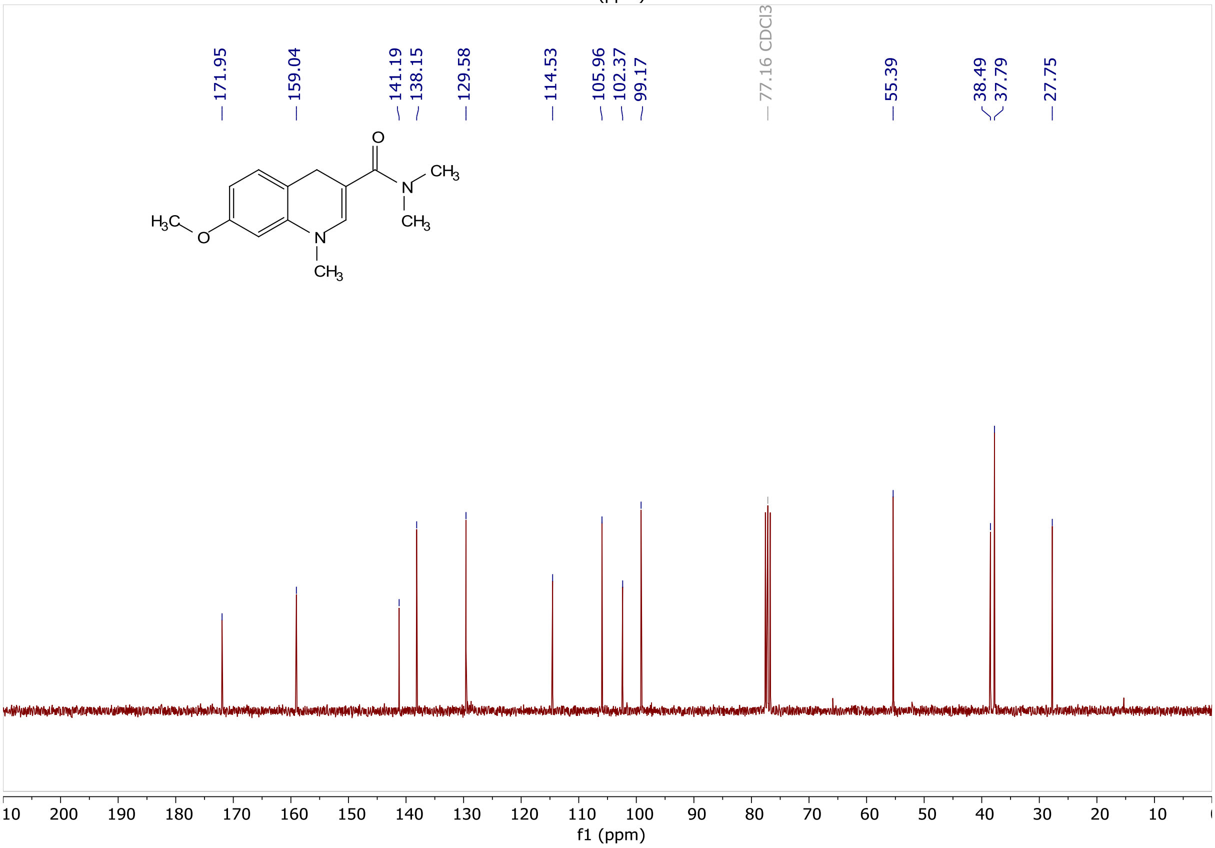

N,7-Dimethoxy-N,1-dimethyl-1,4-dihydroquinoline-3-carboxamide (**1m**)

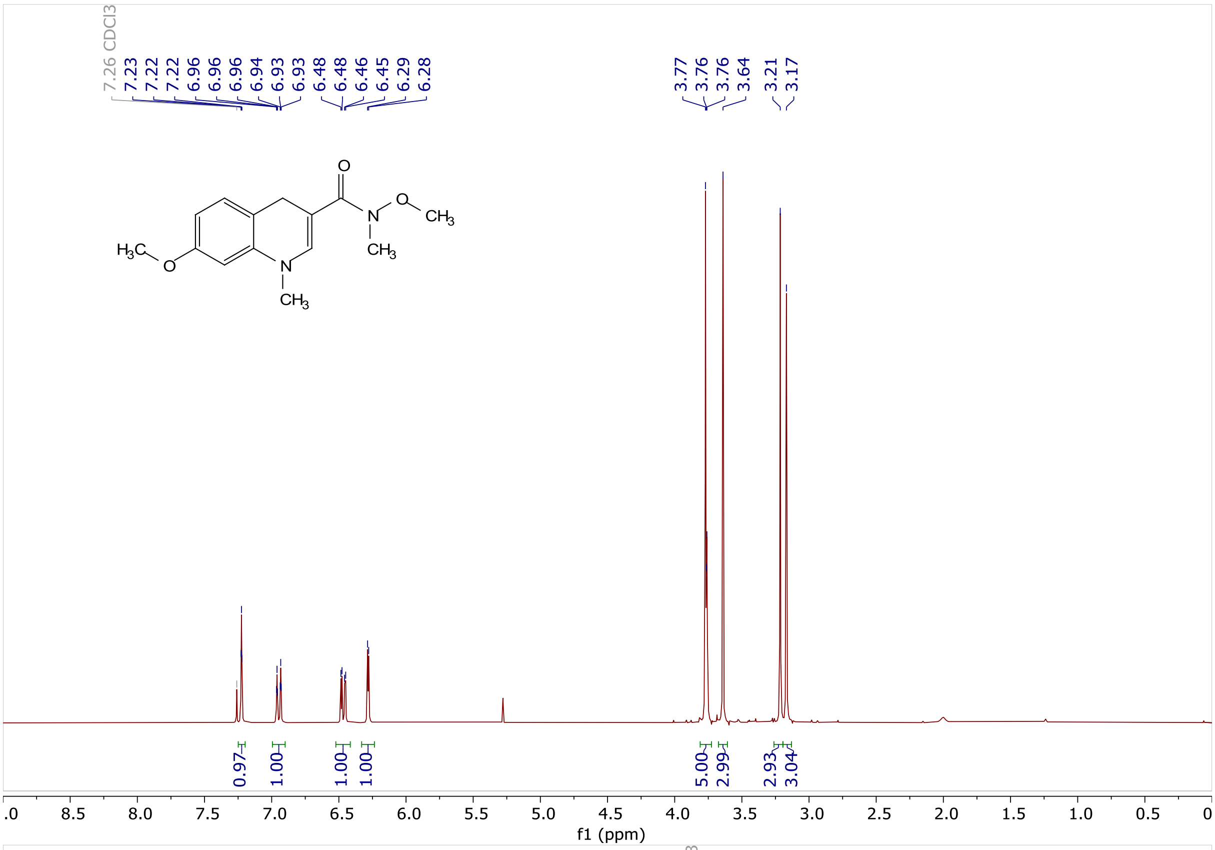

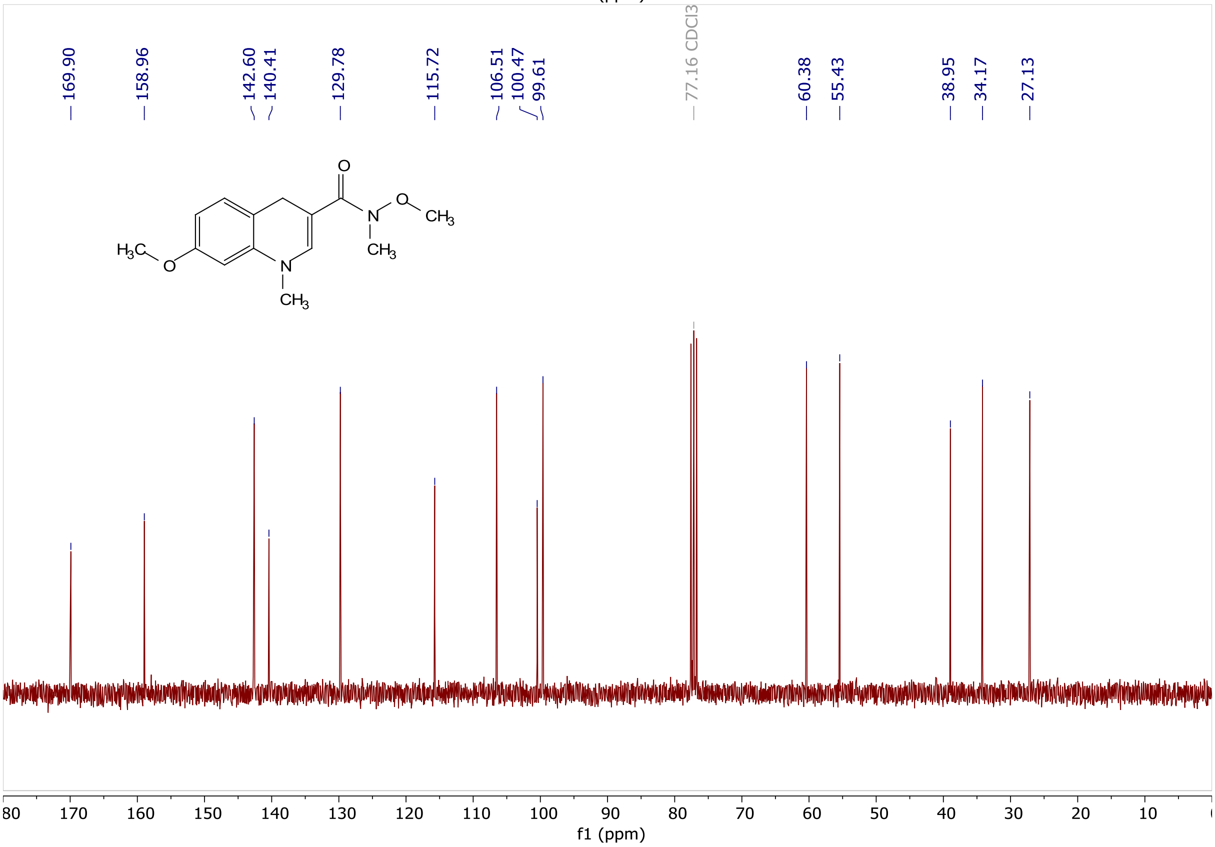

1-(1-Benzyl-7-methoxy-1,4-dihydroquinolin-3-yl)ethanone (**1n**)

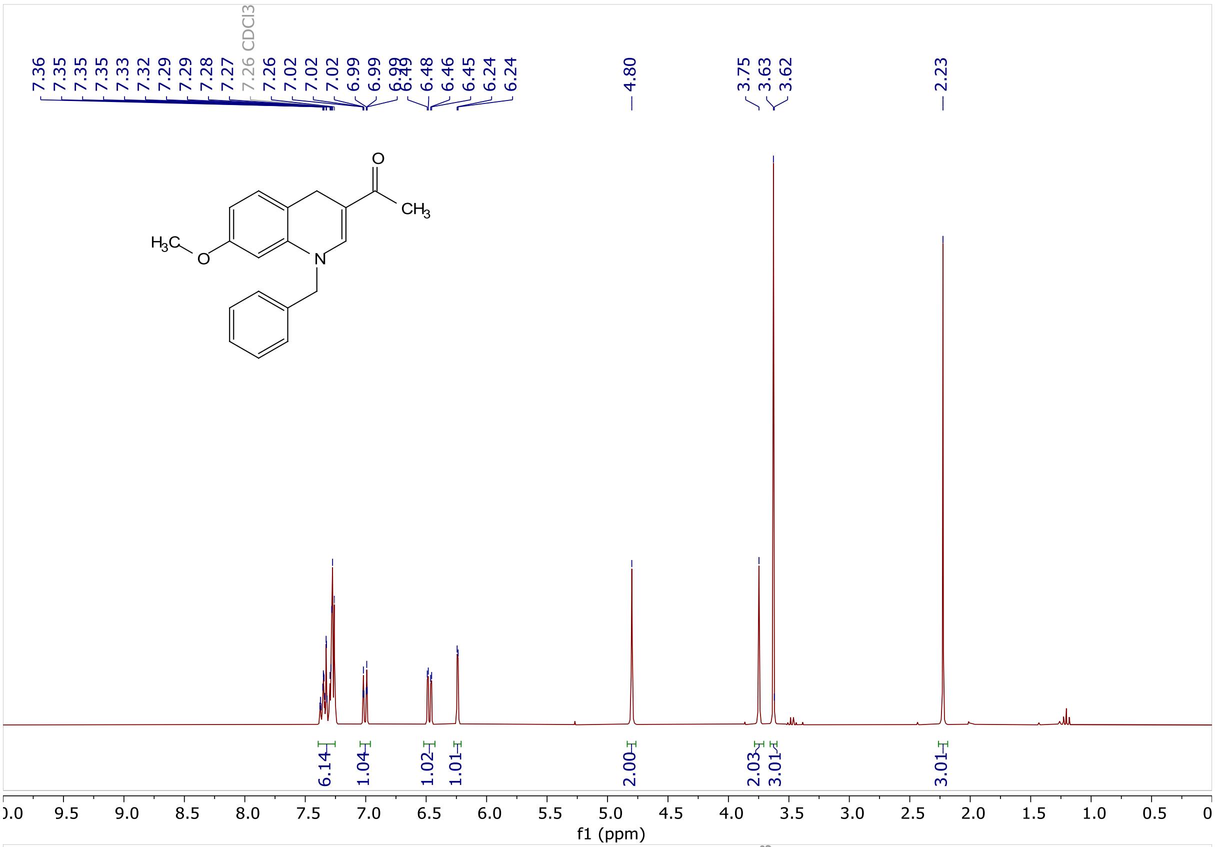

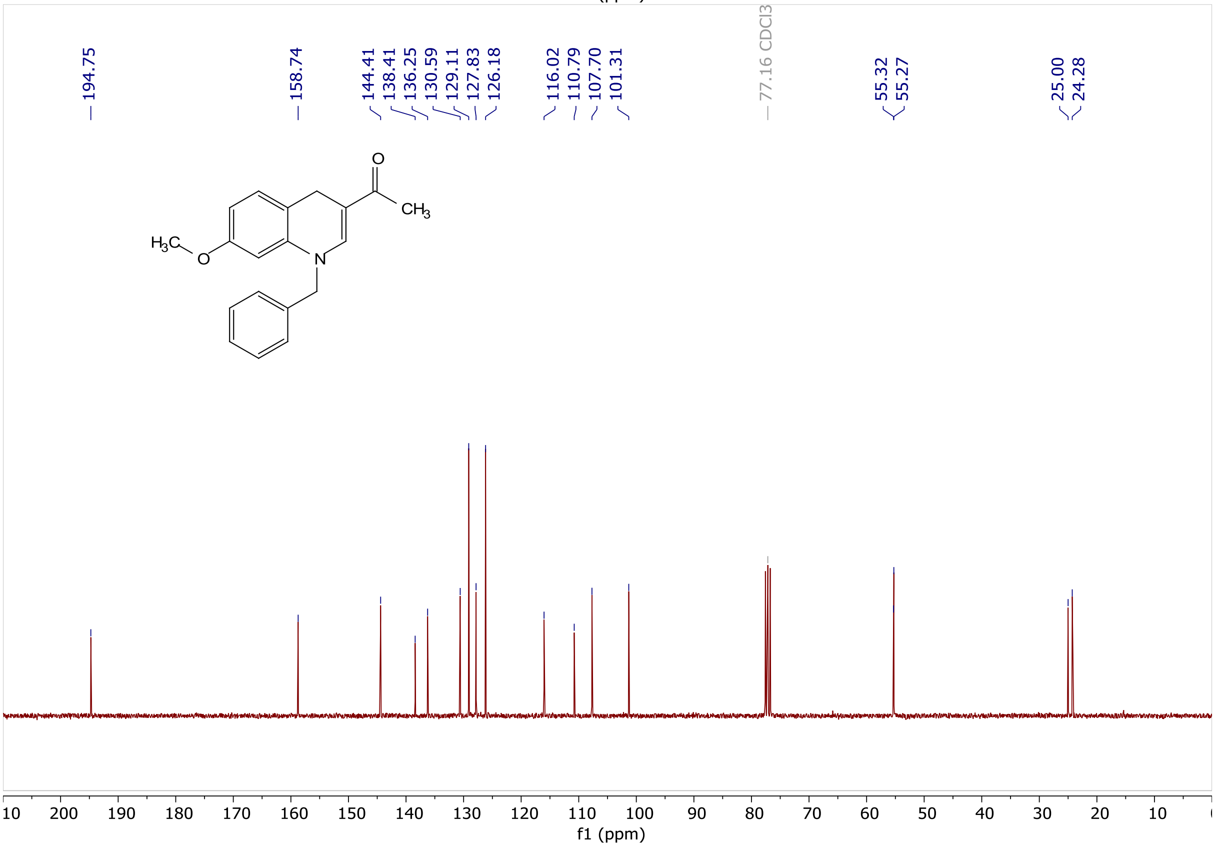

1-(7-Methoxy-1-phenethyl-1,4-dihydroquinolin-3-yl)ethanone (**1o**)

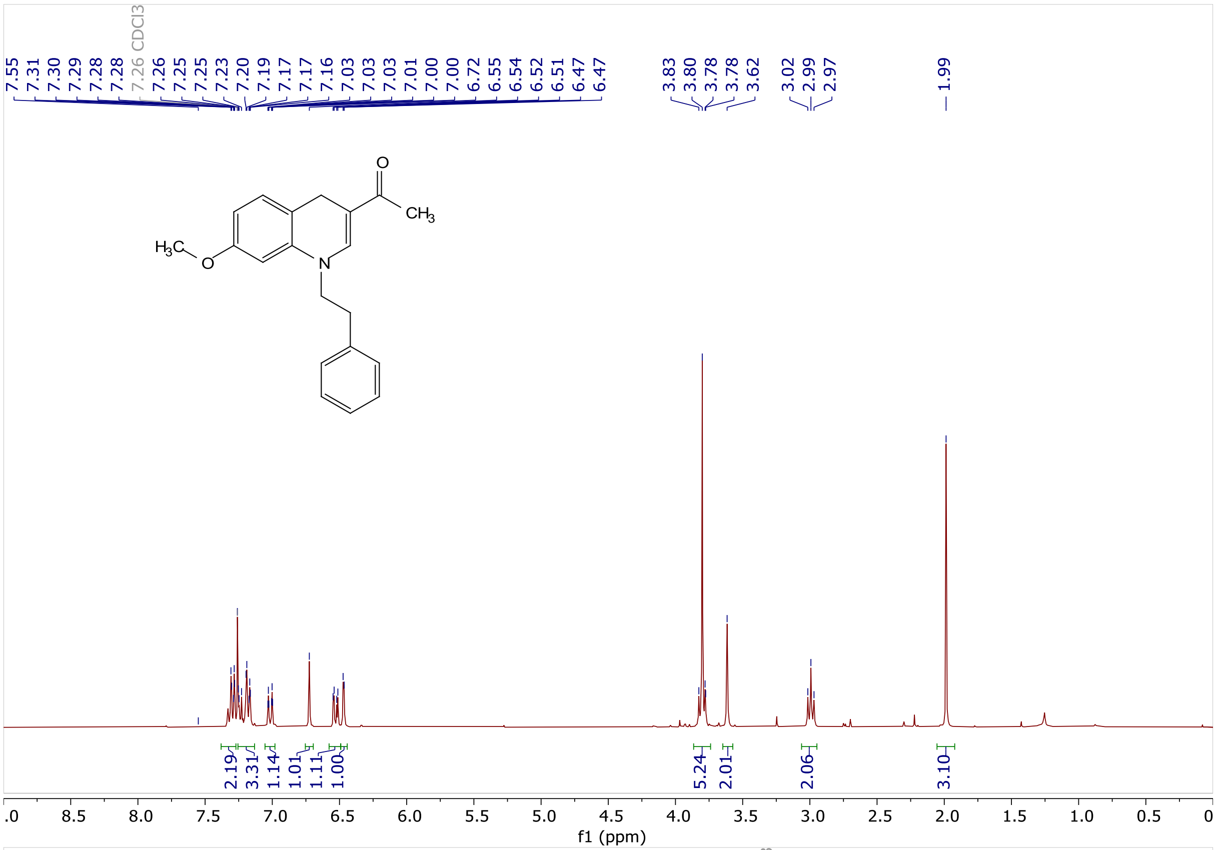

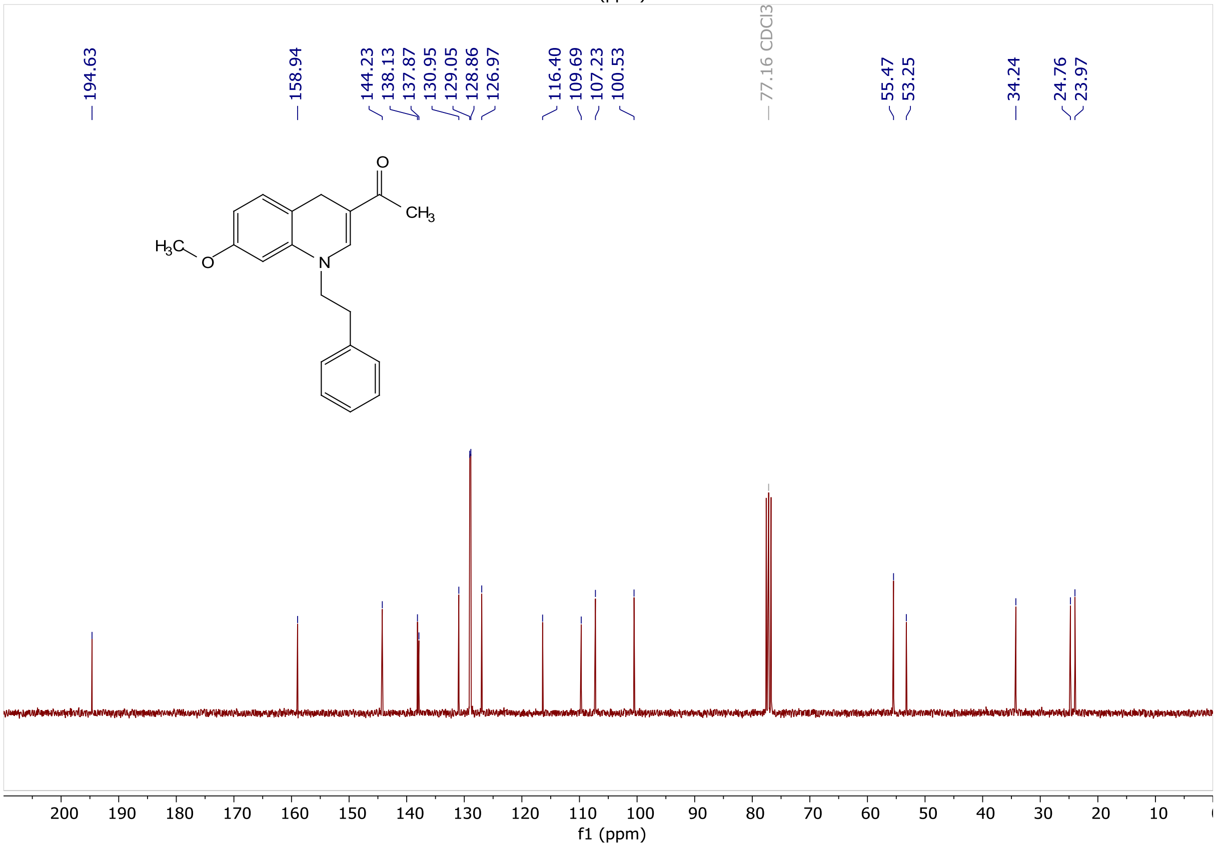

1-(7-Methoxy-1-propyl-1,4-dihydroquinolin-3-yl)ethanone (**1p**)

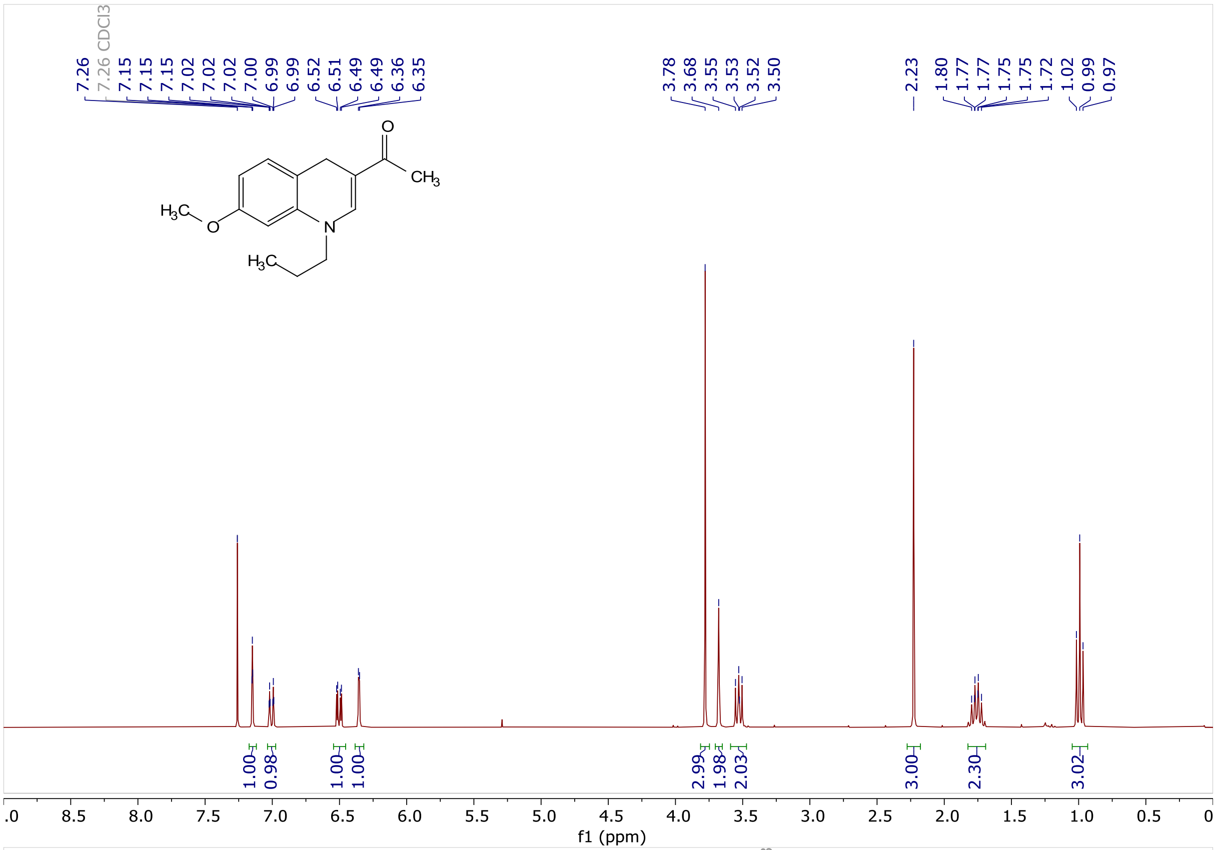

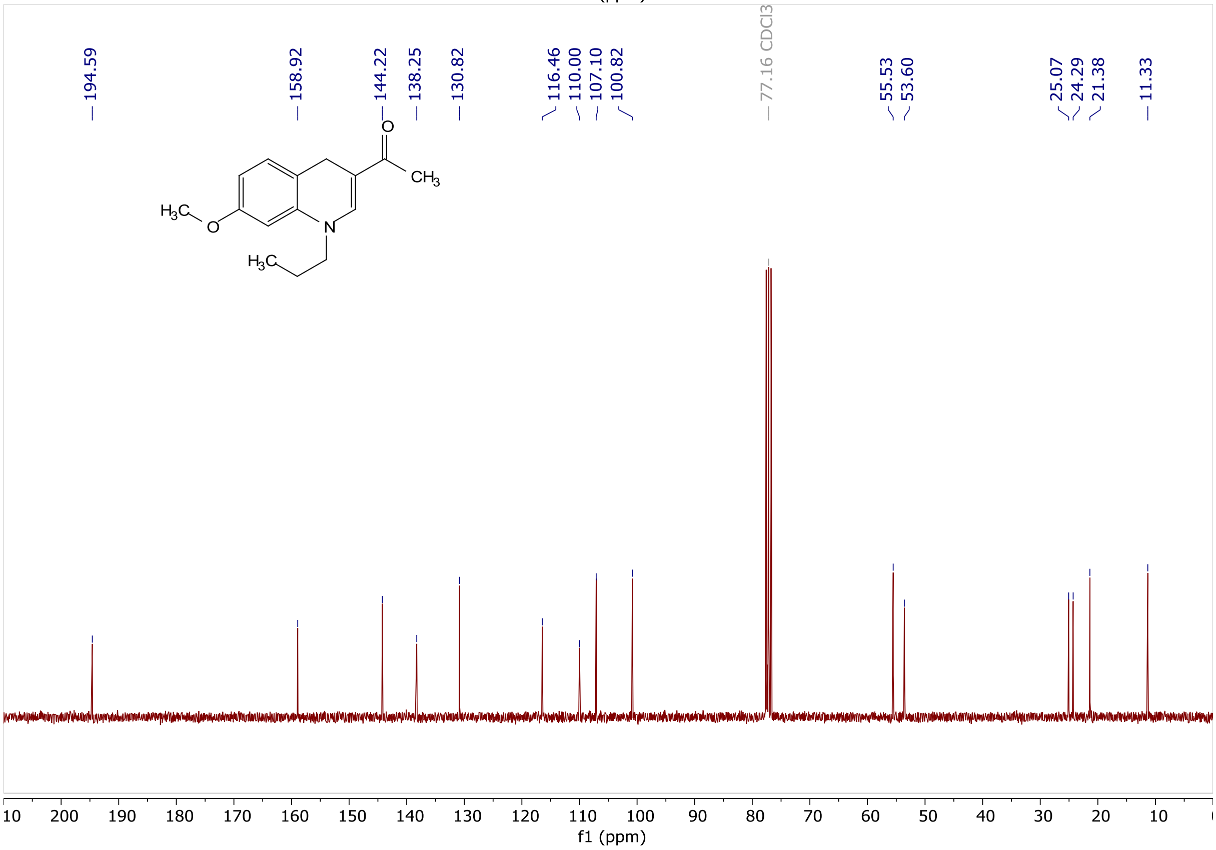

3-Acetyl-1-methylquinolin-1-ium iodide (**2a**)

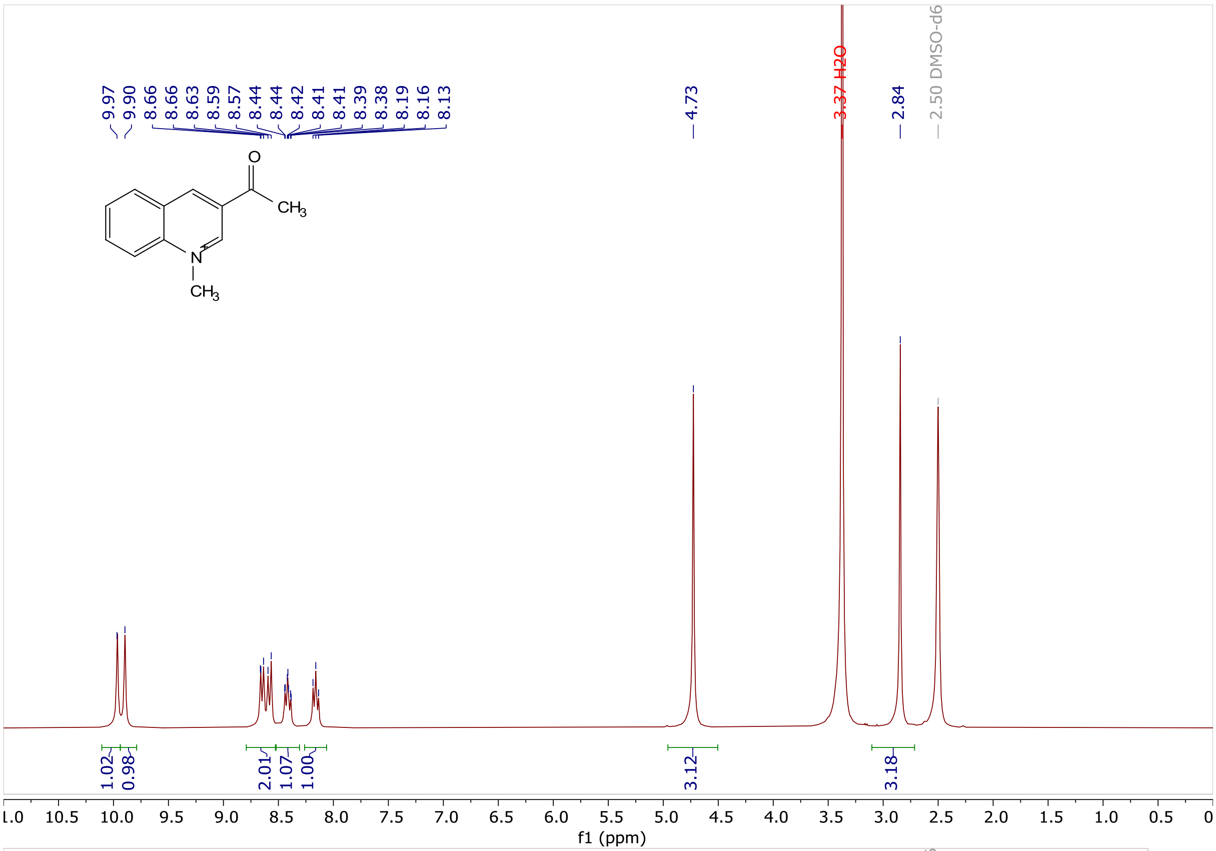

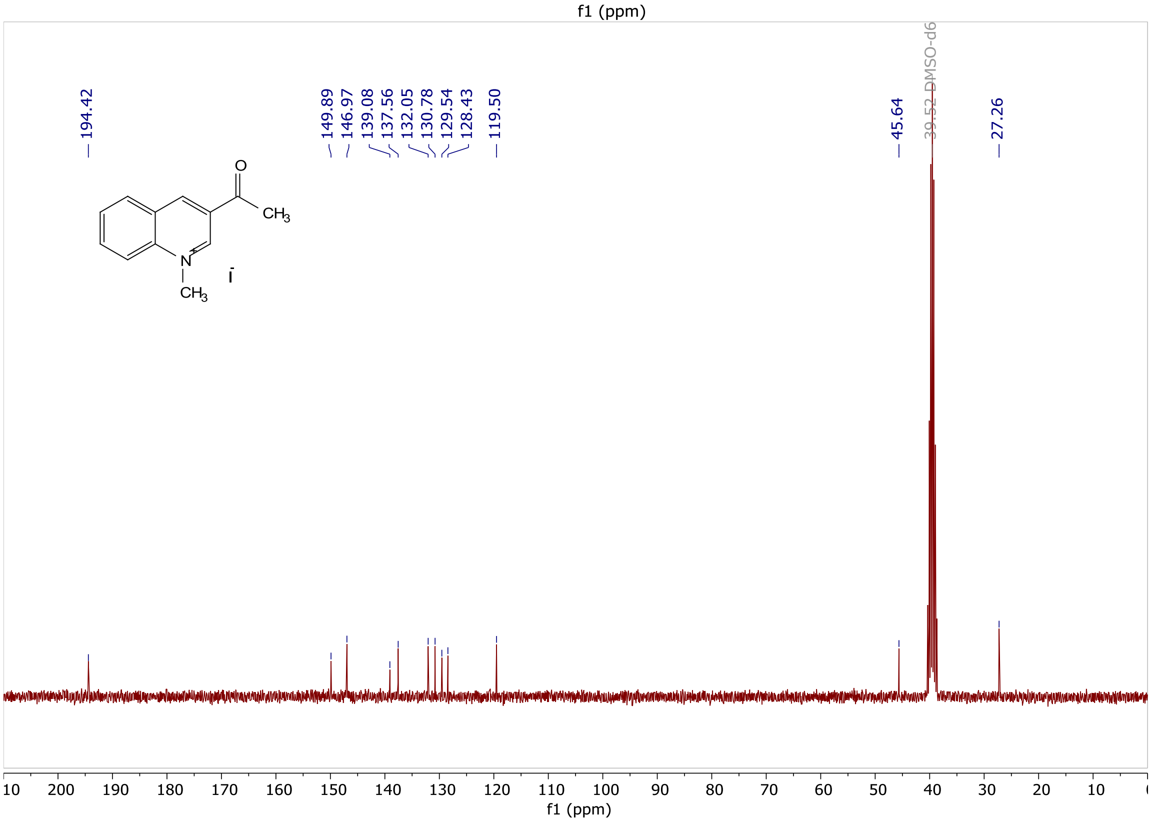

3-Acetyl-1-methyl-7-(trifluoromethyl)quinolin-1-ium iodide (**2b**)

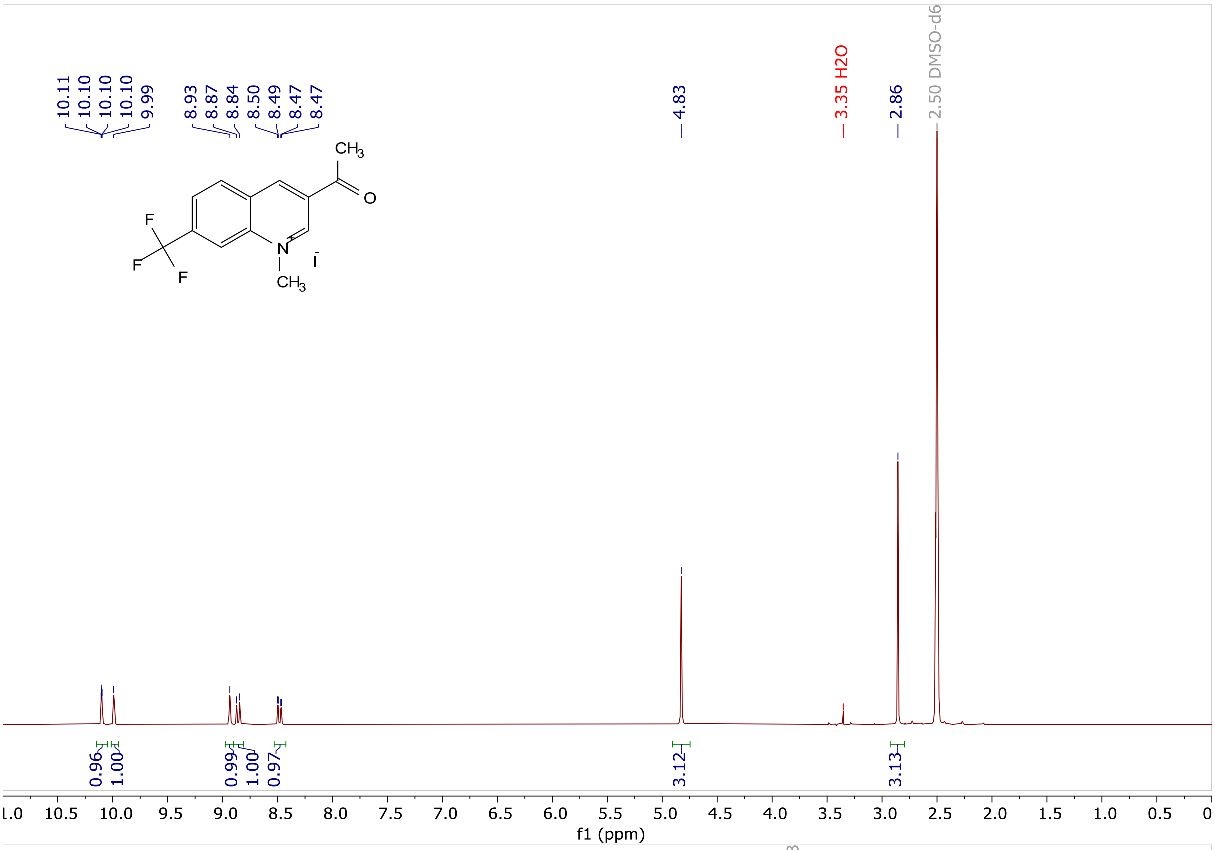

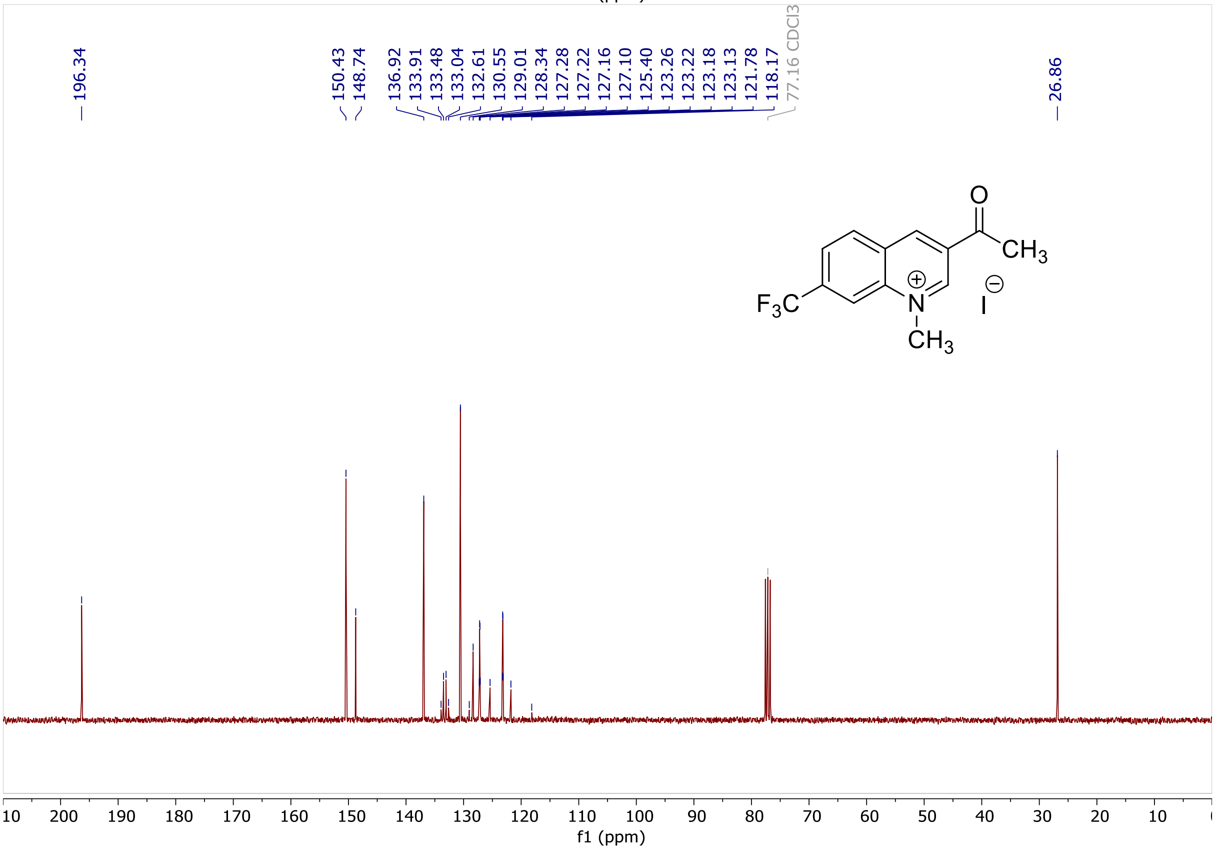

3-Acetyl-7-dimethylamino-1-methylquinolin-1-ium iodide (**2c**)

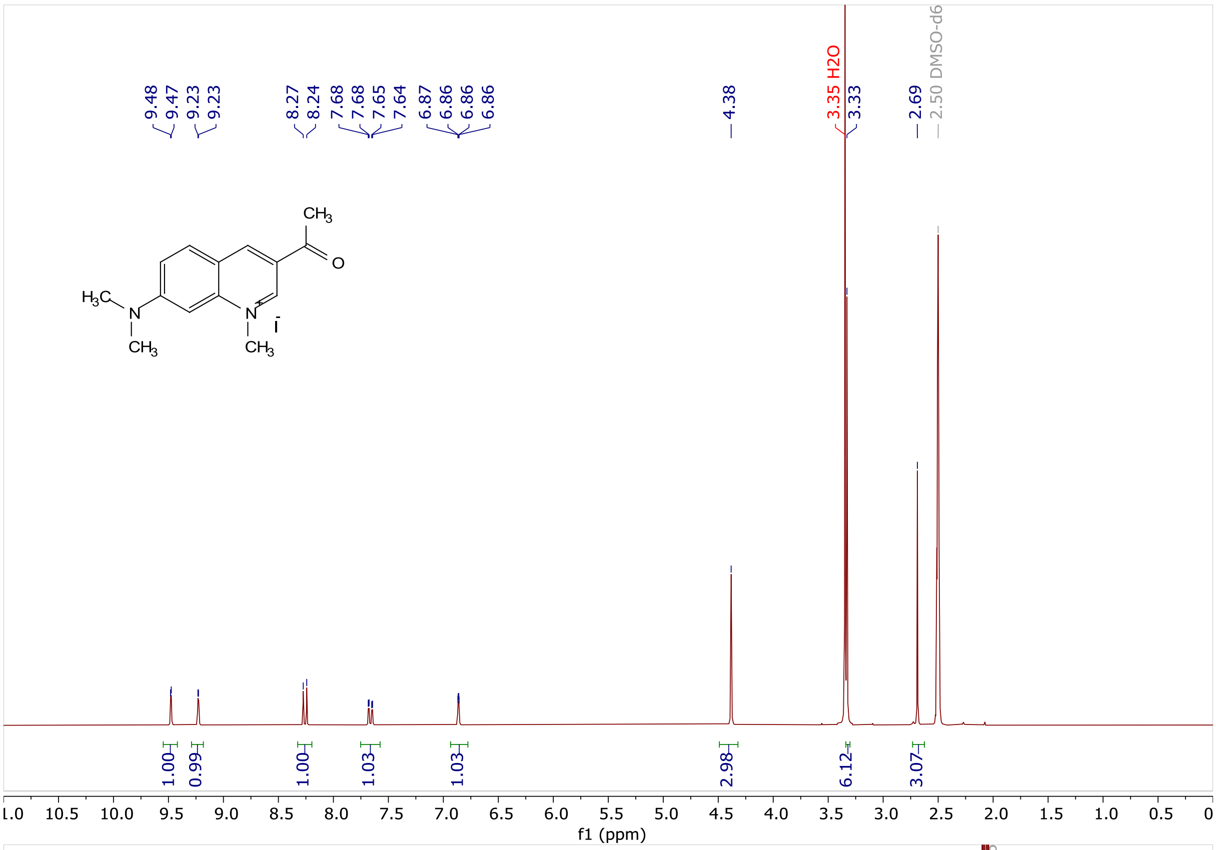

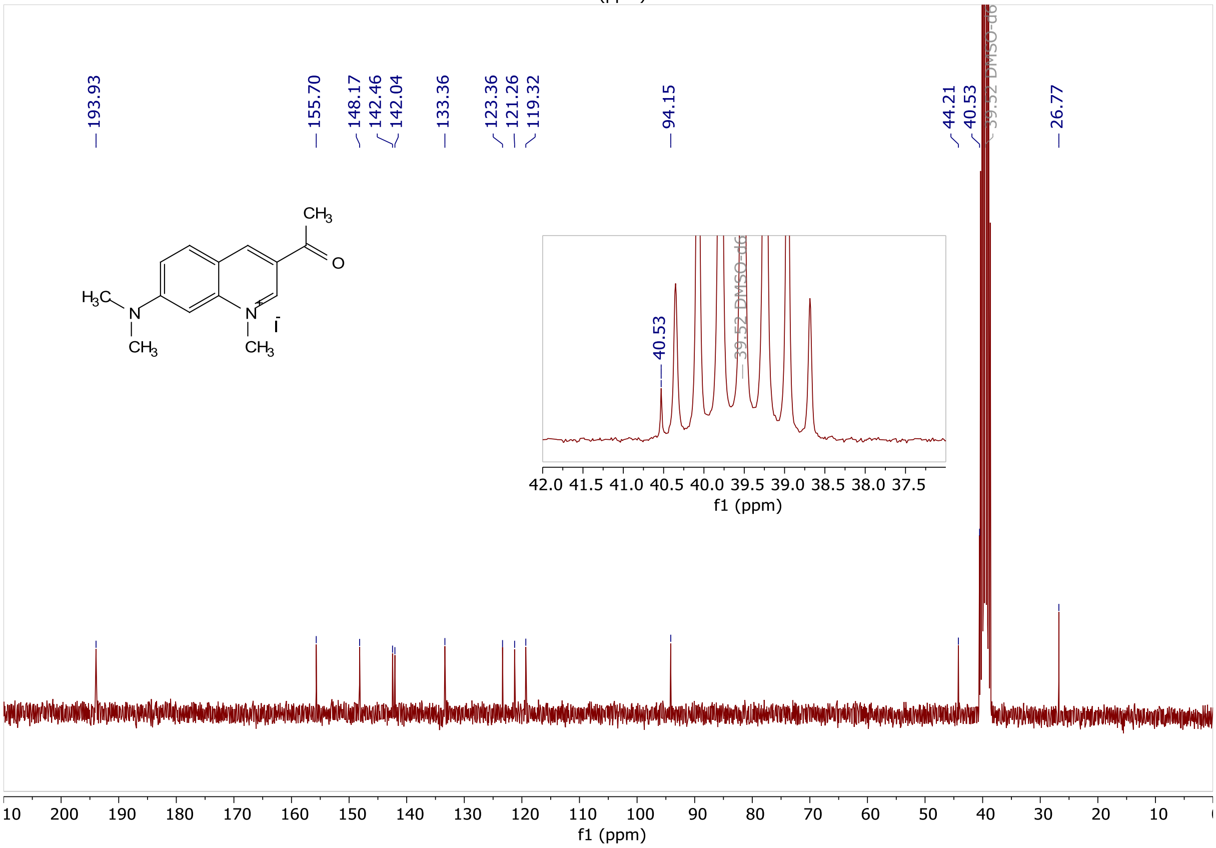

3-Acetyl-7-bromo-1-methylquinolin-1-ium iodide (**2d**)

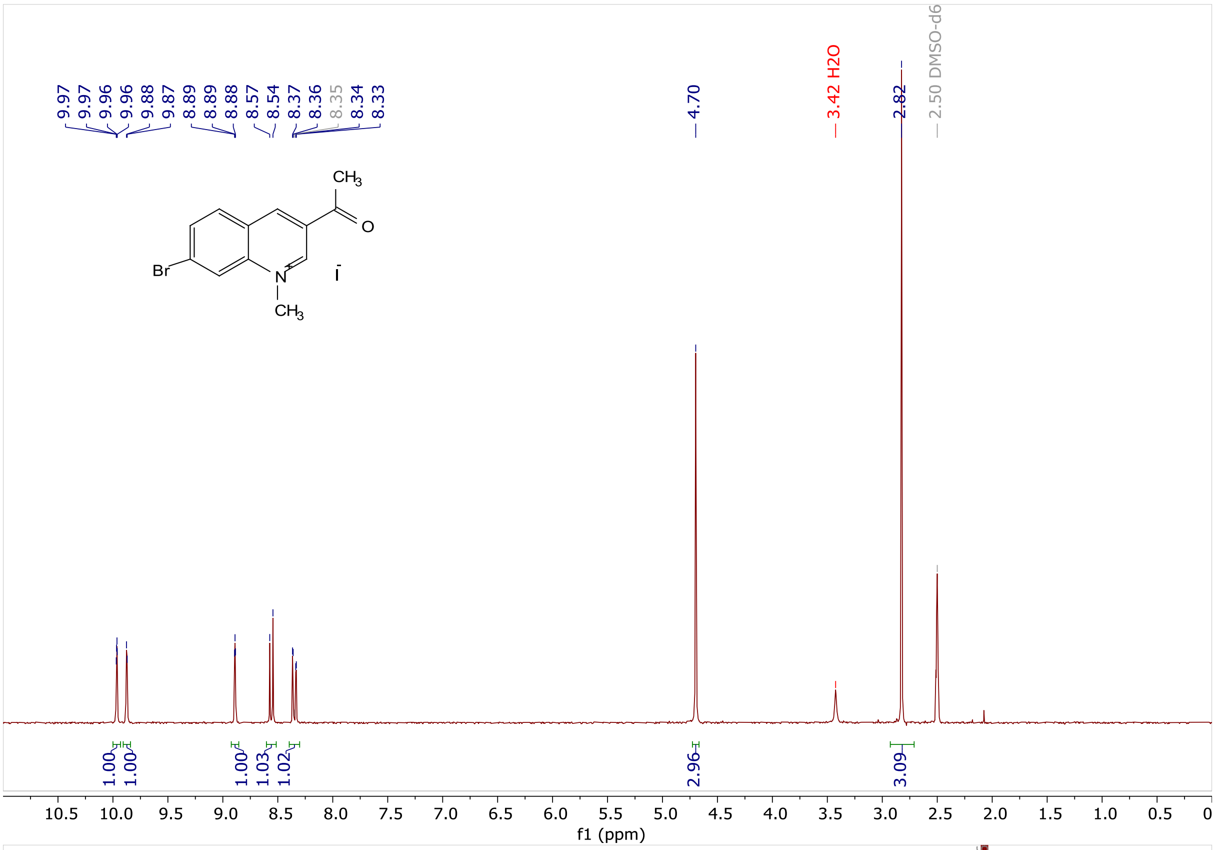

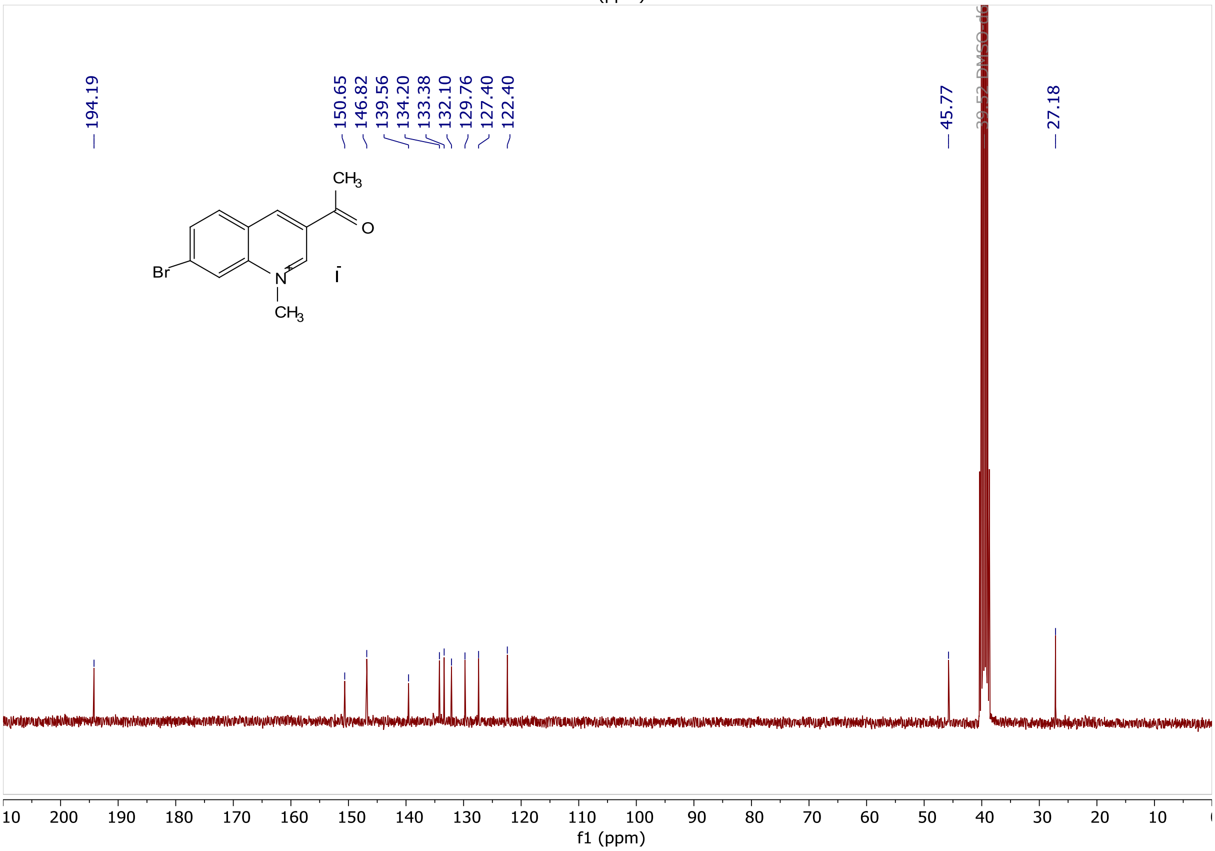

3-Acetyl-6-chloro-1-methylquinolin-1-ium iodide (**2e**)

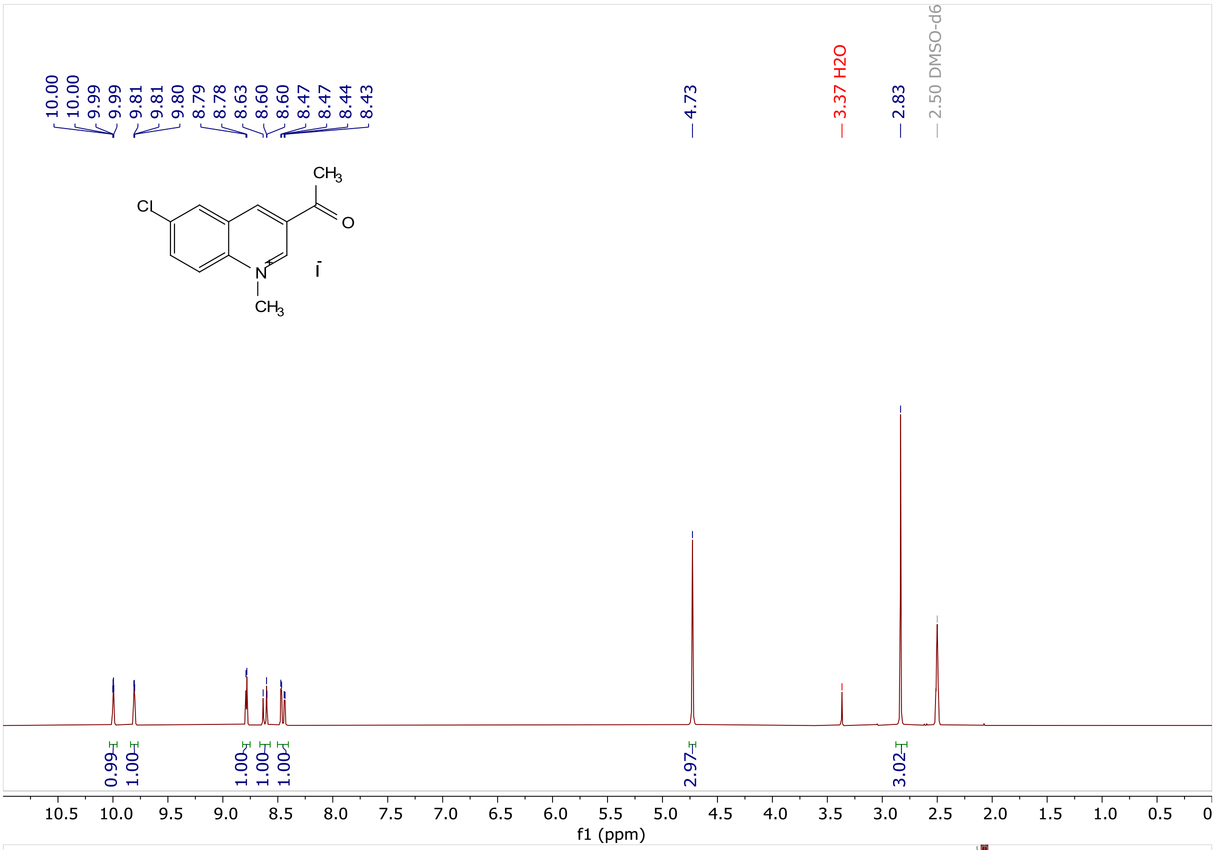

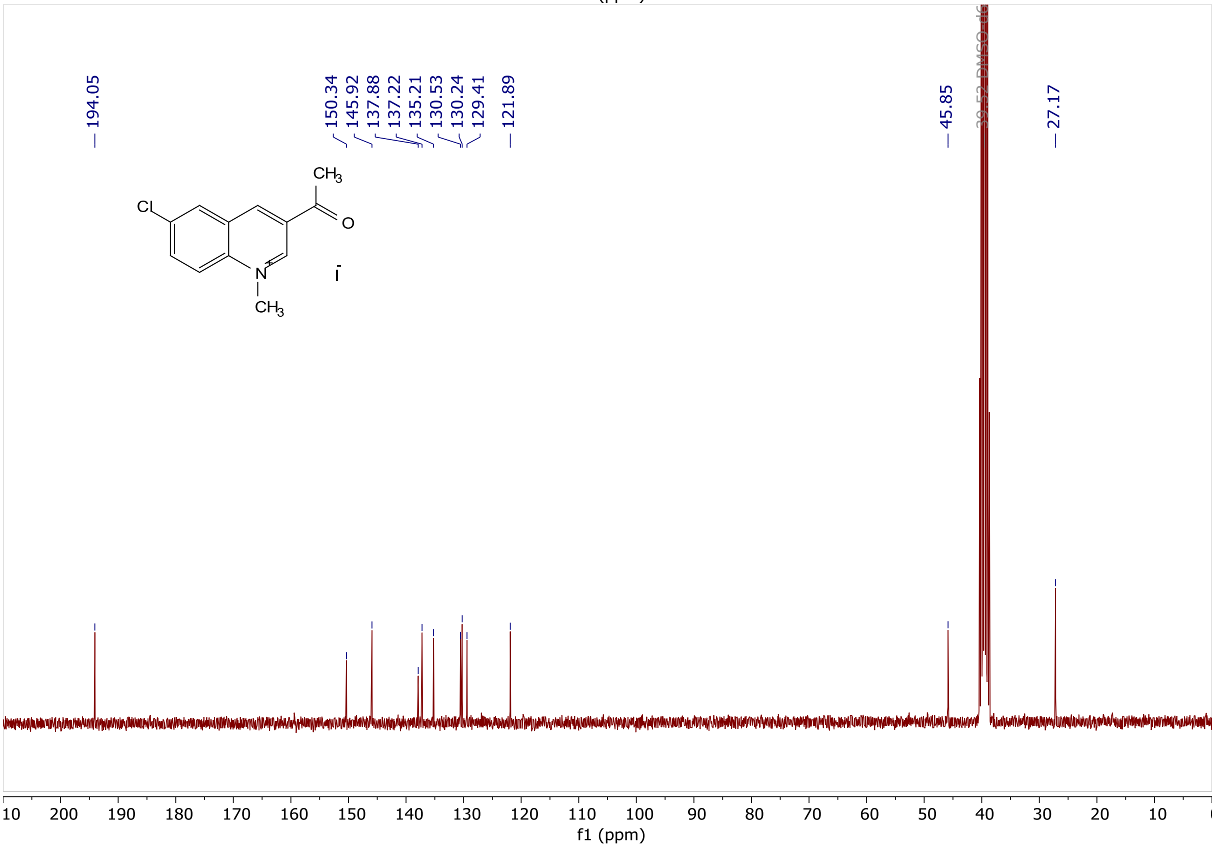

3-Acetyl-6,7-dimethoxy-1-methylquinolin-1-ium iodide (**2f**)

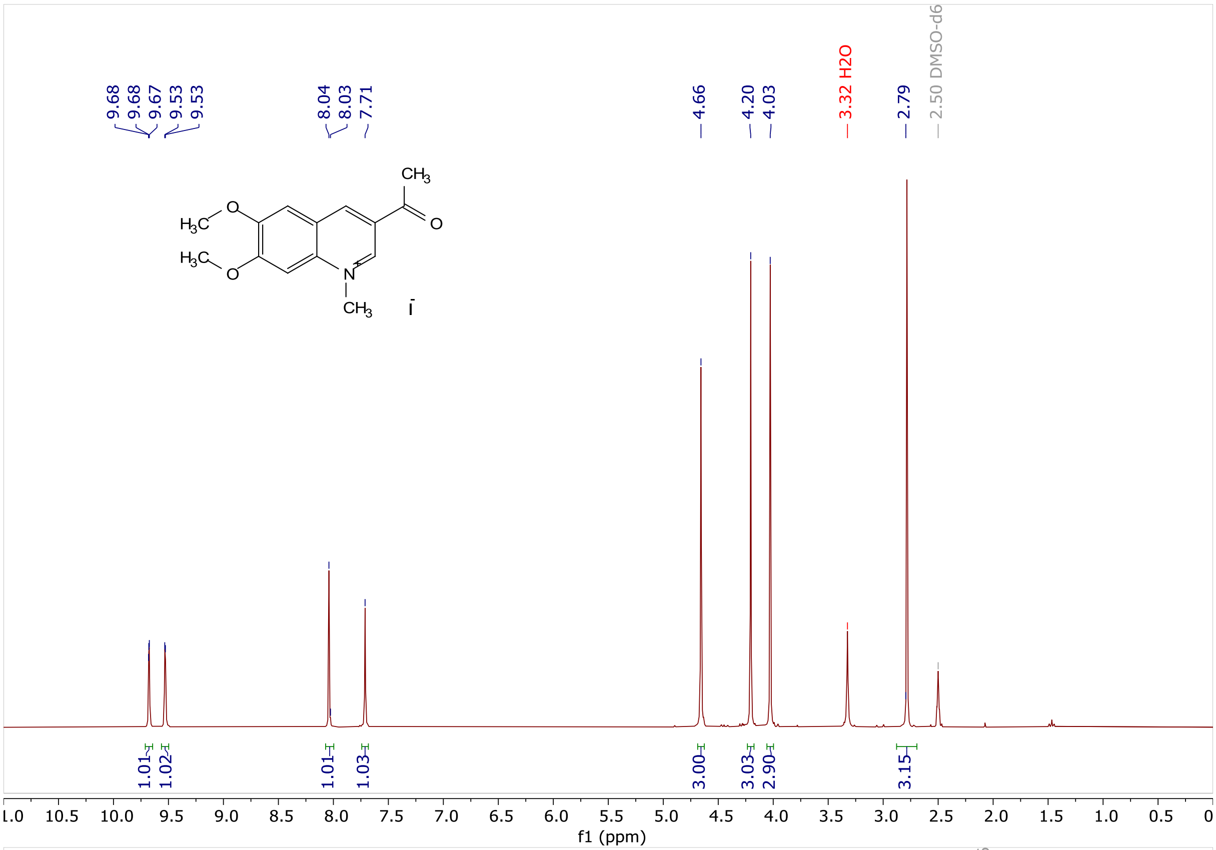

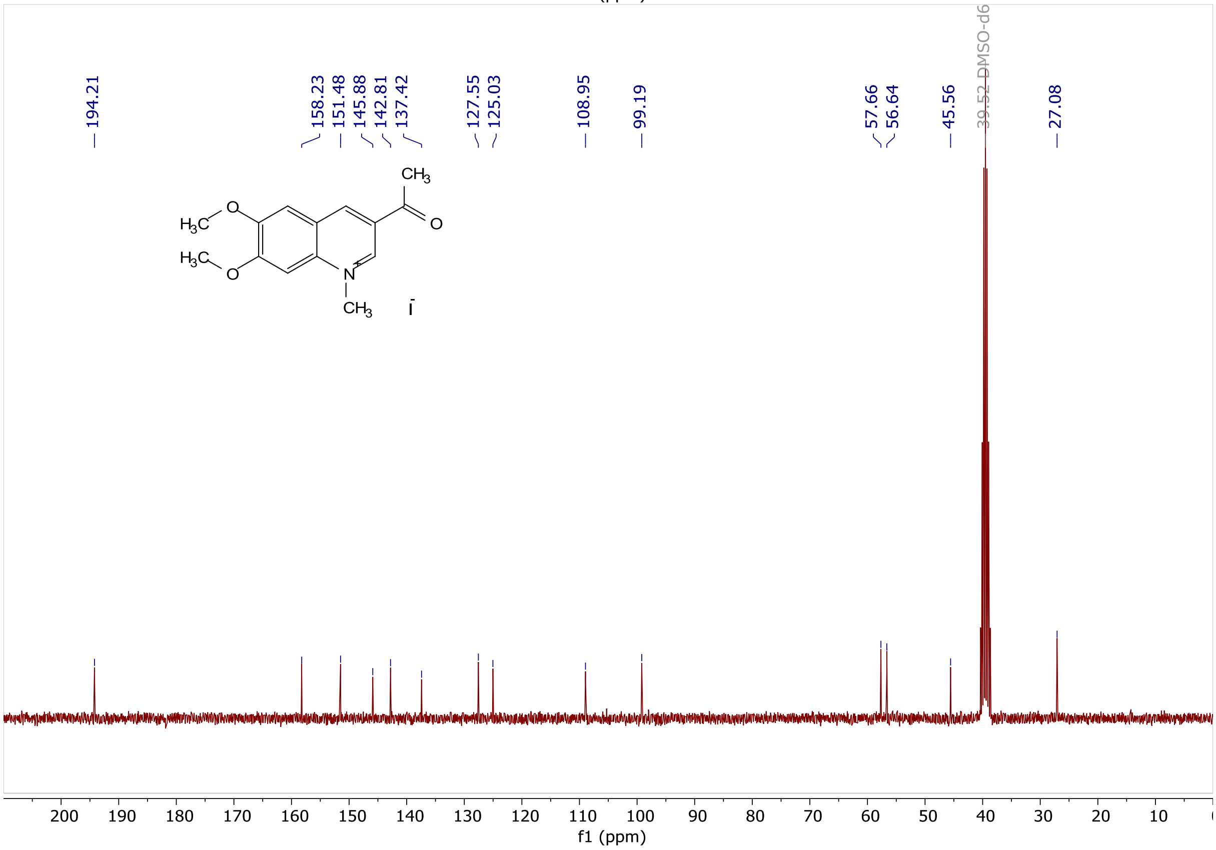

7-Acetyl-5-methyl-[1,3]dioxolo[4,5-g]quinolin-5-ium iodide (**2g**)

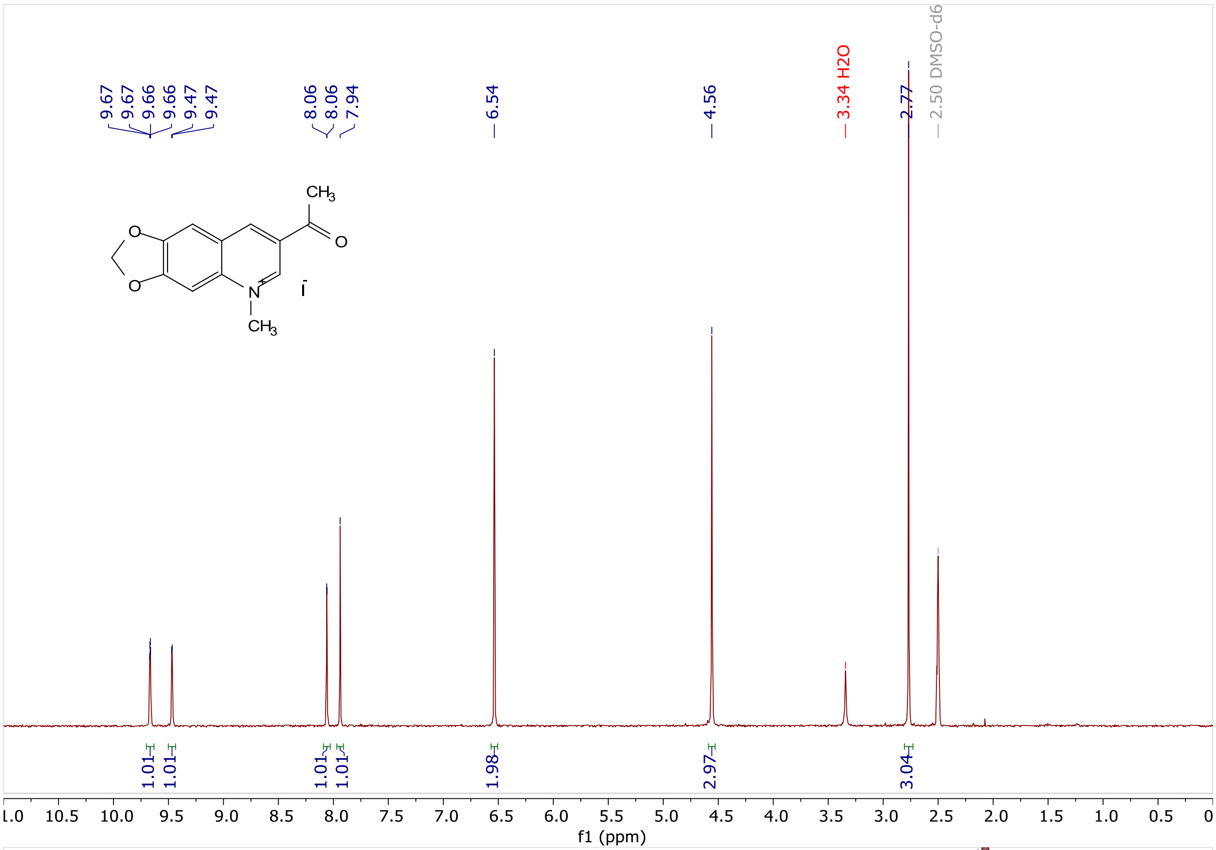

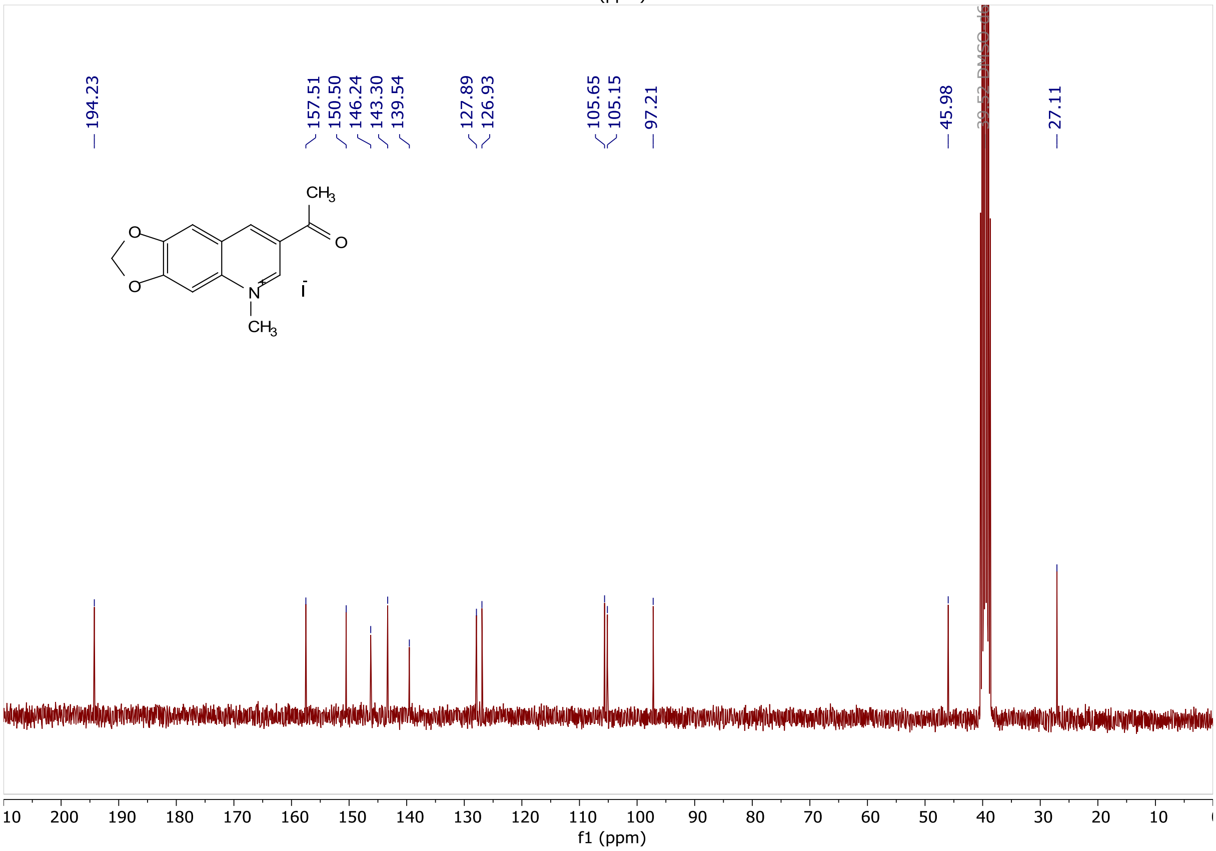

3-Acetyl-7-methoxy-1-methylquinolin-1-ium iodide (**2h**)

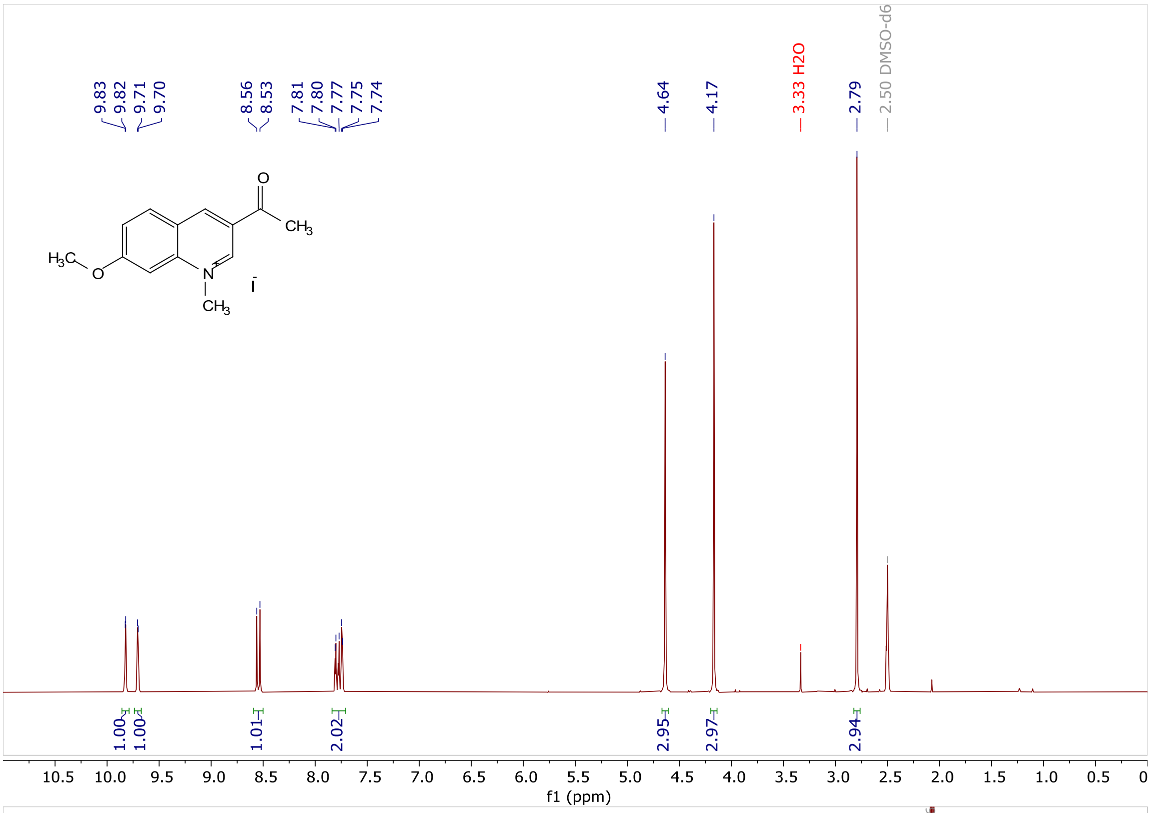

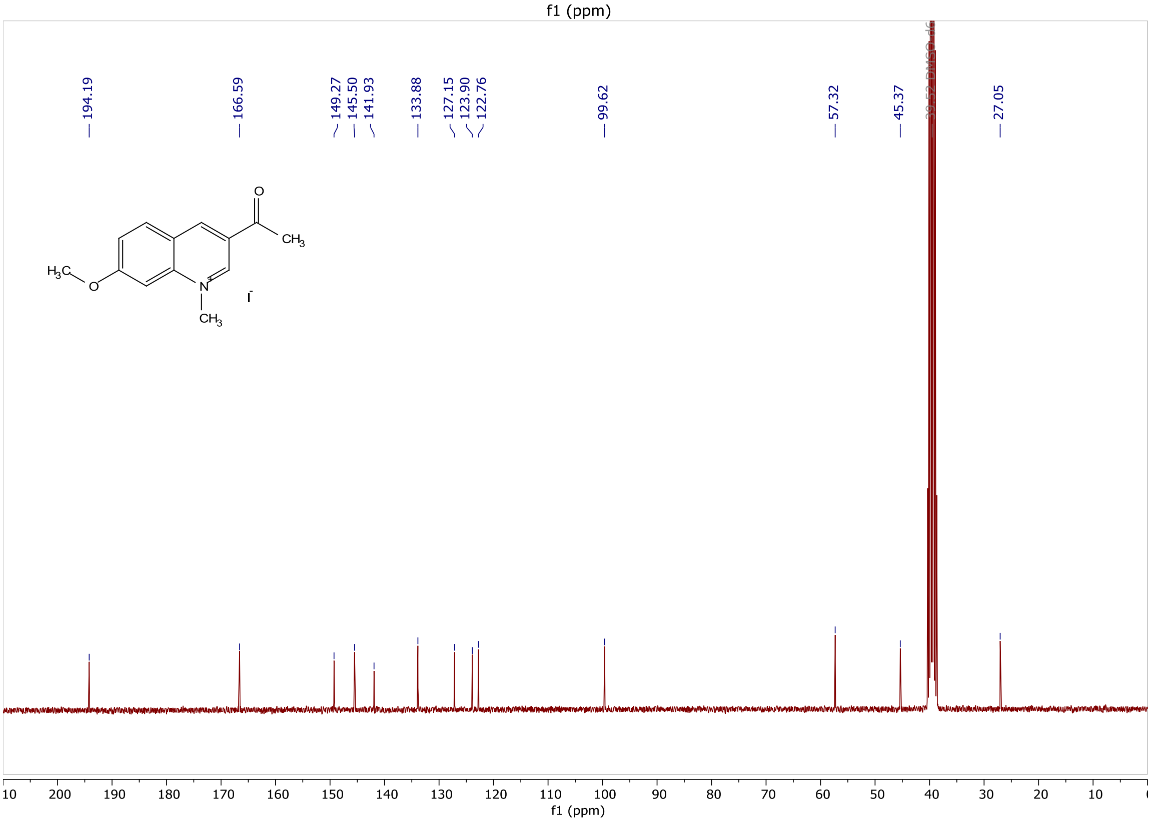

3-Cyano-7-methoxy-1-methylquinolin-1-ium (**2i**)

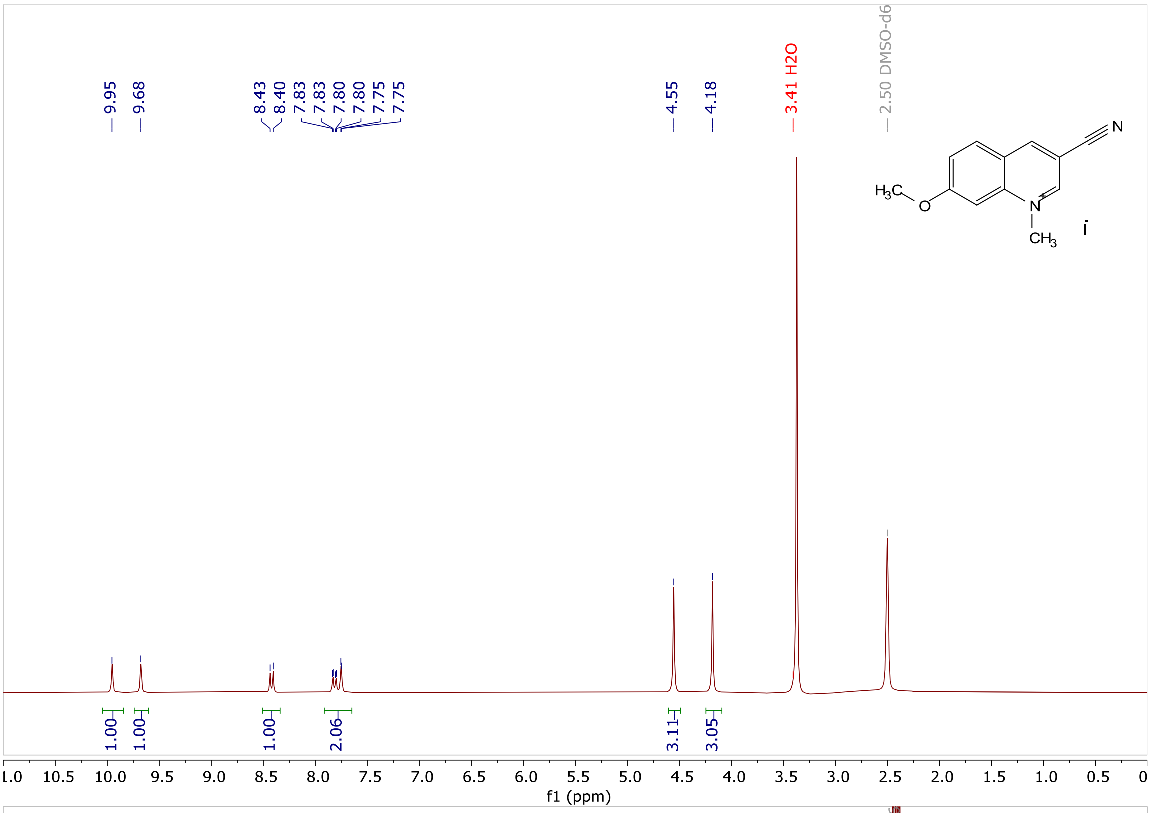

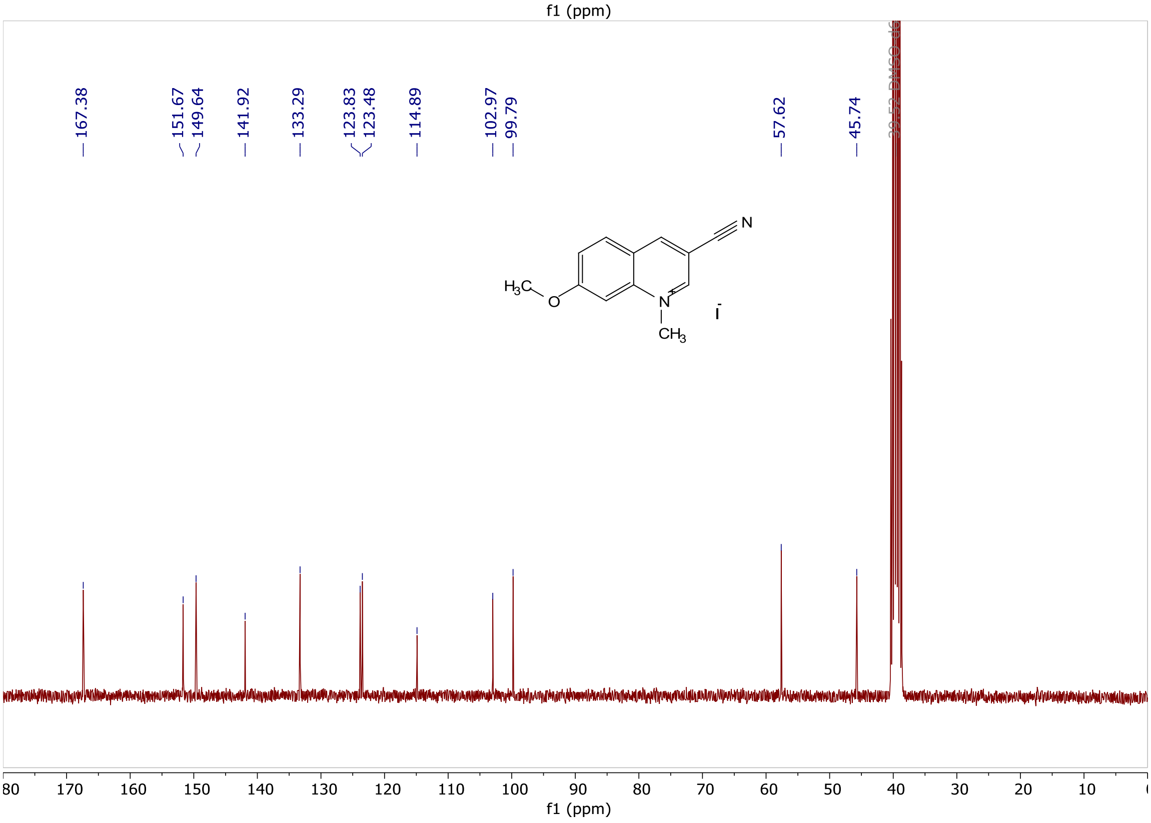

7-Methoxy-3-(methoxycarbonyl)-1-methylquinolin-1-ium (**2j**)

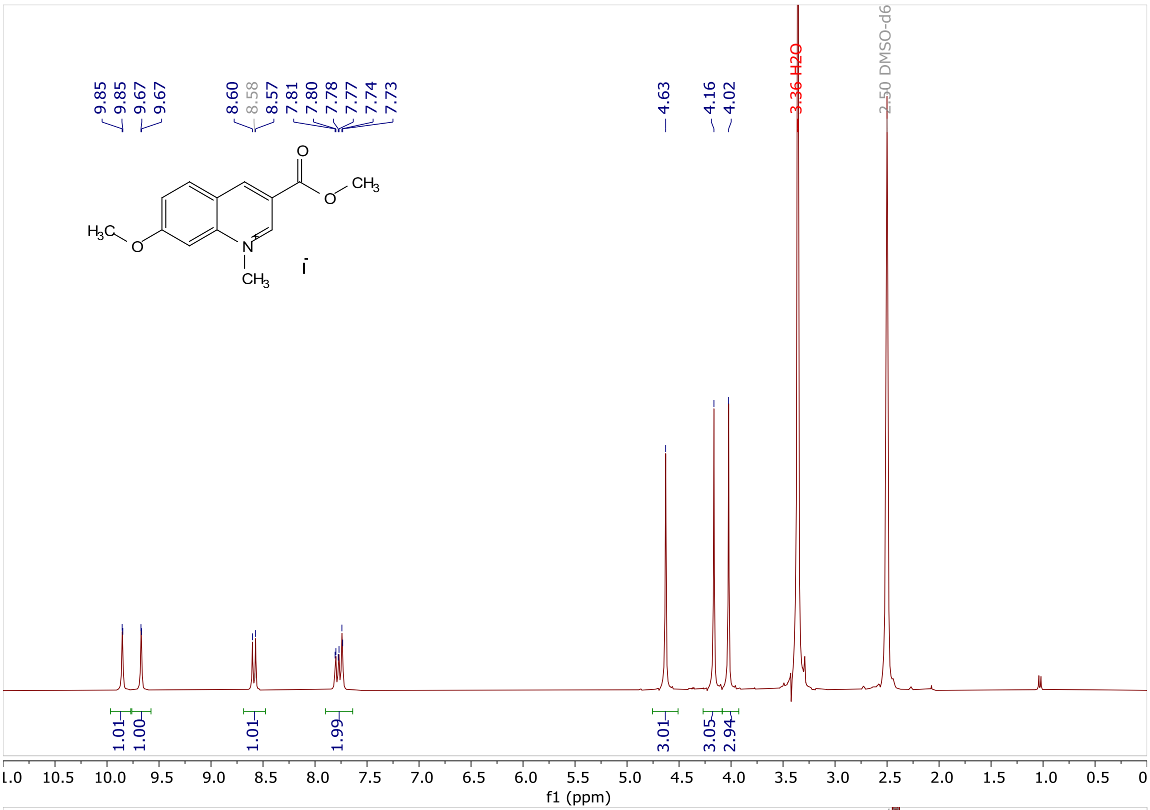

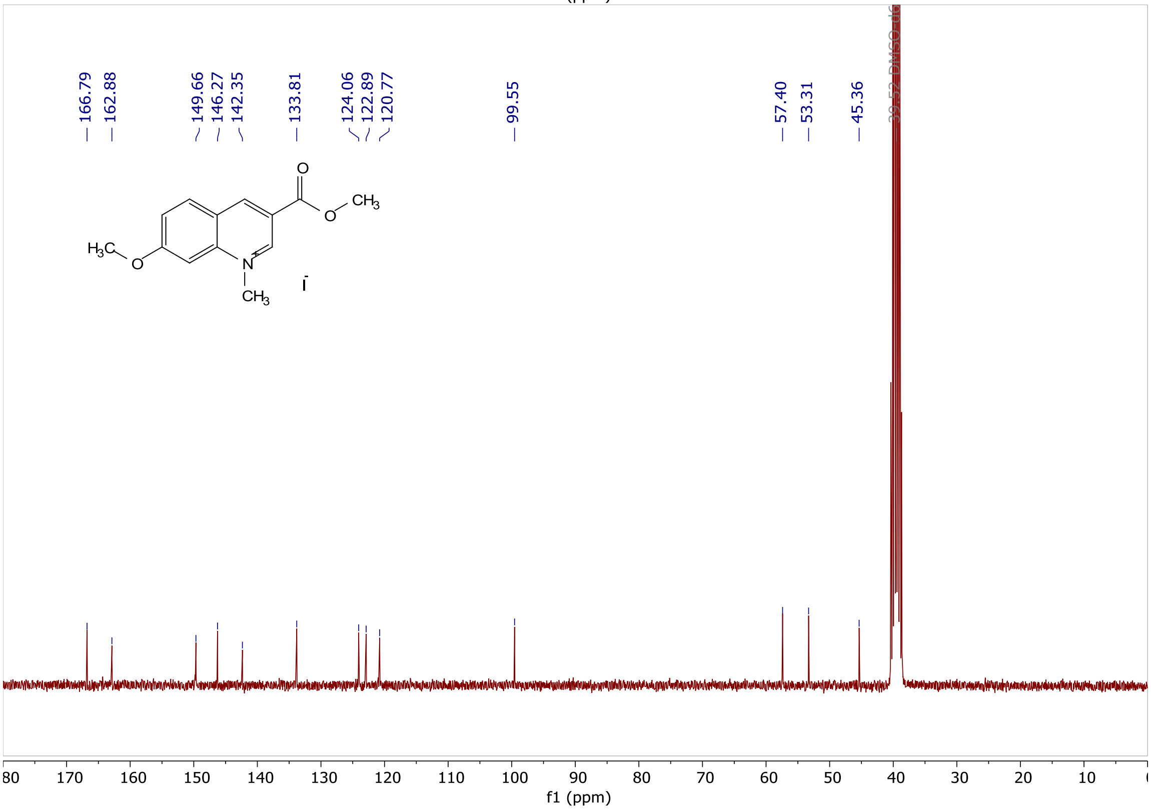

3-(Ethoxycarbonyl)-7-methoxy-1-methylquinolin-1-ium iodide (**2k**)

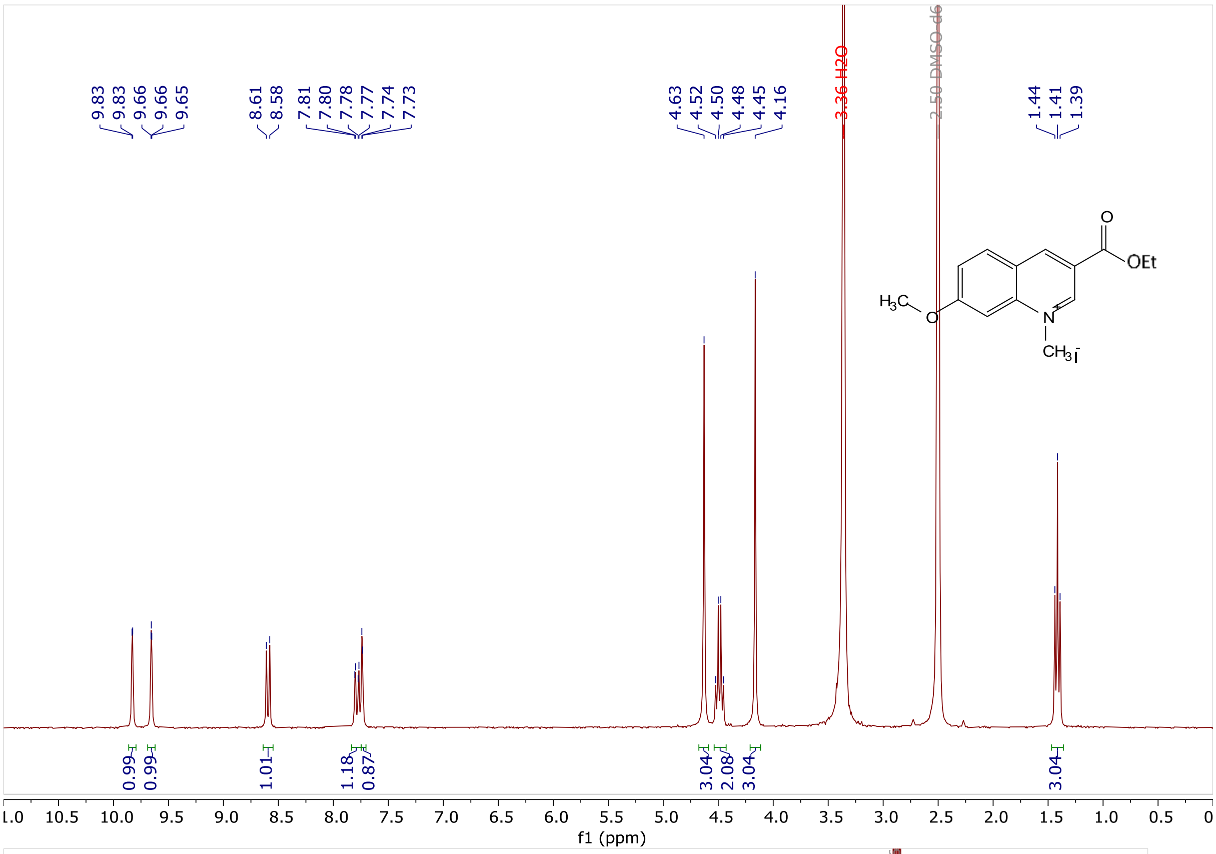

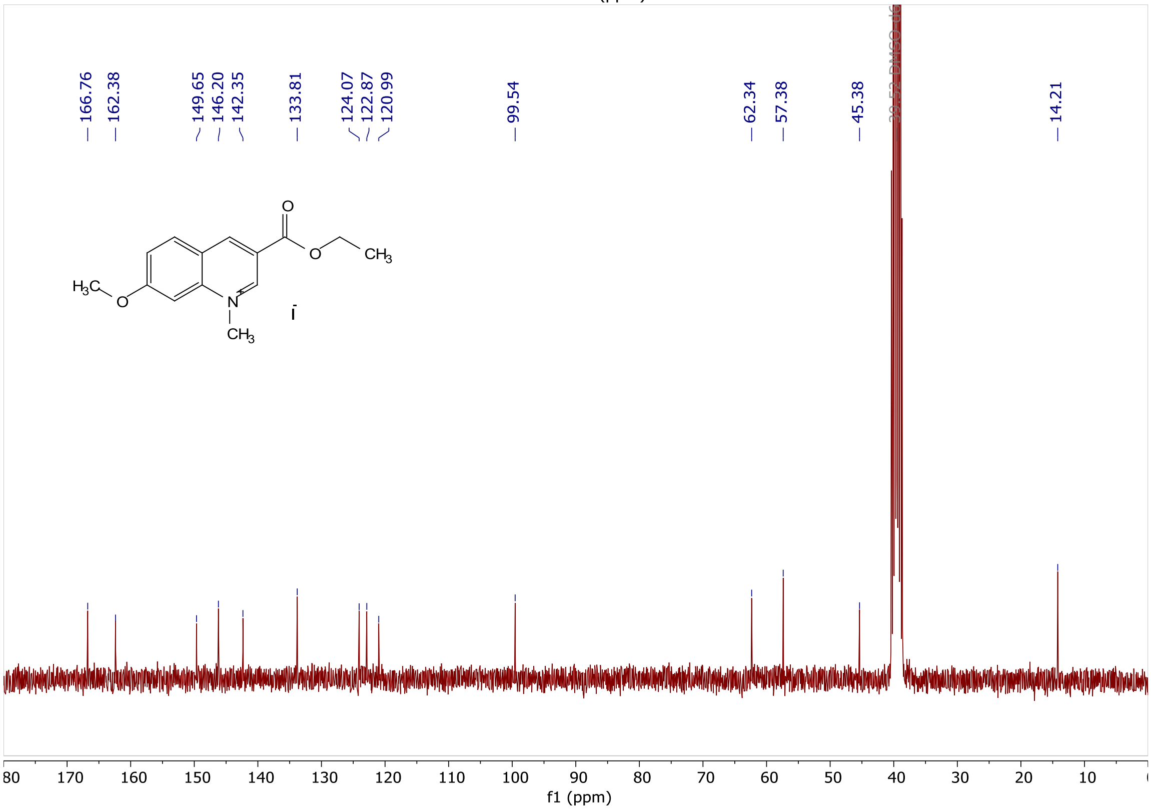

3-(Dimethylcarbamoyl)-7-methoxy-1-methylquinolin-1-ium iodide (**2l**)

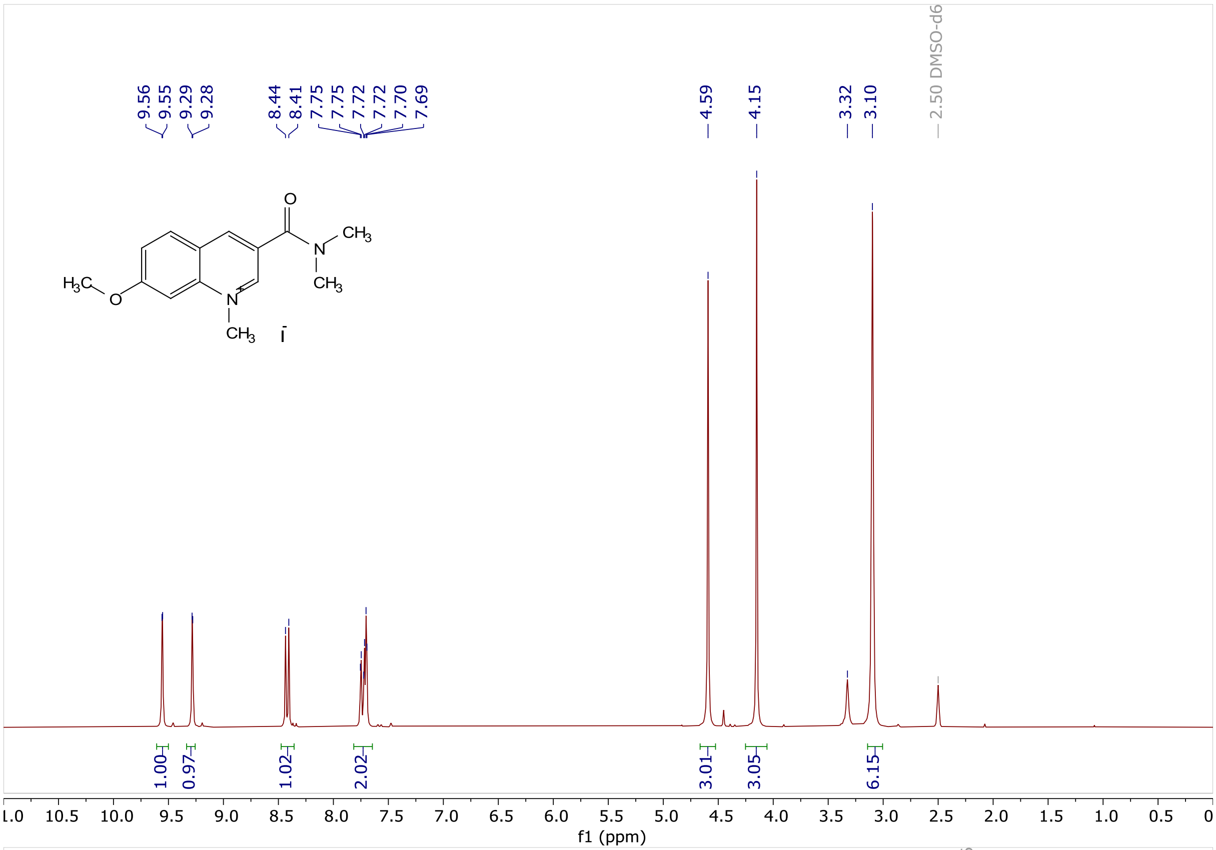

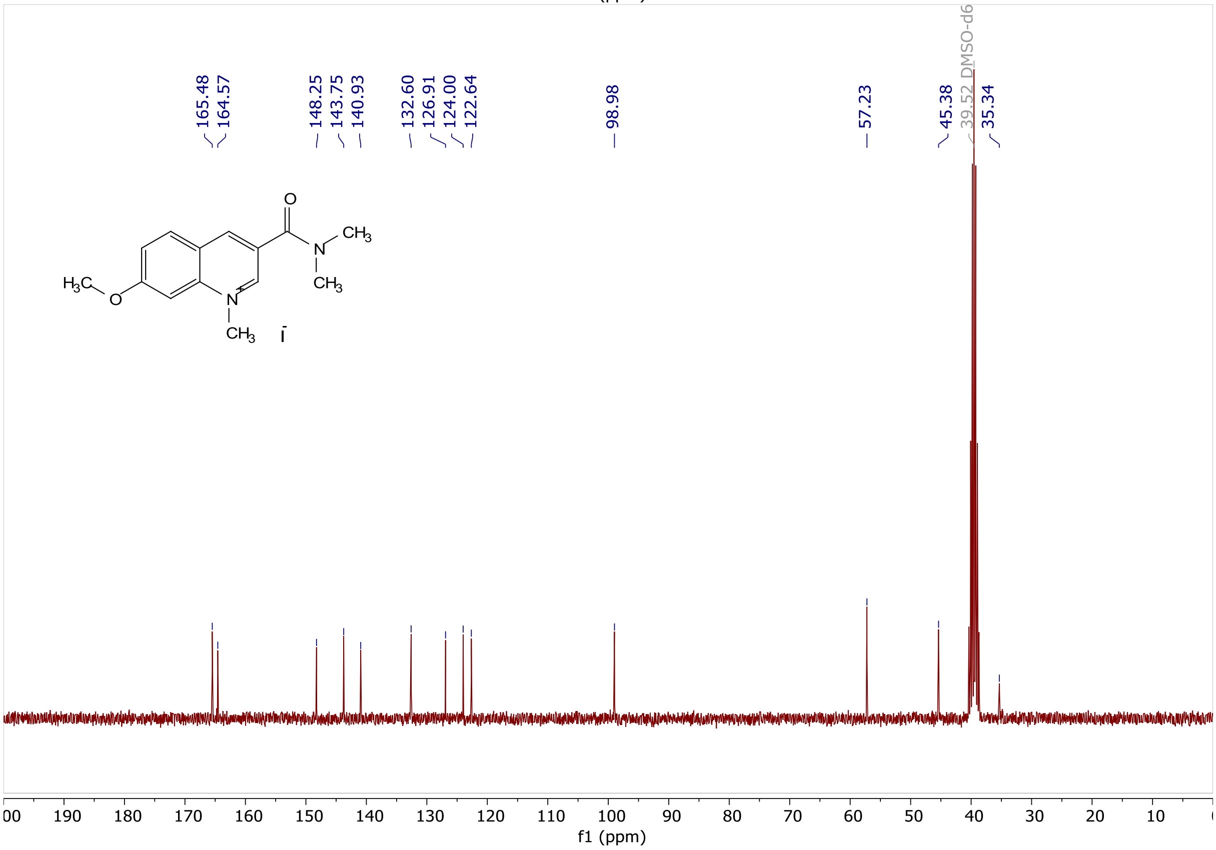

7-Methoxy-3-(methoxy(methyl)carbamoyl)-1-methylquinolin-1-ium iodide (**2m**)

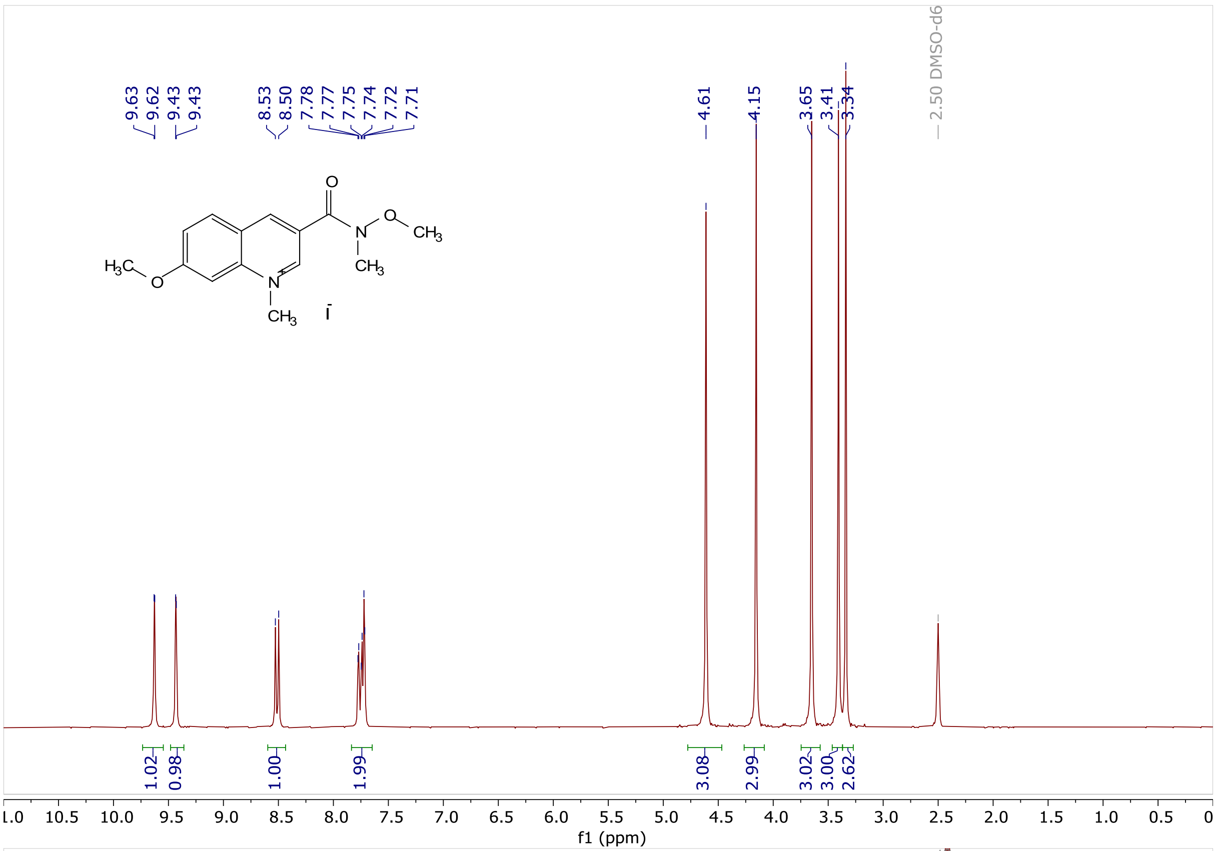

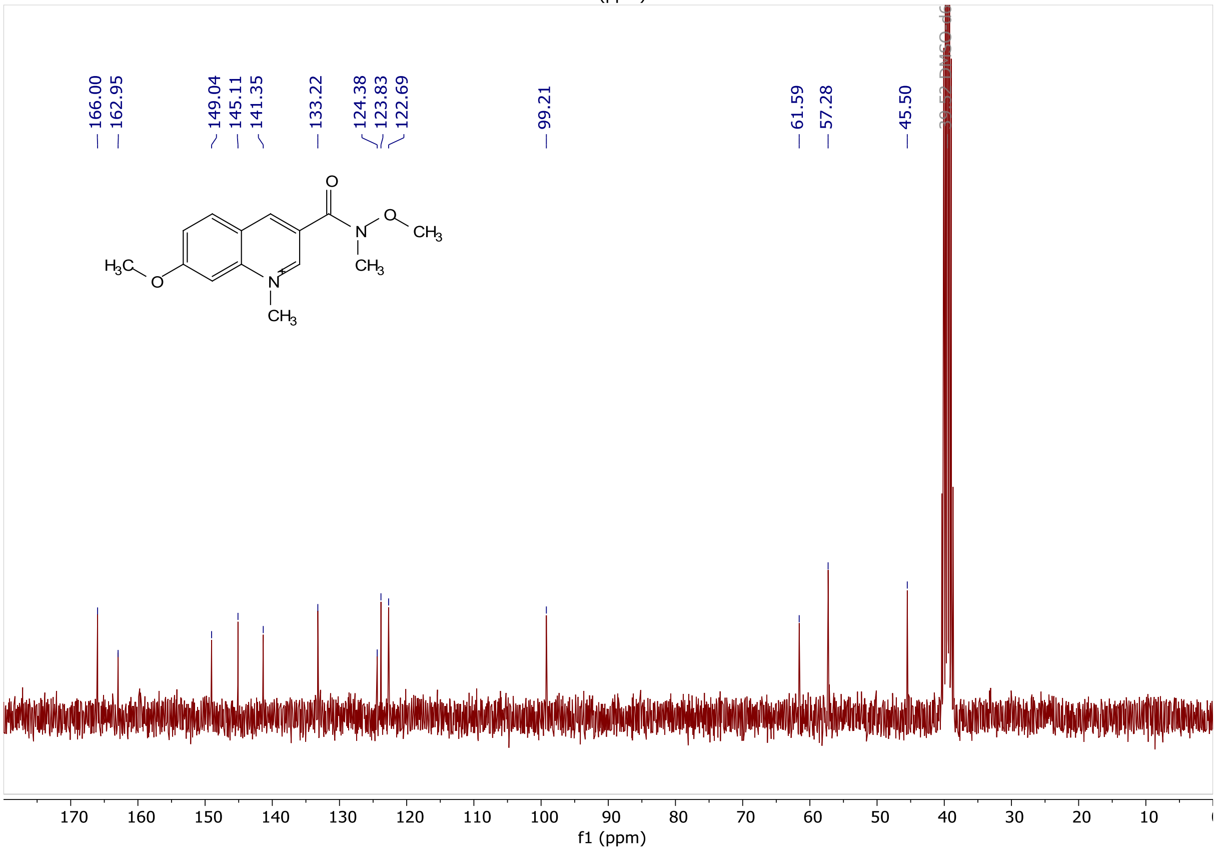

3-Acetyl-1-benzyl-7-methoxyquinolin-1-ium bromide (**2n**)

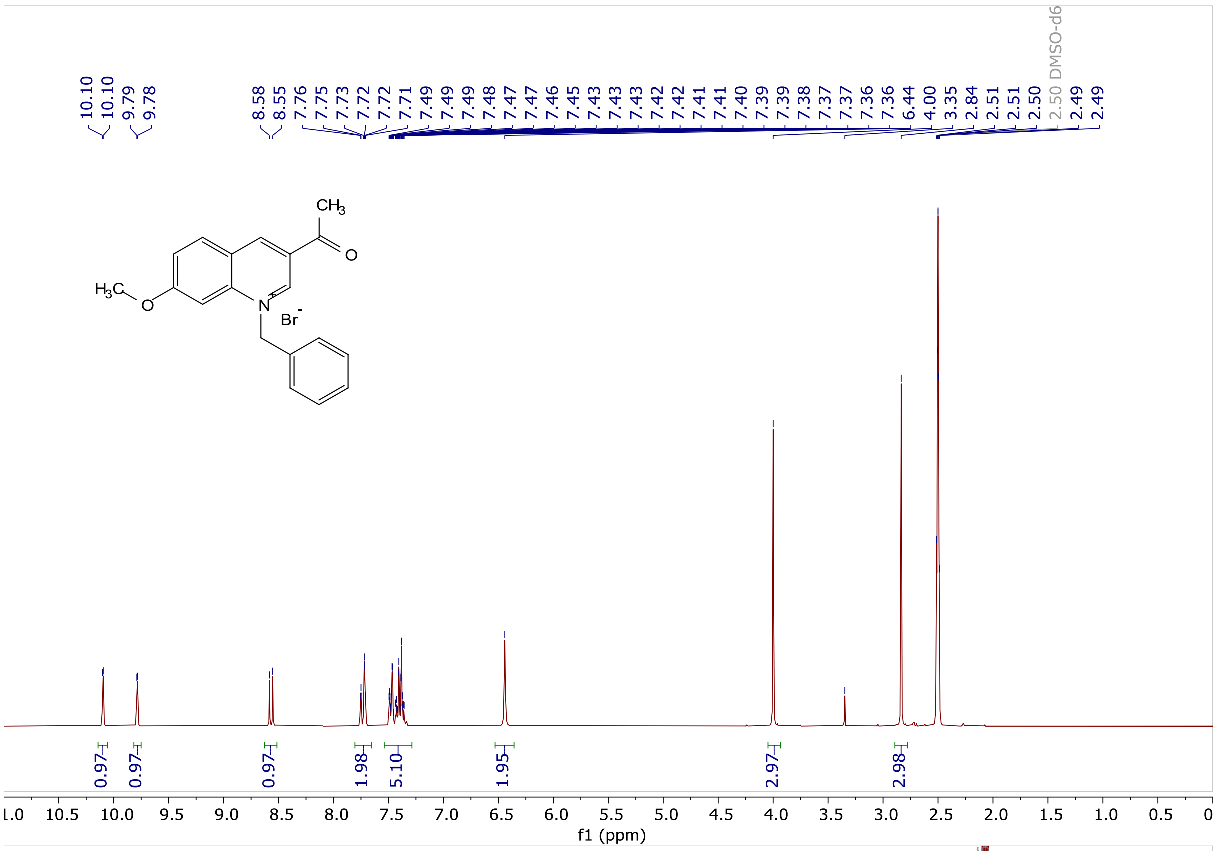

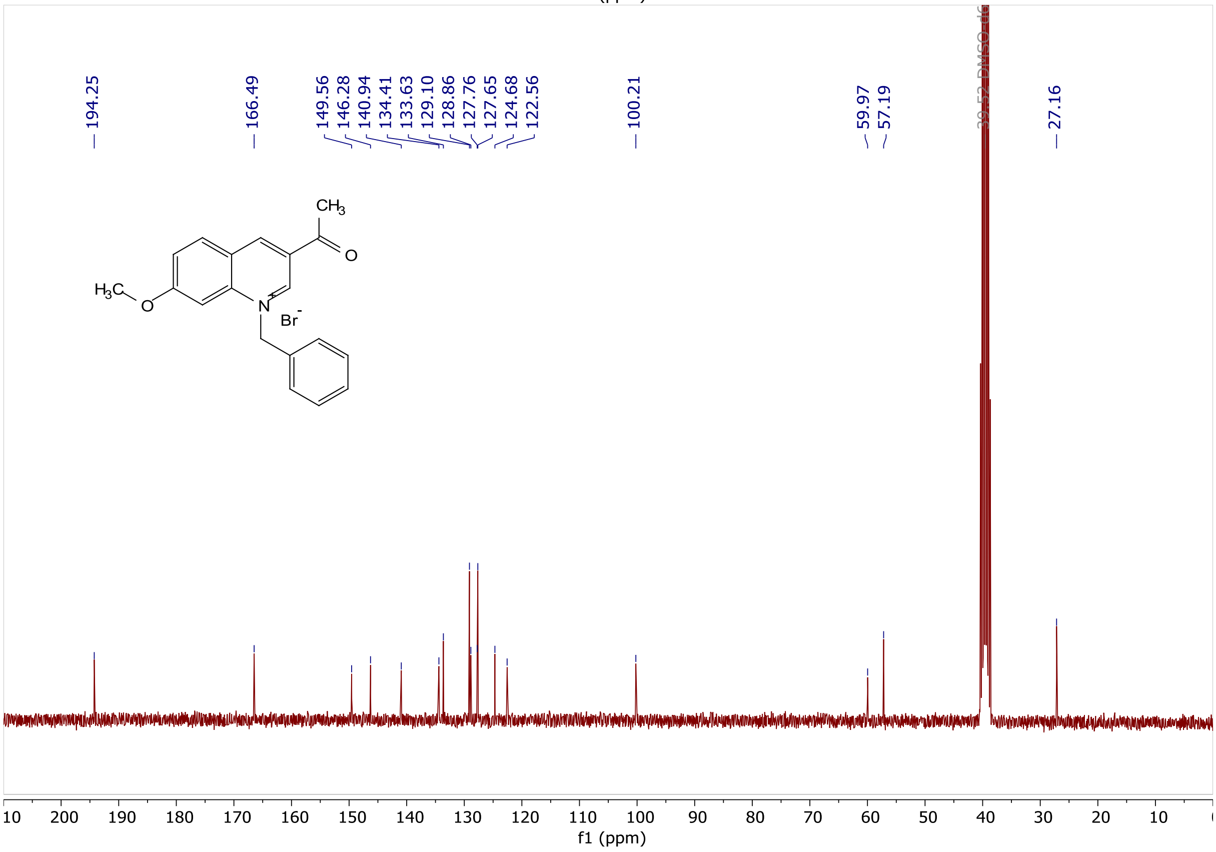

3-Acetyl-7-methoxy-1-phenethylquinolin-1-ium bromide (**2o**)

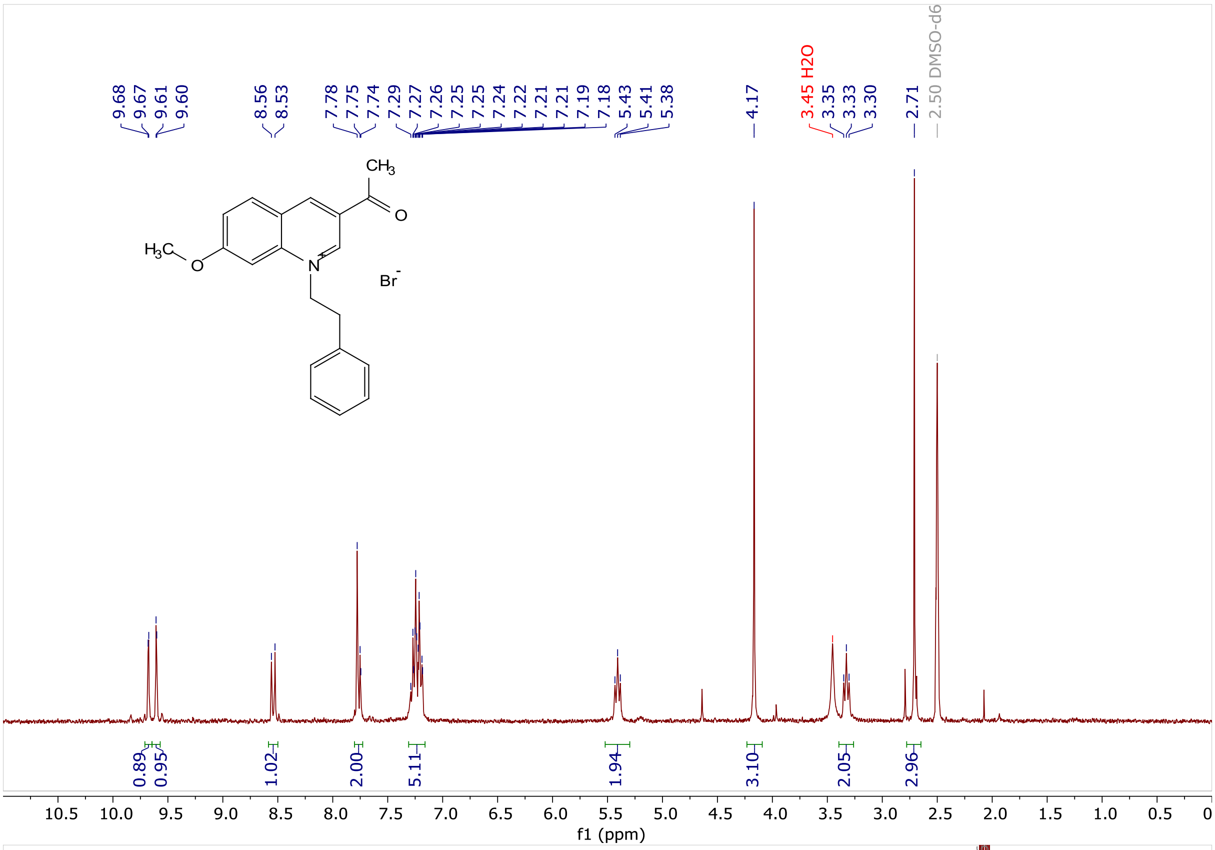

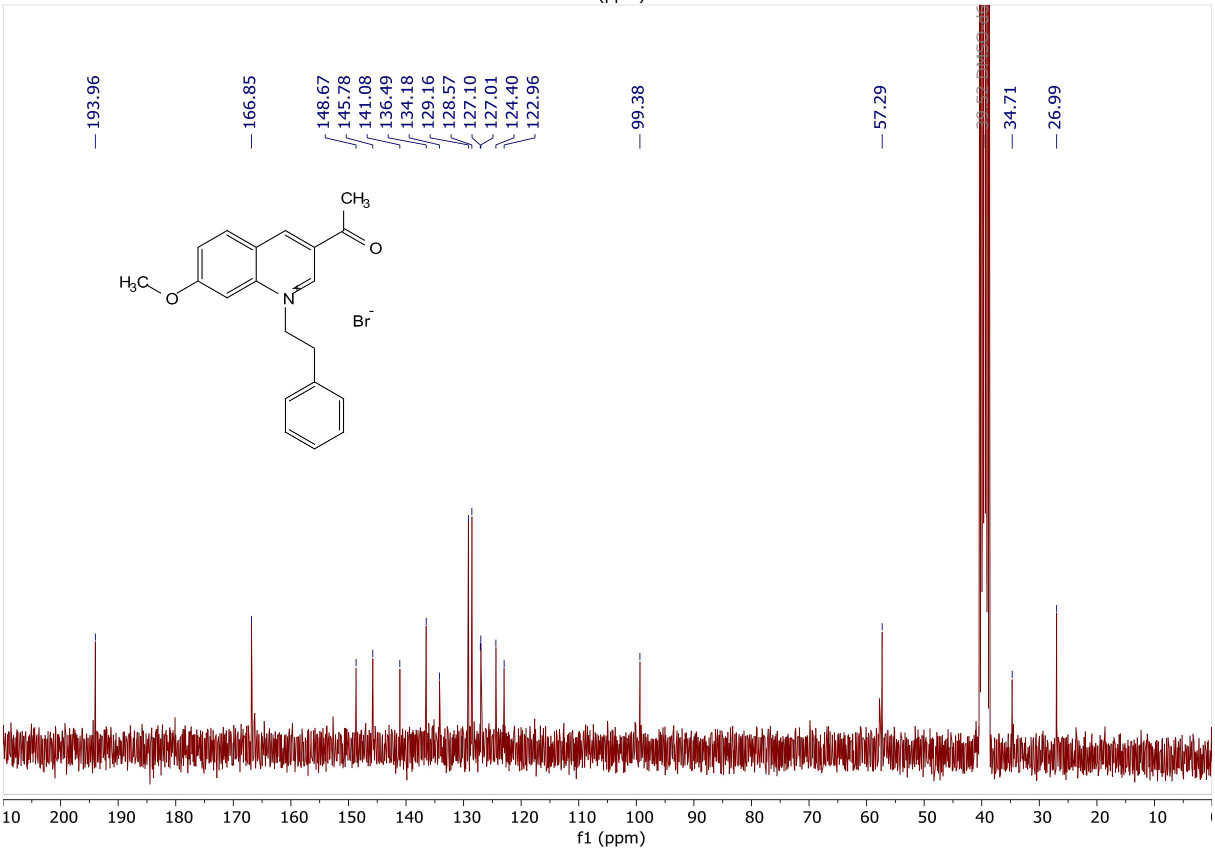

3-Acetyl-7-methoxy-1-propylquinolin-1-ium bromide (**2p**)

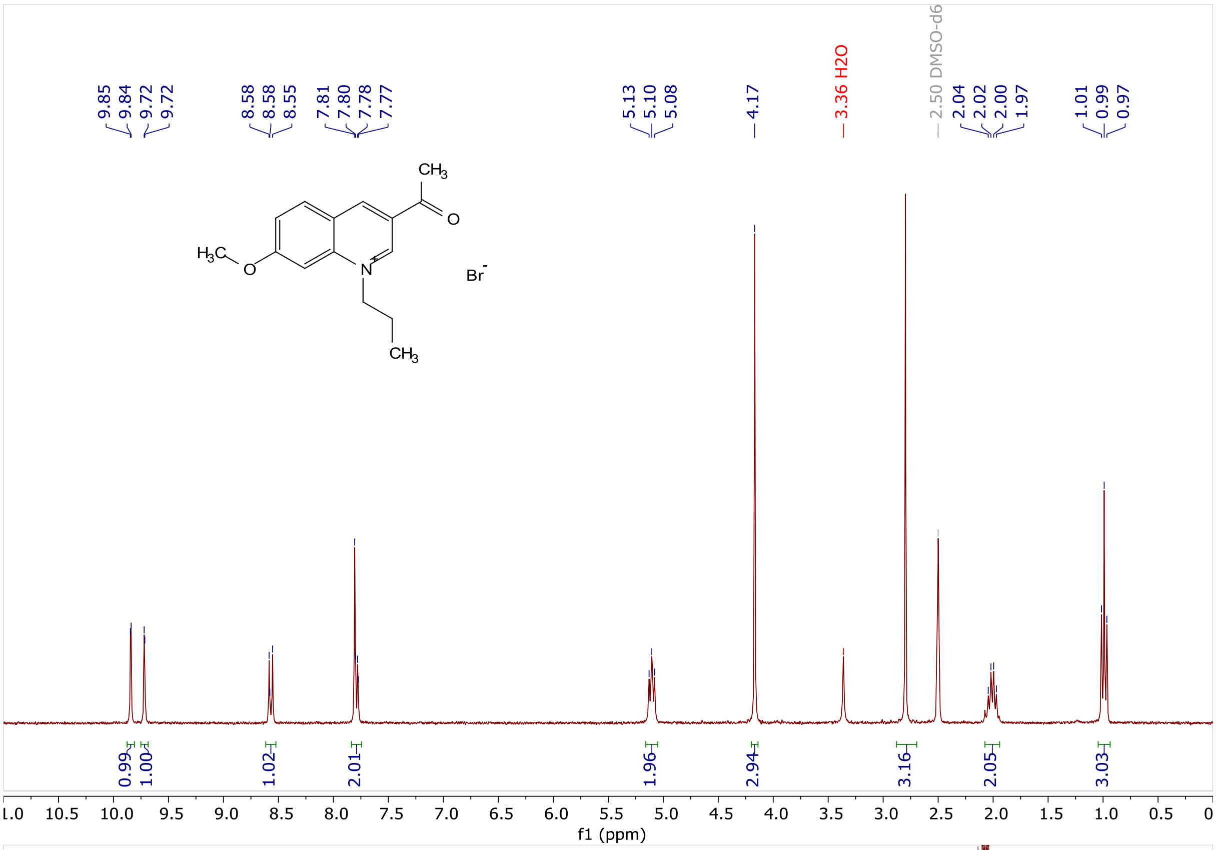

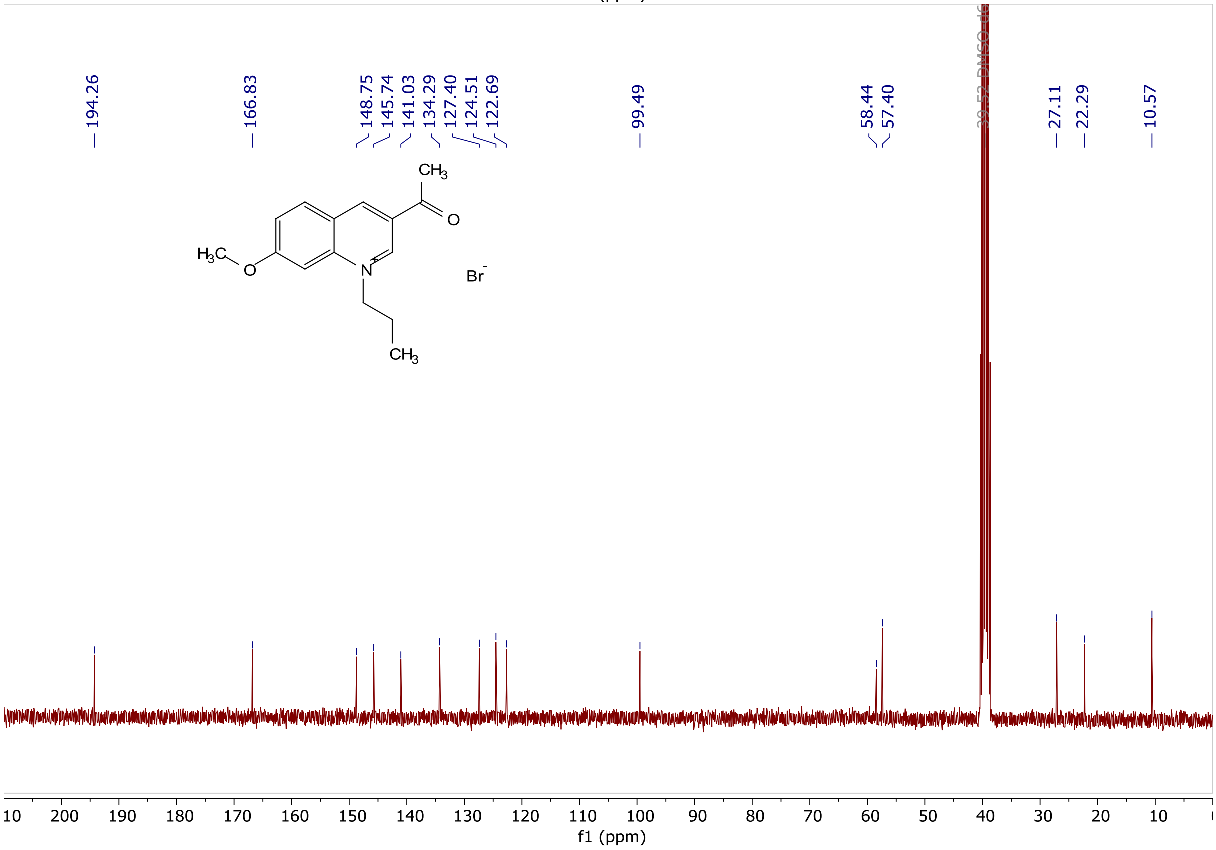

3-Acetyl-7-trifluoromethylquinoline (**6b**)

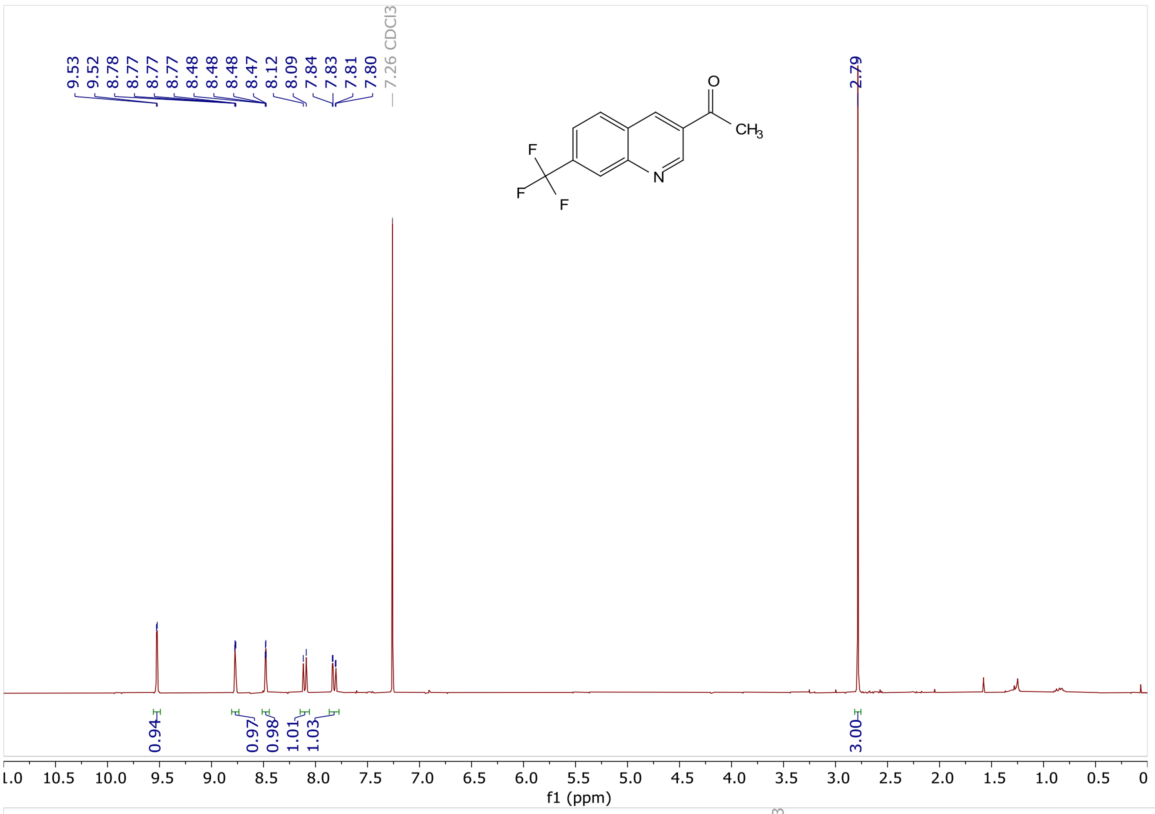

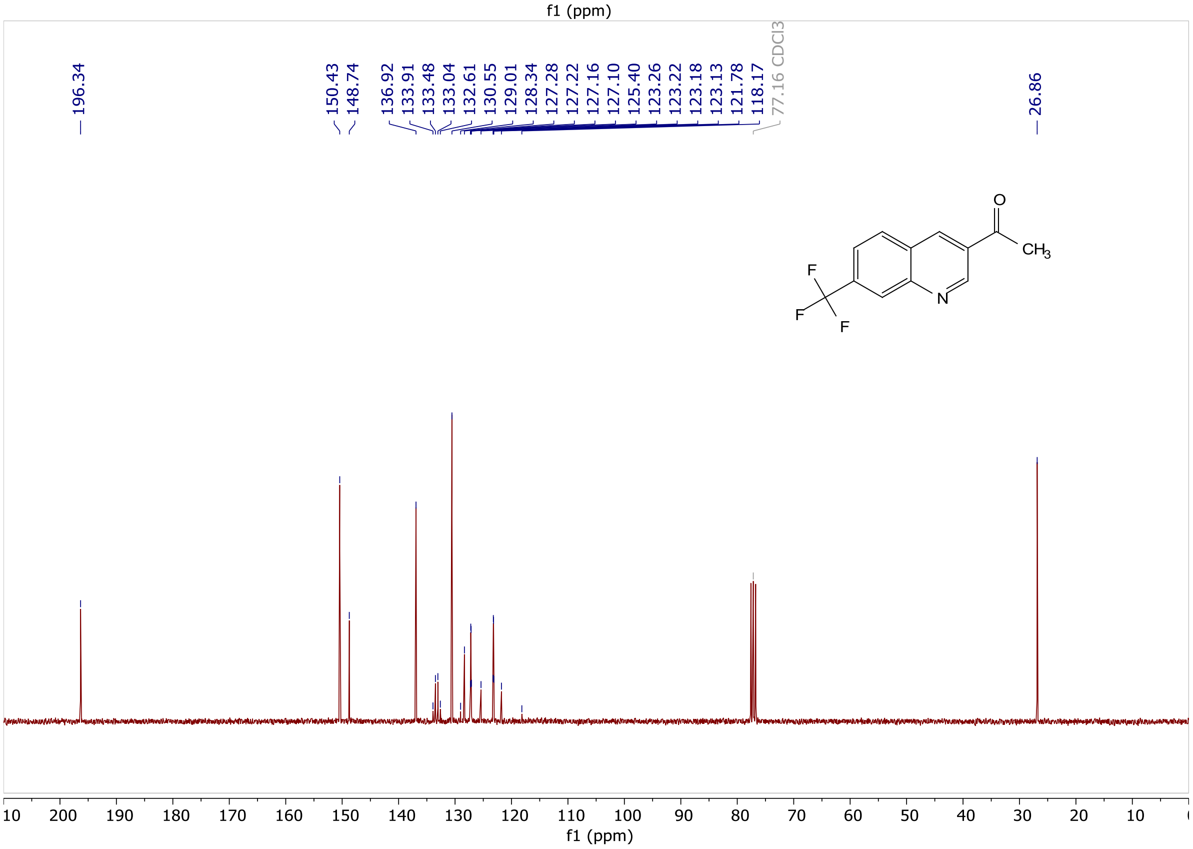

3-Acetyl-7-dimethylaminoquinoline (**6c**)

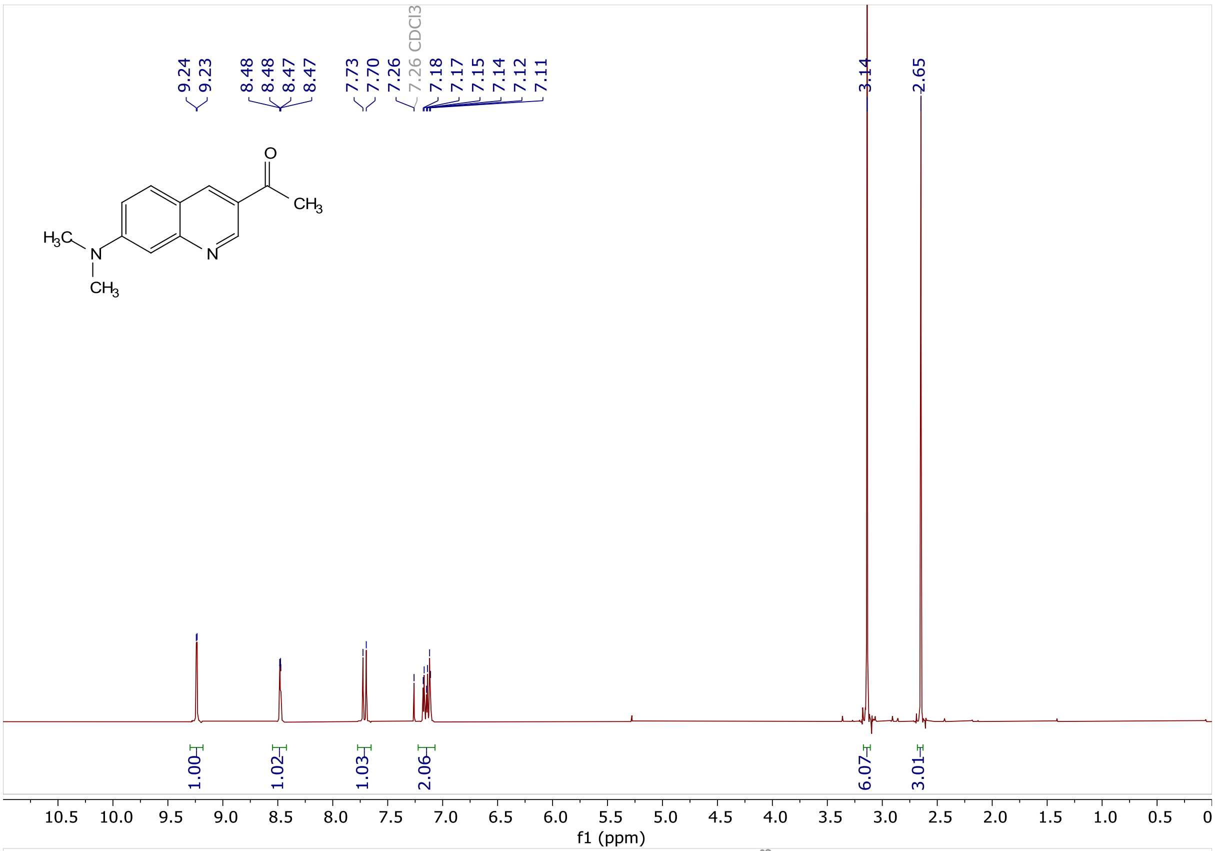

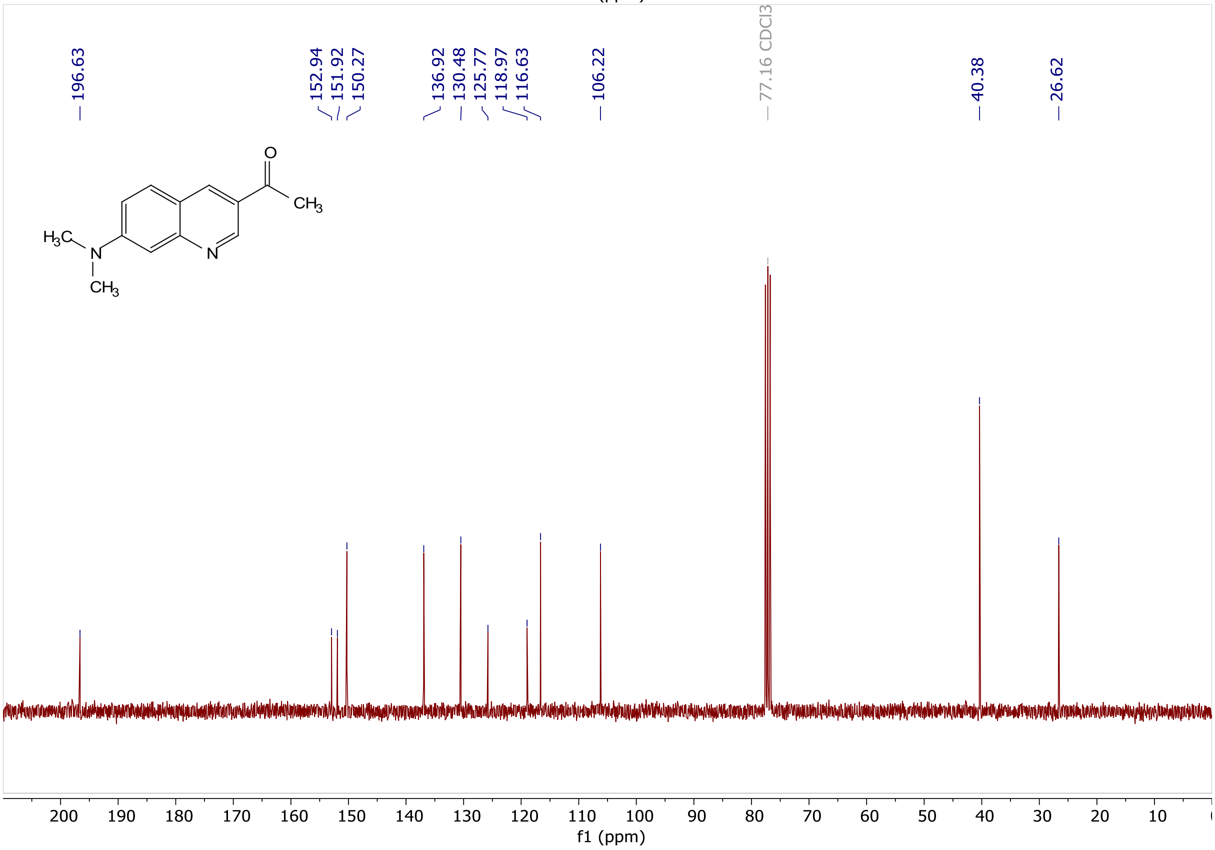

3-Acetyl-7-bromoquinoline (**6d**)

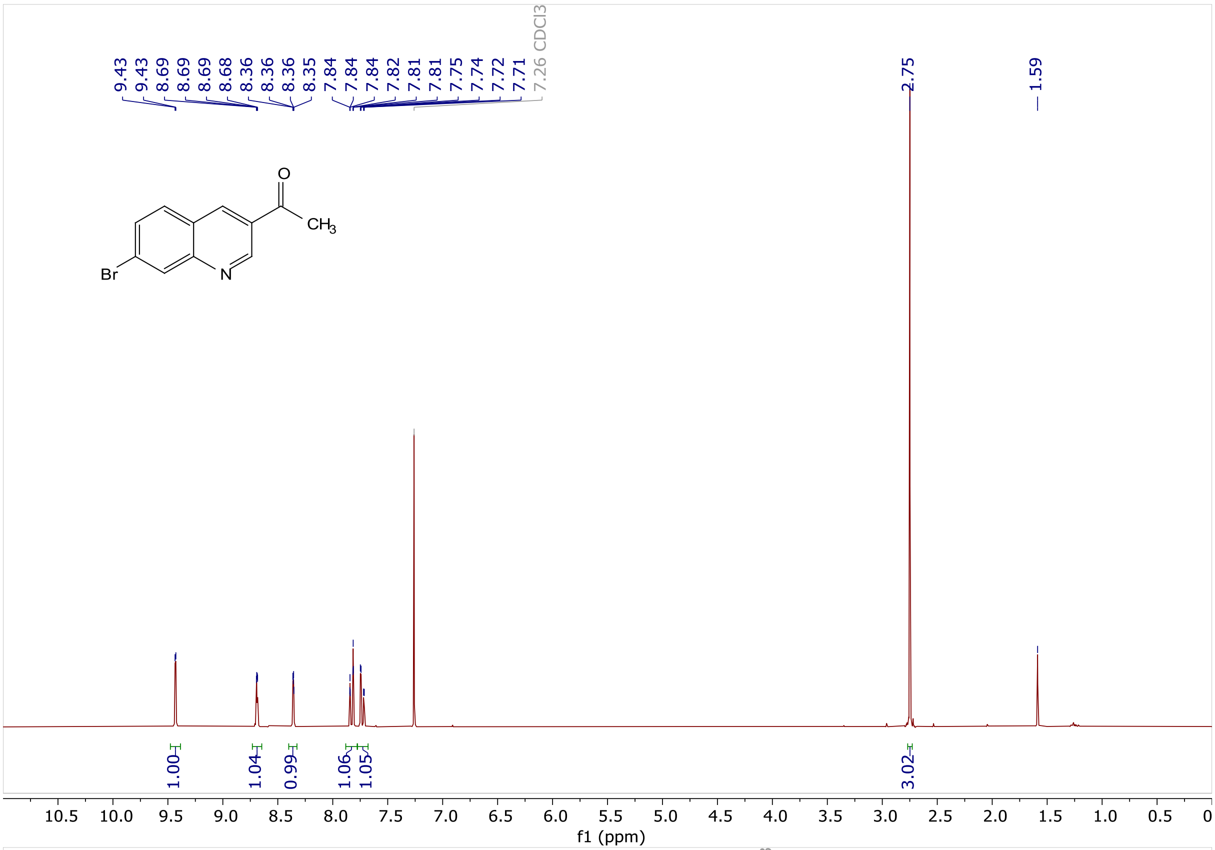

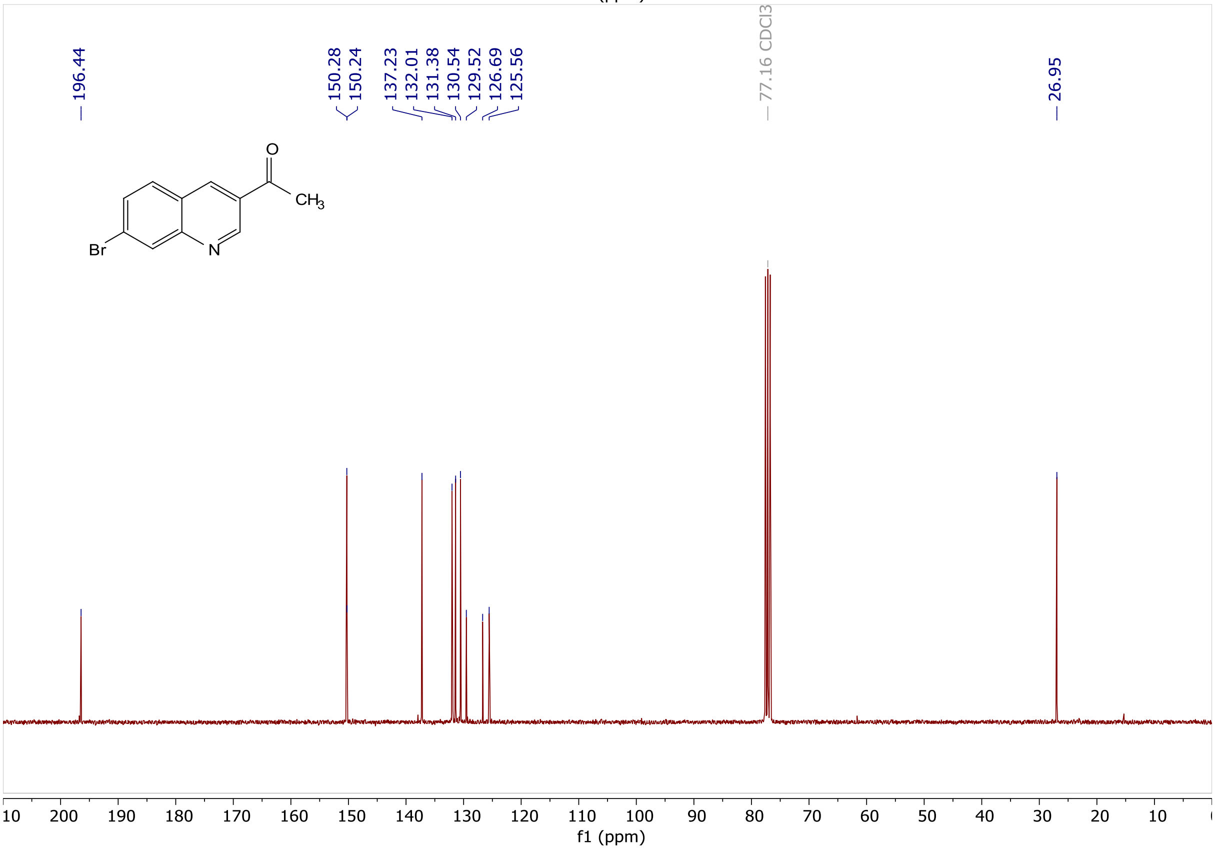

3-Acetyl-6-chloroquinoline (**6e**)

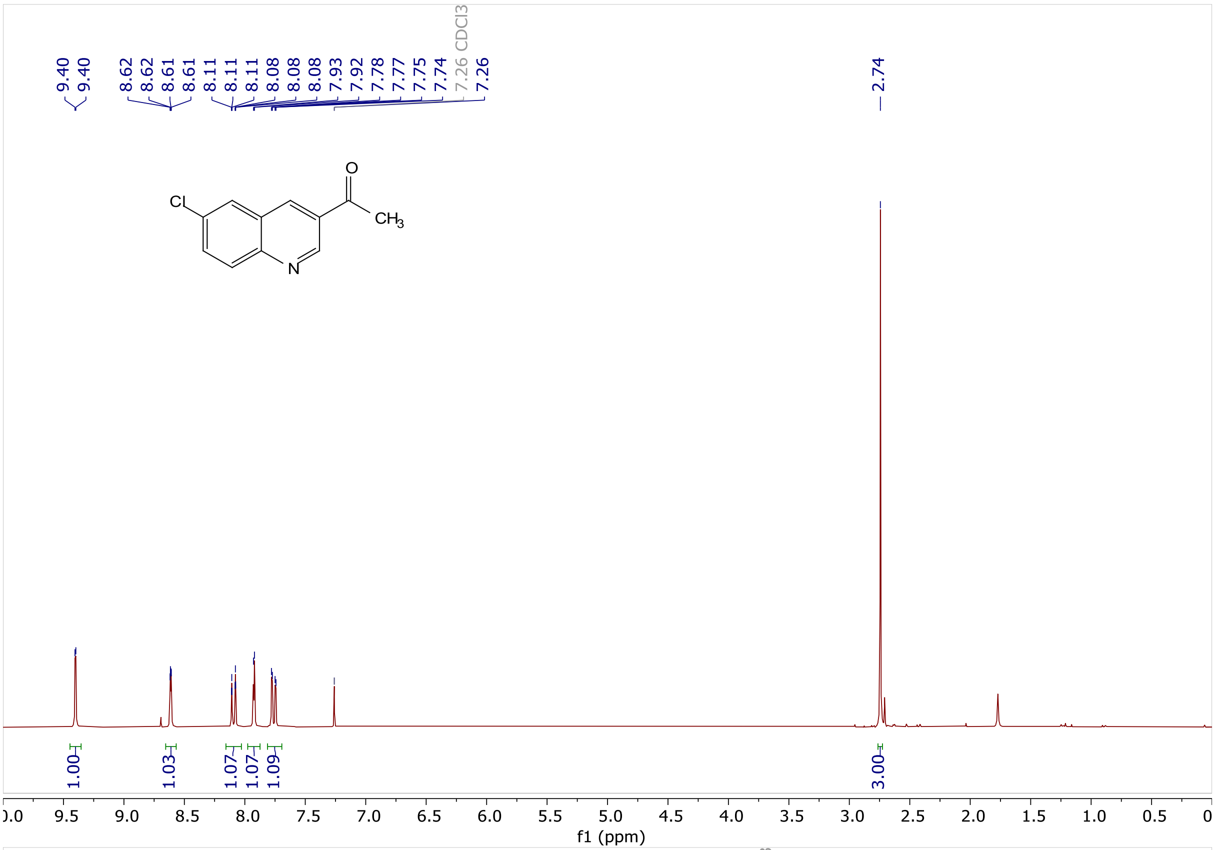

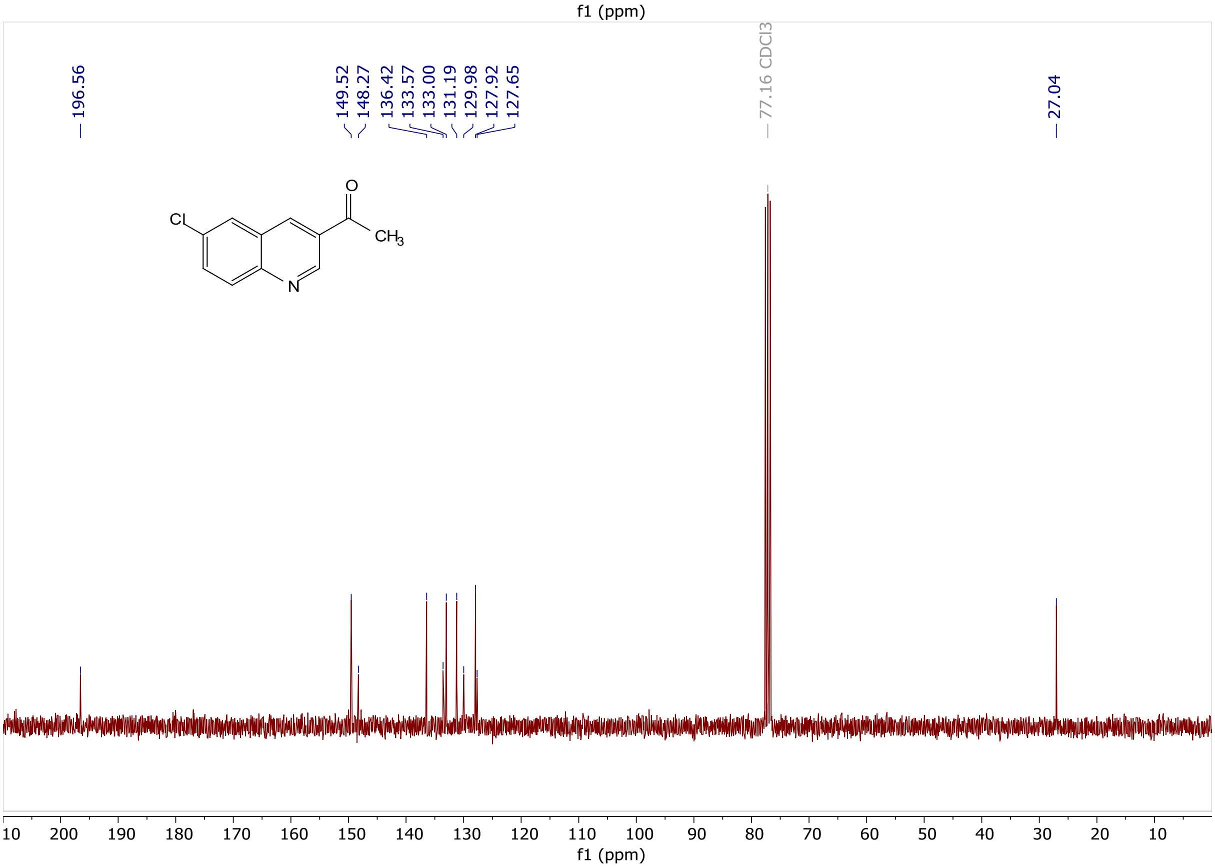

3-Acetyl-6,7-dimethoxyquinoline (**6f**)

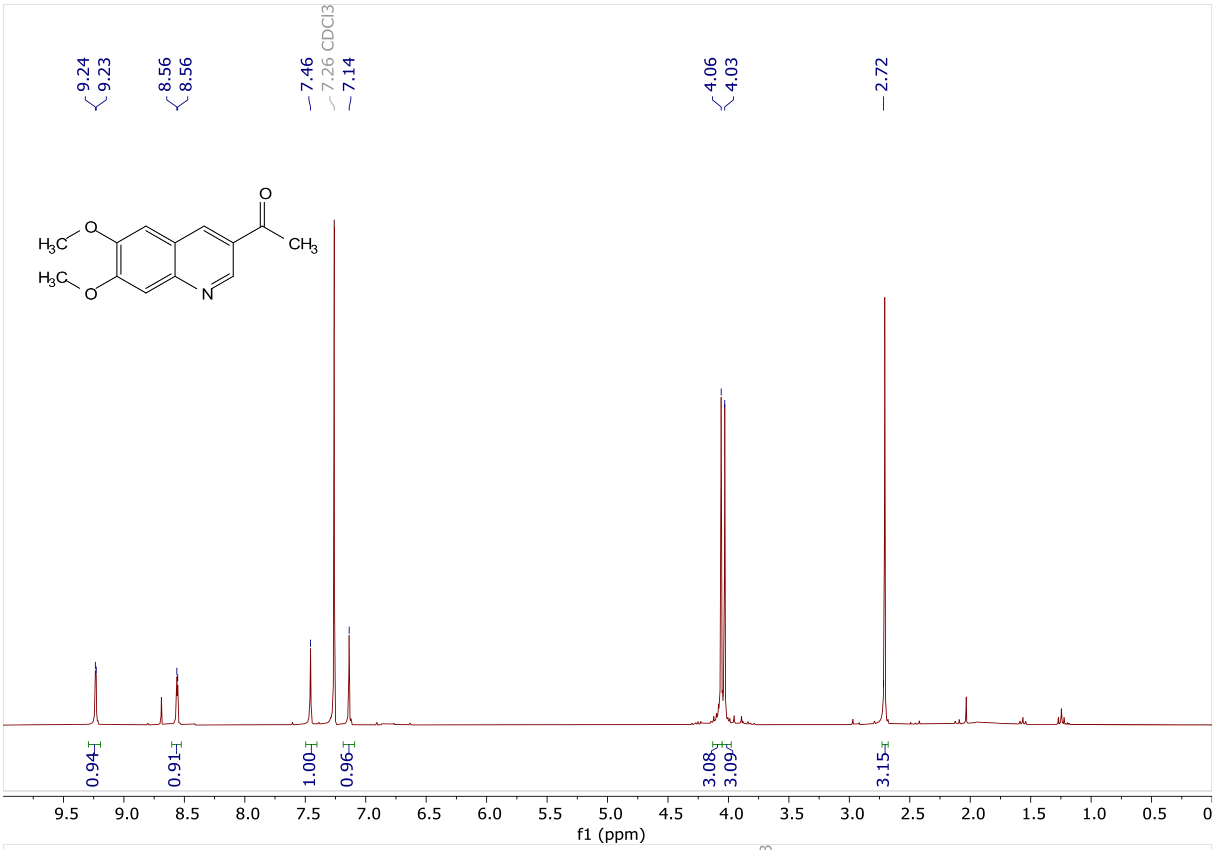

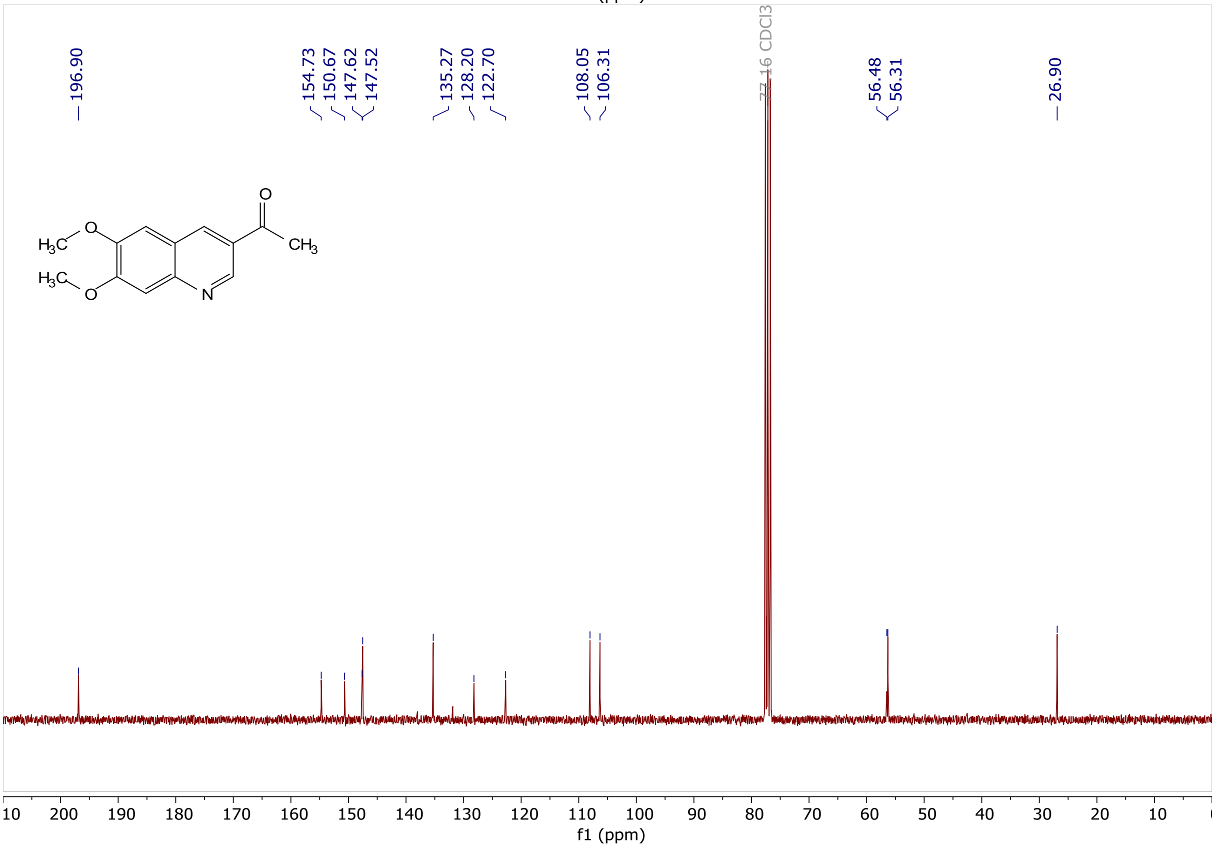

7-Acetyl-[1,3]dioxolo[4,5-g]quinoline (**6g**)

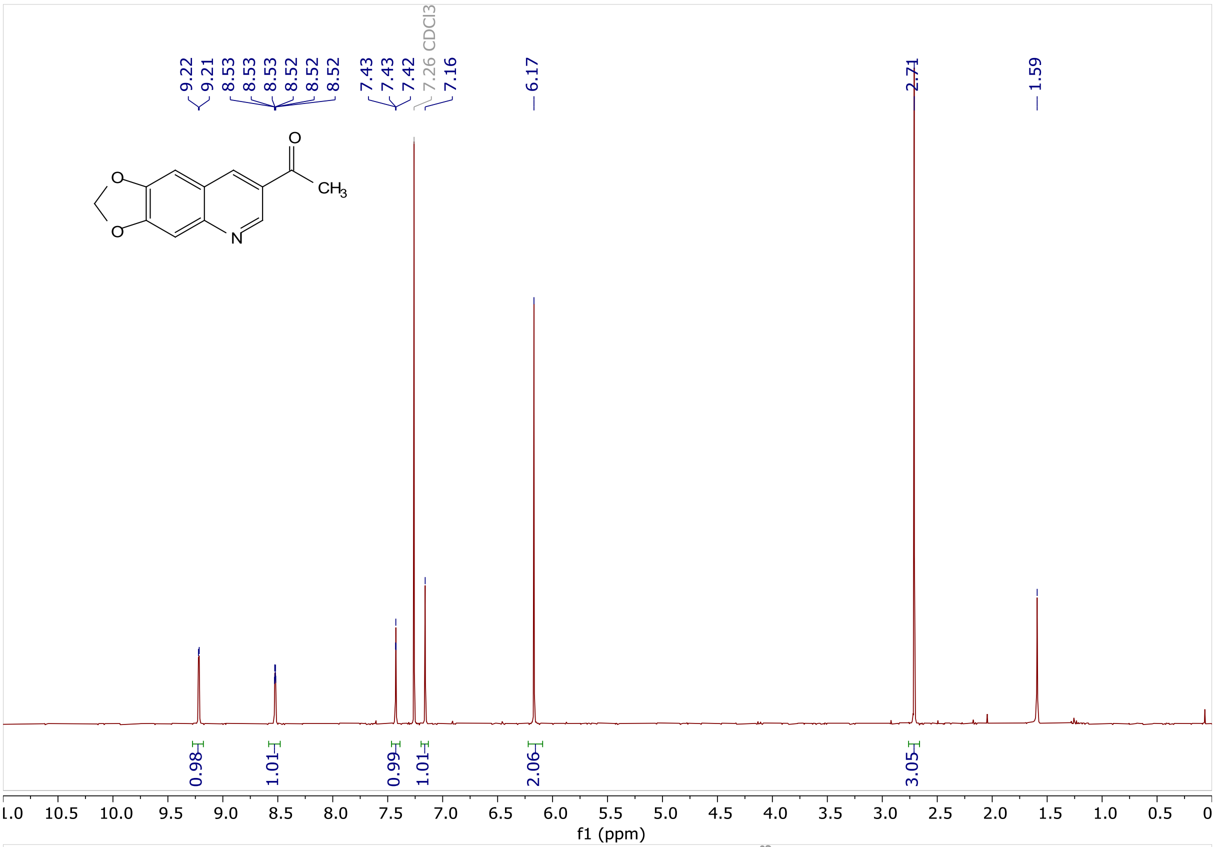

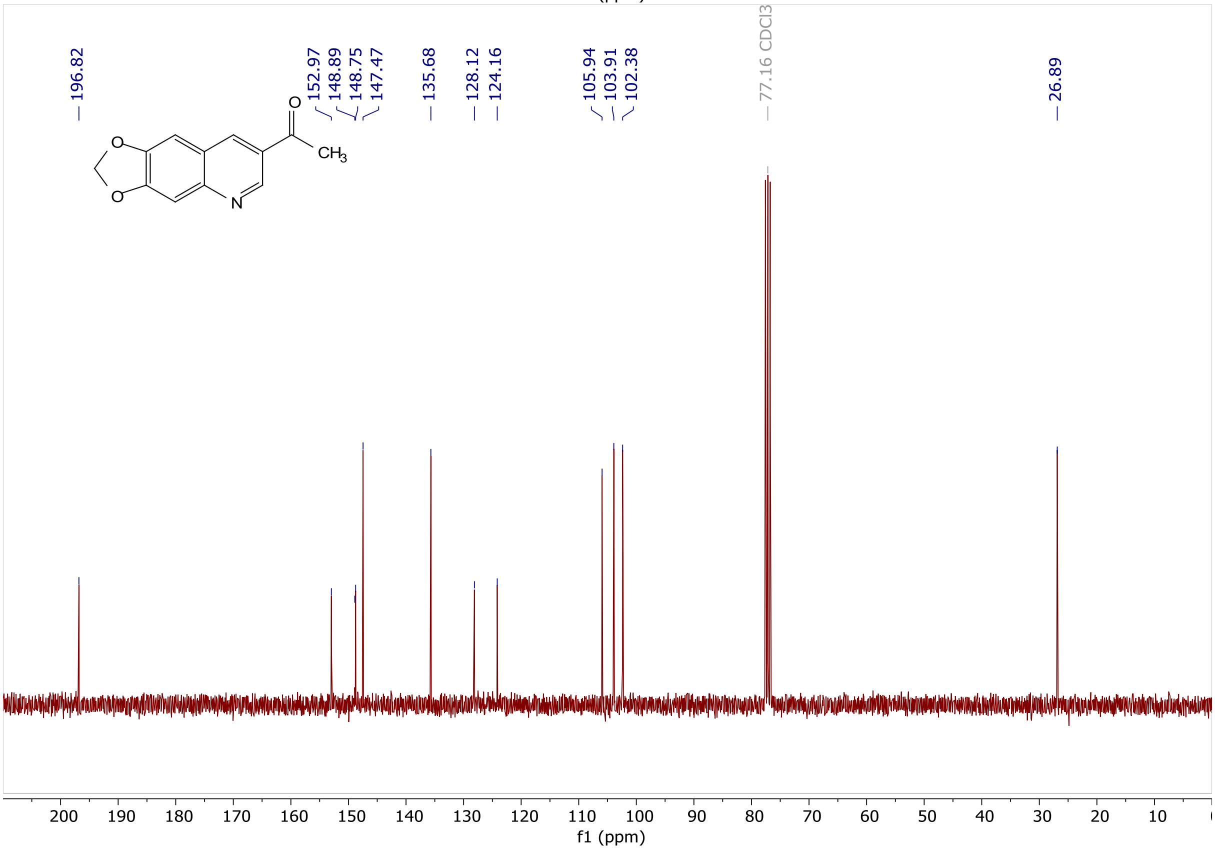

3-Acetyl-7-methoxyquinoline (**6h**)

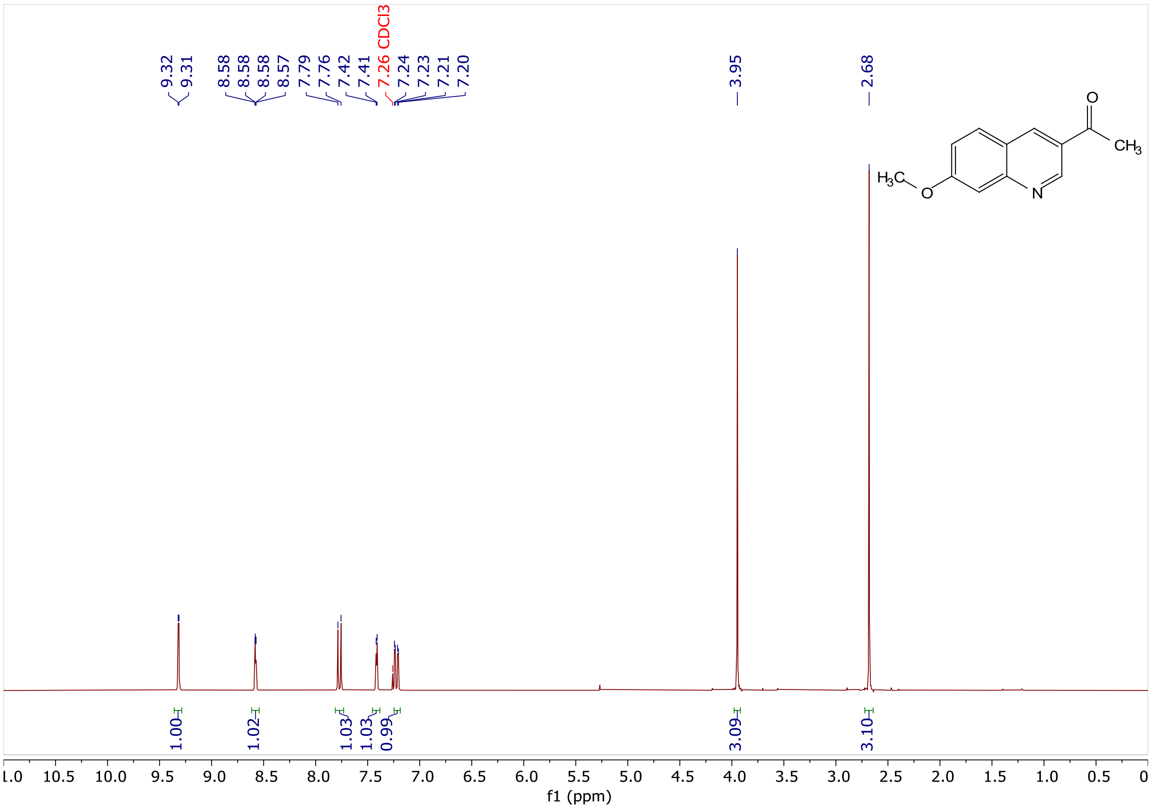

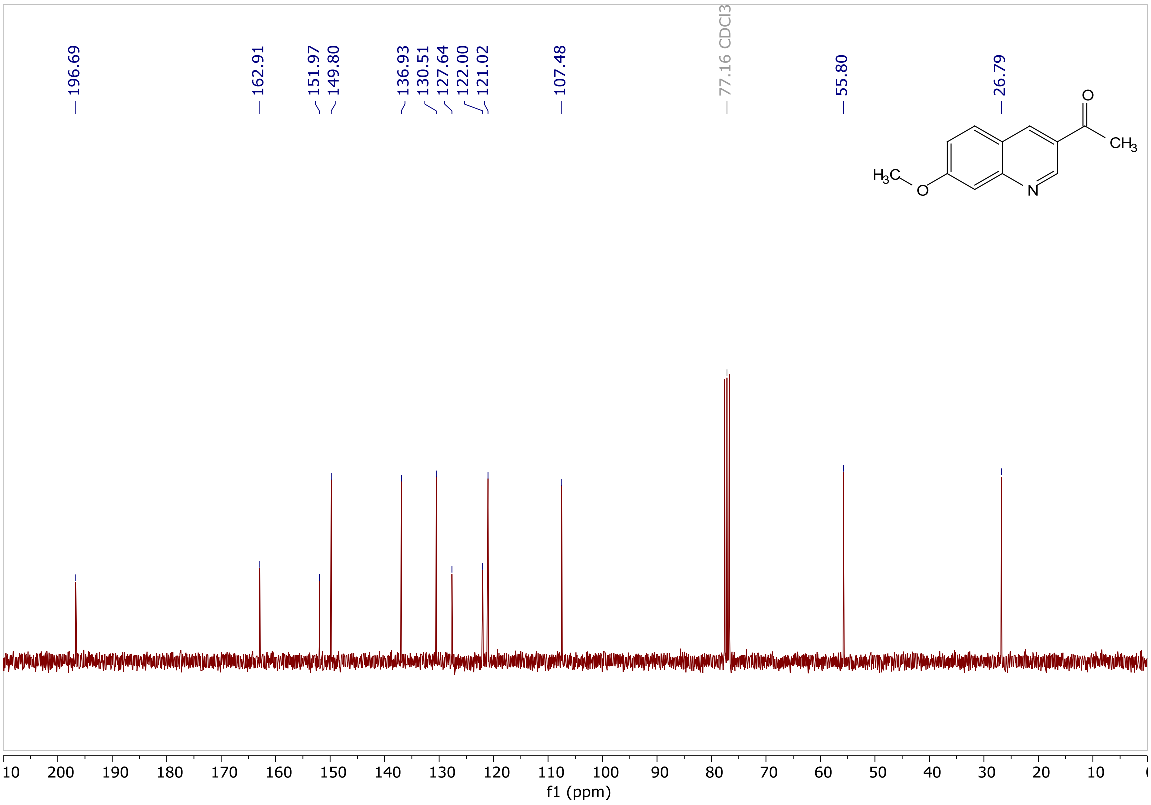

Methyl 7-methoxyquinoline-3-carboxylate (**6j**)

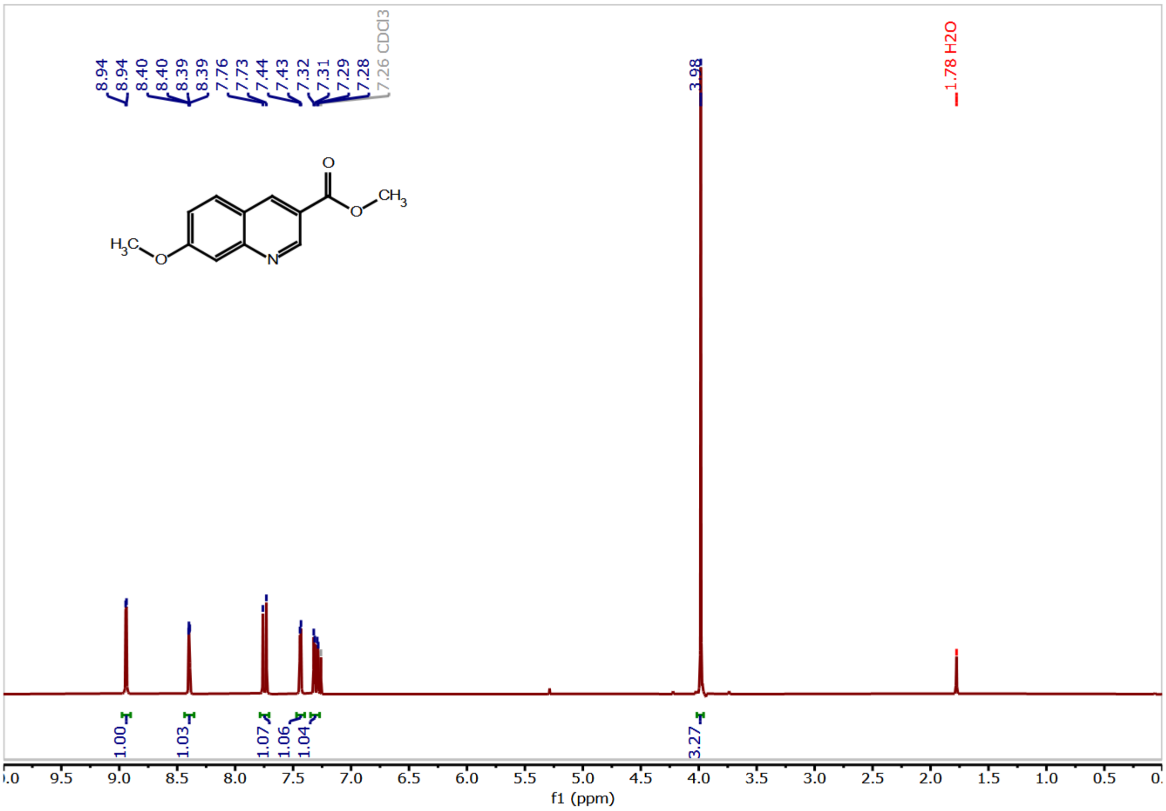

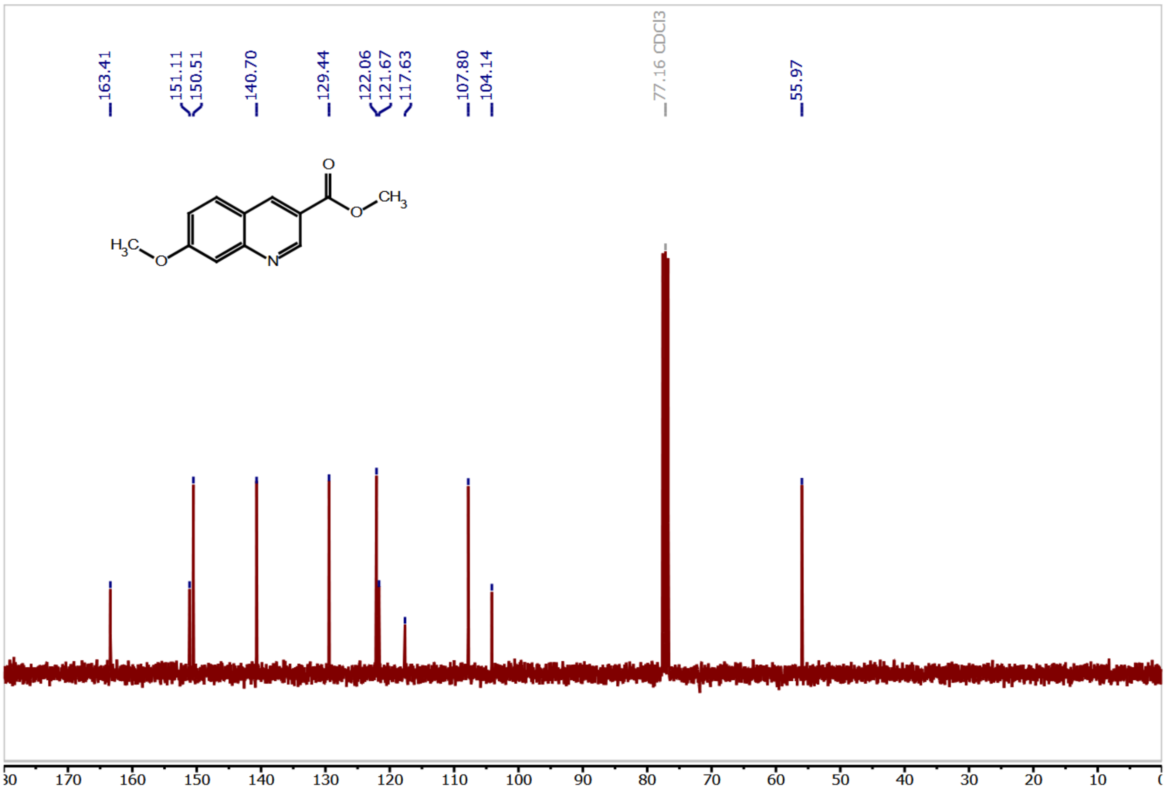

Ethyl 7-methoxy-3-carboxylate (**6k**)

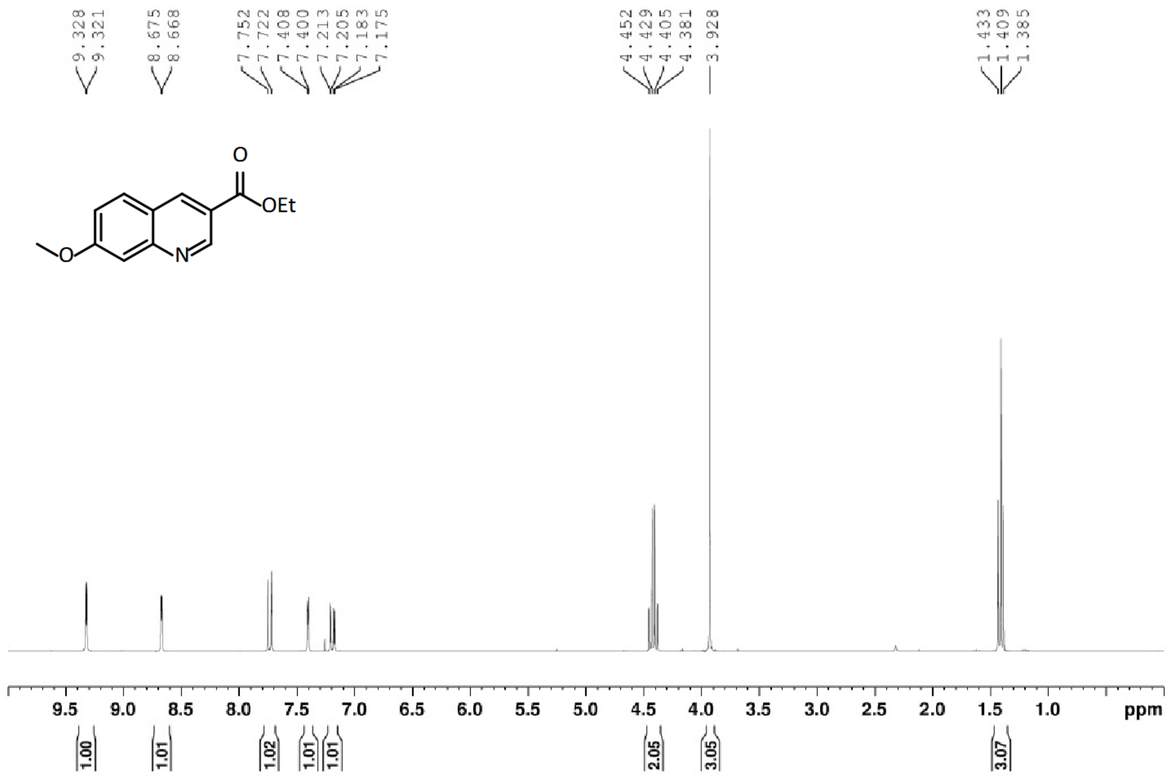

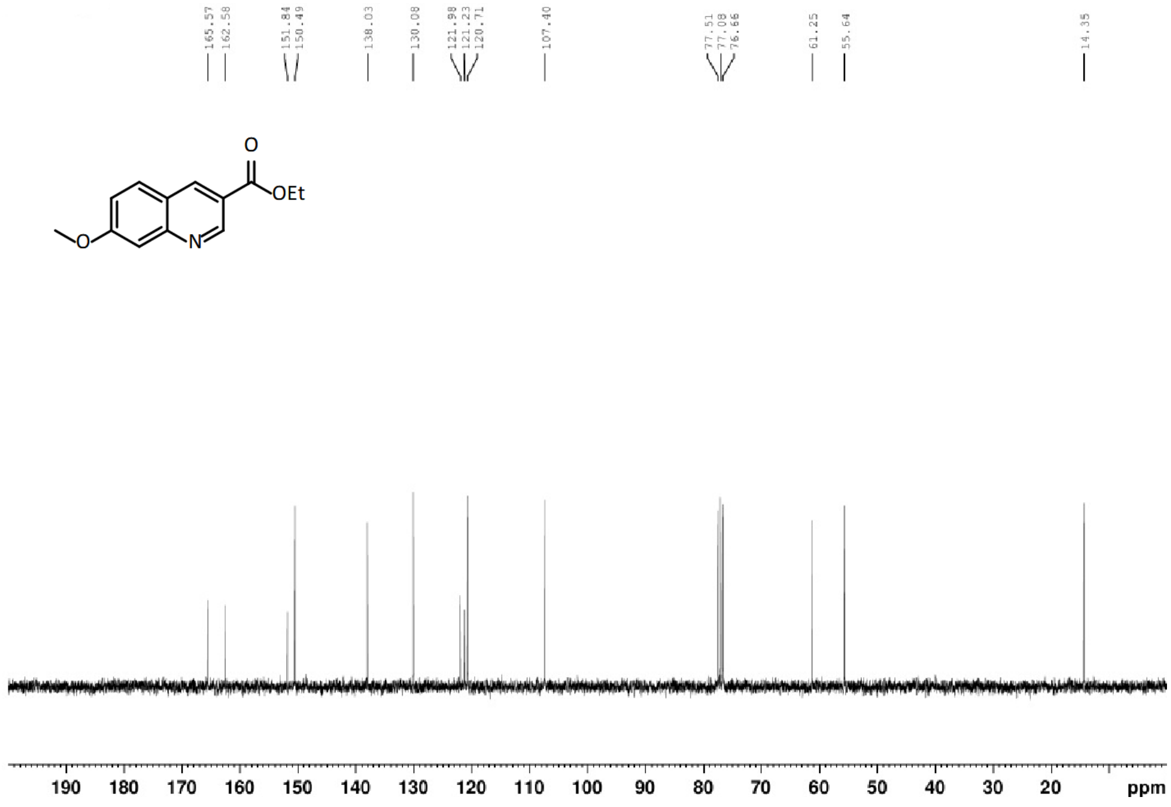

7-Methoxy-N,N-dimethylquinoline-3-carboxamide (**6l**)

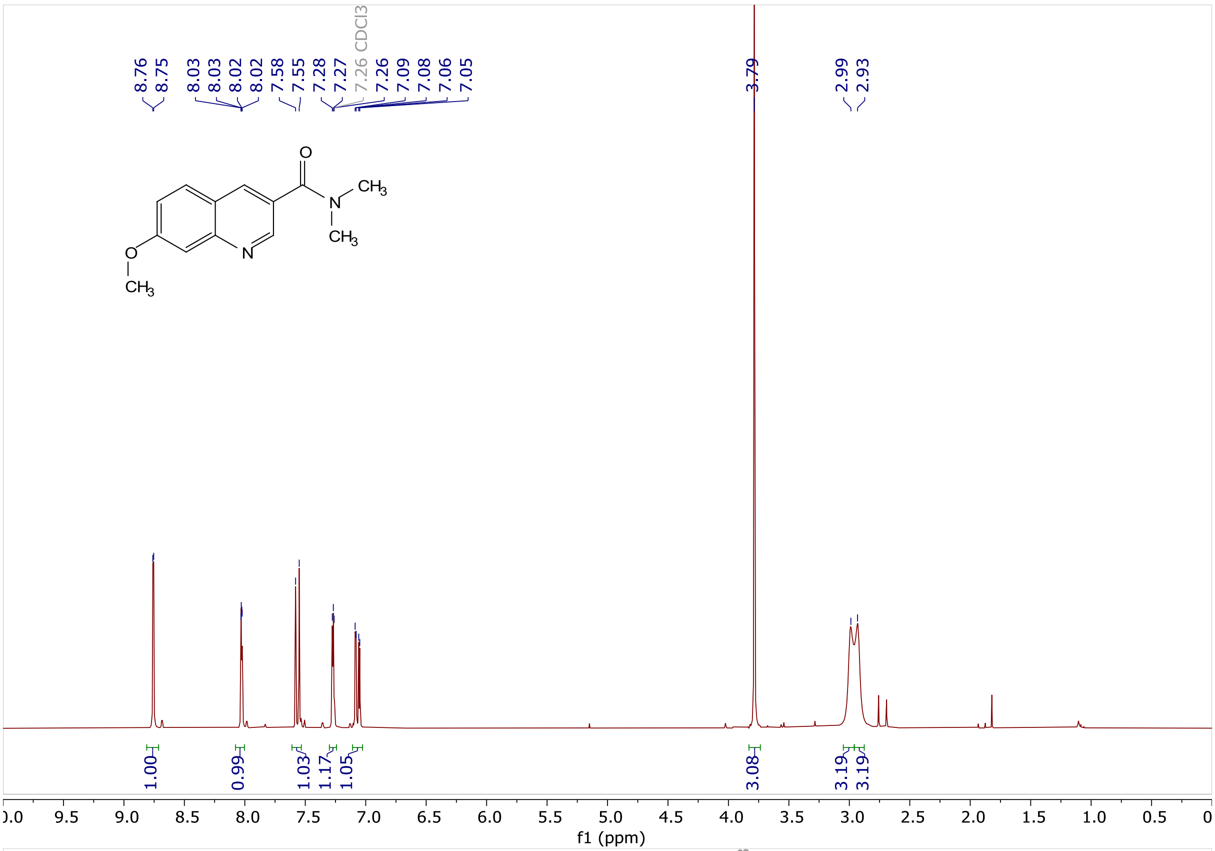

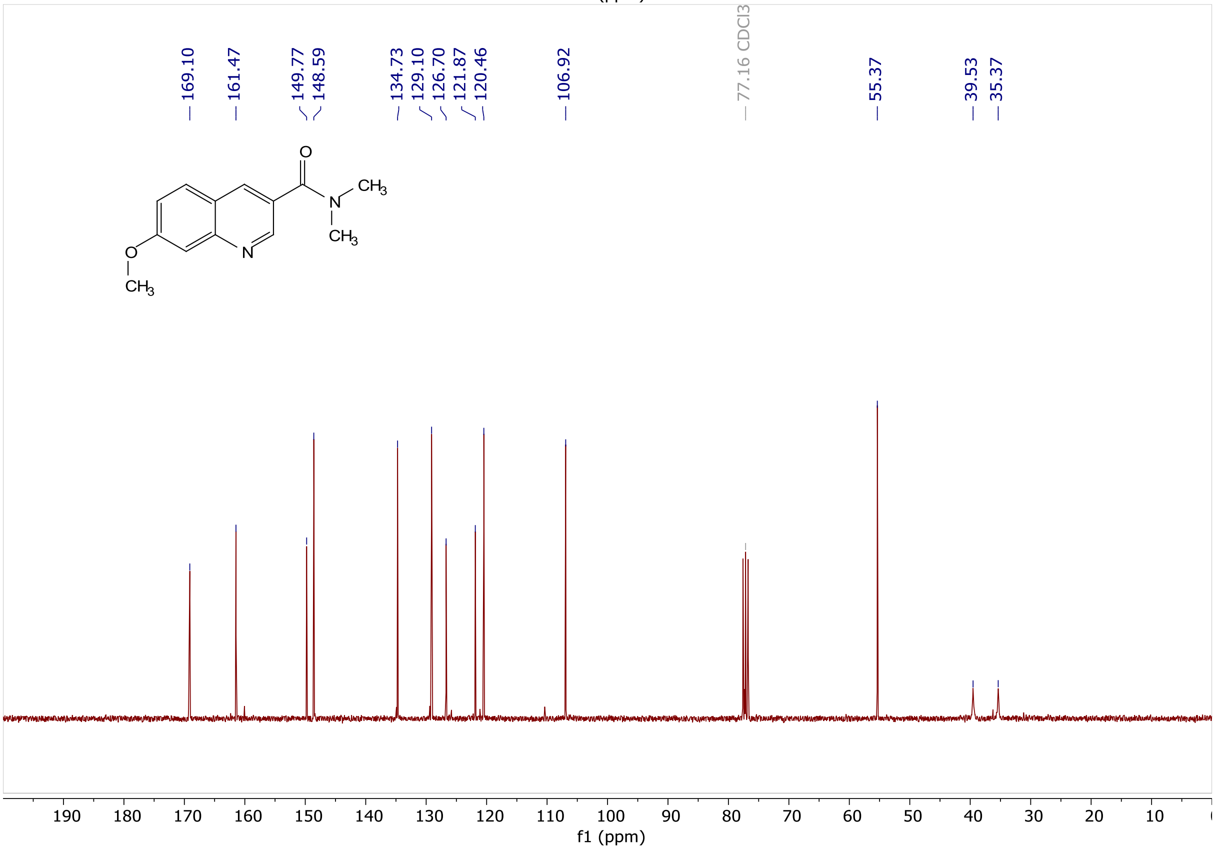

N,7-Dimethoxy-N-methylquinoline-3-carboxamide (**6m**)

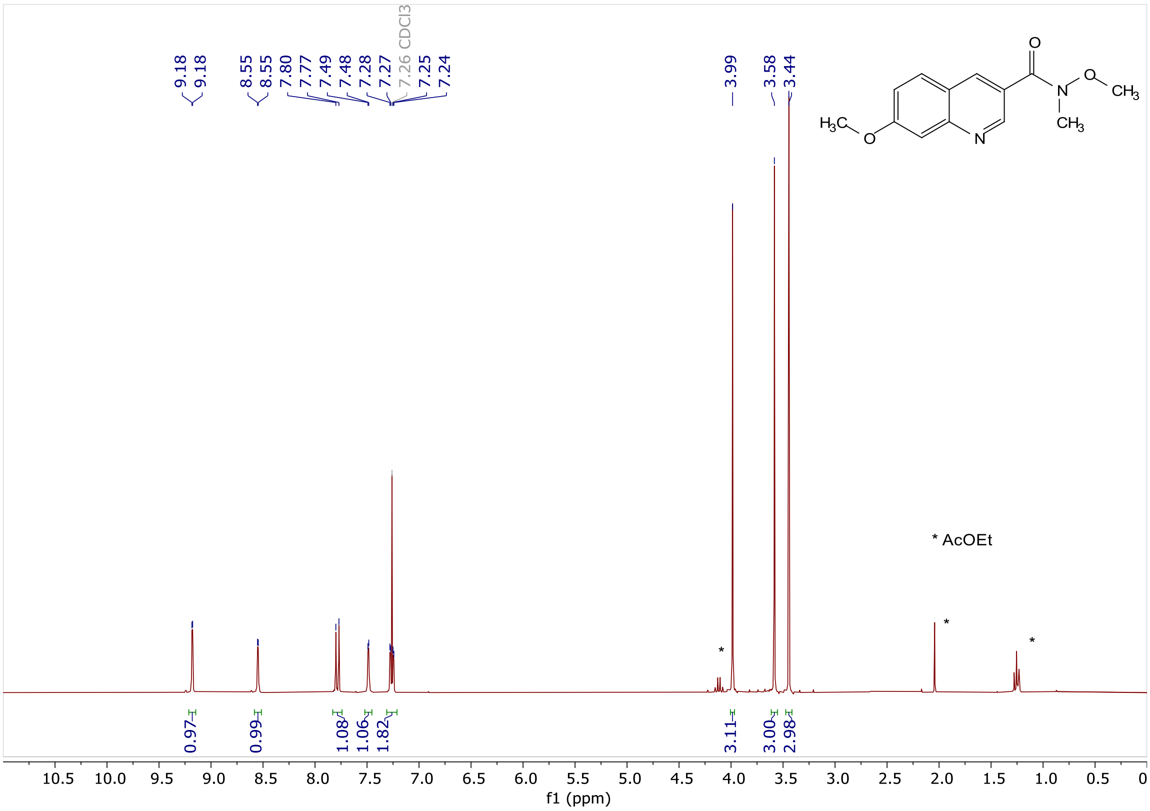

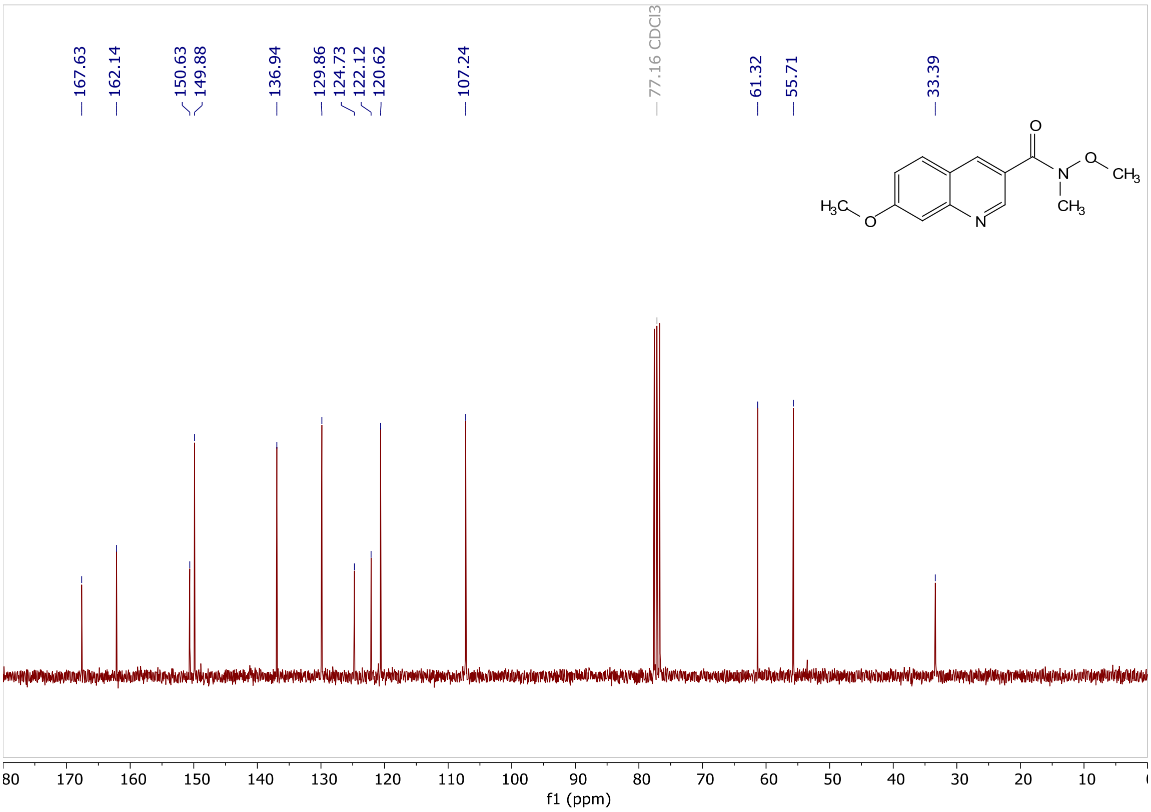

Supplement: Supplementary file 1 [file molecules-28-00036-s001.zip › molecules-2084357-supplementary.pdf]
